# The Origin of Anion−π Autocatalysis

**DOI:** 10.1021/jacsau.2c00656

**Published:** 2023-03-17

**Authors:** M. Ángeles Gutiérrez López, Mei-Ling Tan, Antonio Frontera, Stefan Matile

**Affiliations:** † Department of Organic Chemistry, University of Geneva, CH-1211 Geneva, Switzerland; ‡ National Centre of Competence in Research (NCCR) Molecular Systems Engineering (MSE), CH-4002 Basel, Switzerland; § Departament de Química, Universitat de les Illes Balears, SP-07122 Palma de Mallorca, Spain

**Keywords:** anion−π interactions, anion−π catalysis, autocatalysis, water catalysis, cascade cyclizations, epoxides, polyethers

## Abstract

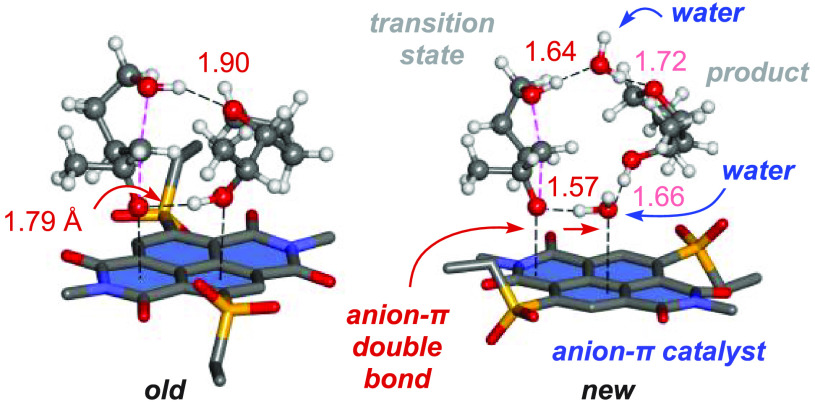

The autocatalysis of epoxide-opening ether cyclizations on the aromatic surface of anion−π catalysts stands out as a leading example of emergent properties expected from the integration of unorthodox interactions into catalysis. A working hypothesis was proposed early on, but the mechanism of anion−π autocatalysis has never been elucidated. Here, we show that anion−π autocatalysis is almost independent of peripheral crowding in substrate and product. Inaccessible asymmetric anion−π autocatalysis and sometimes erratic reproducibility further support that the origin of anion−π autocatalysis is more complex than originally assumed. The apparent long-distance communication without physical contact calls for the inclusion of water between substrate and product on the catalytic aromatic surface. Efficient anion−π autocatalysis around equimolar amounts but poor activity in dry solvents and with excess water indicate that this inclusion of water requires high precision. Computational models suggest that two water molecules transmit dual substrate activation by the product and serve as proton shuttles along antiparallel but decoupled hydrogen-bonded chains to delocalize and stabilize evolving charge density in the transition state by “anion−π double bonds”. This new transition-state model of anion−π autocatalysis provides a plausible mechanism that explains experimental results and brings anion−π catalysis to an unprecedented level of sophistication.

## Introduction

**Figure 1:**
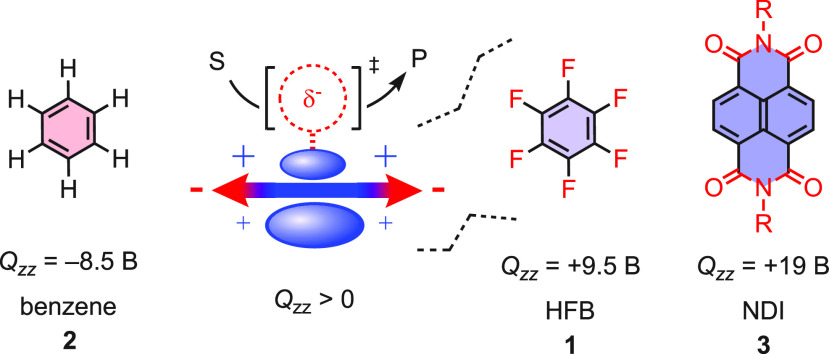
Anion−π catalysis is conceived as stabilization of anionic transition states on polarizable (blue ellipses) aromatic surfaces with withdrawing substituents (red arrows) to access positive quadrupole moments *Q*_*zz*_ (+, −, + ); with *Q*_*zz*_ for classical π acids (**1**, **3**) and π bases (**2**).

Cation−π catalysis, that is, the stabilization of cationic reactive intermediates such as carbocations on π–basic aromatic surfaces, is ubiquitous in biology.^1,2^ The most spectacular example is steroid cyclization, which nicely illustrates how the delocalized nature of cation−π interactions can be advantageous to stabilize intermediates and transition states that involve long-distance charge displacements.^3^ In small-molecule organocatalysis, the integration of cation−π interactions is receiving increasing appreciation.^1,2,4−7^ The complementary anion−π catalysis, that is, the stabilization of anionic transition states and reactive intermediates on π-acidic aromatic surfaces (Figure 1), is rare in nature and has, like the anion−π interaction itself, been ignored for a long time in chemistry.^8−11^ This is in part understandable because the attraction of anions to aromatic surfaces is counterintuitive. One way to achieve this inversion of selectivity is to invert the quadrupole moment *Q*_*zz*_ perpendicular to the π surface from *Q*_*zz*_ < 0 to *Q*_*zz*_ > 0 (Figure 1).^8^ This is achieved by attaching electron-withdrawing substituents to the aromatic plane, which also strengthens outward in-plane dipoles that enhance the affinity of anions rather than cations (Figure 1). In addition, the aromatic system can be polarized transiently to induce and increase anion−π interactions.

The archetypal aromatic system capable of anion−π interactions is hexafluorobenzene (HFB, **1**, Figure 1). The *Q*_*zz*_ = +9.5 B (Buckinghams) is in the range of benzene (**1**, *Q*_*zz*_ = −8.5 B) but opposite in sign. Naphthalenediimides (NDIs) **3** have been identified early on as a privileged platform for anion−π interactions because the intrinsic *Q*_*zz*_ = +19 B is very high and can be further increased strongly by adding electron-withdrawing substituents.^8^

**Scheme 1:**
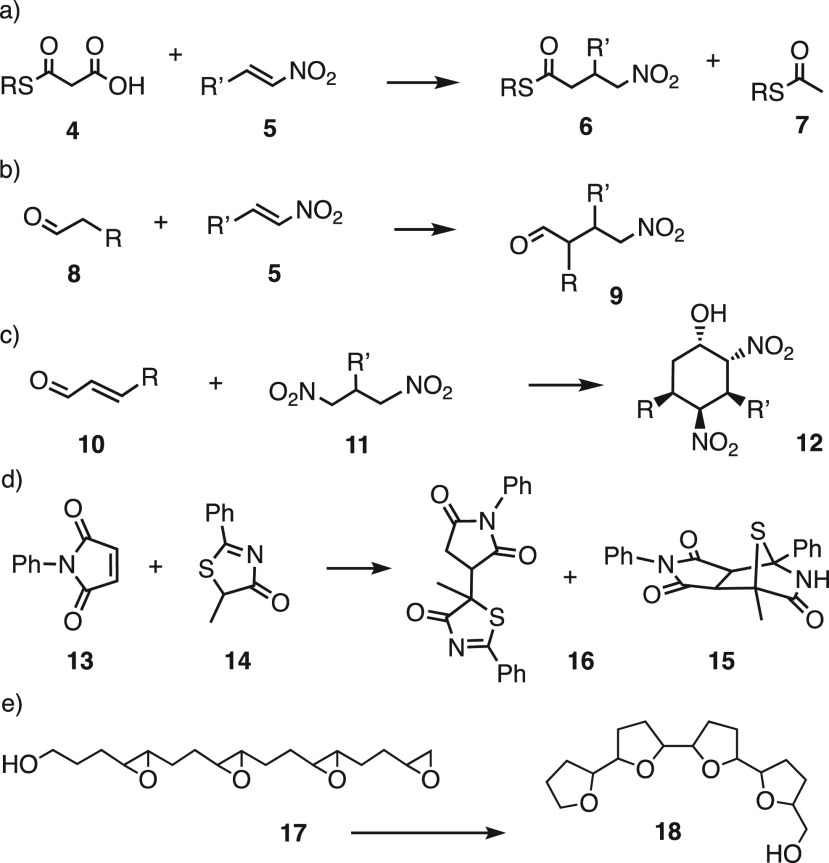
Selected Reactions Realized with Anion−π Catalysis Key: (a) enolate addition, (b) enamine, (c) iminium, (d) Diels–Alder, and (e) cascade cyclization chemistry. See the text for comments and references.

Although reported first in 2013,^12^ the true beginning of anion−π catalysis is arguably in 2015 because it took some time to find a good reaction for detection.^13^ The best benchmark reaction turned out to be the addition of malonic half-thioesters **4** to nitroolefins **5** (Scheme 1). Stabilization of both the enolate and the nitronate intermediates on aromatic surfaces with a positive quadrupole moment accelerates enolate addition and decelerates decarboxylation, thus affording the disfavored product **6** rather than the intrinsically favored product **7**.^13^

With the enolate addition chemistry in place, anion−π catalysts up to π-stacked foldamers, fullerenes, and carbon nanotubes could be developed, which then were used to realize different reactions (Scheme 1).^8^ Examples include the enantioselective addition of enamines from aldehyde **8** to nitroolefins **5** to generate two stereogenic centers in aldehyde **9**. Iminium chemistry was introduced to asymmetric anion−π catalysis with a Jørgenson reaction that cyclizes the acyclic achiral enal **10** and substrate **11** into cyclohexane **12** with five stereogenic centers. With anion−π catalysis, anionic Diels–Alder cycloadditions with **13** and **14** give the disfavored *exo* product **15** with high selectivity (88% yield, 94% ee, > 20:1 dr), because the otherwise favored *endo* transition state is less accessible for stabilizing π surfaces. Conventional organocatalysts give the Michael addition product **16** as main product instead.^8^

The delocalized nature of anion−π interactions suggests that reactions involving long-range charge displacement could particularly benefit from anion−π catalysis. This hypothesis is supported by examples from the charge-inverted, orthodox cation−π catalysis, with the stabilization of carbocation intermediate on π-basic aromatic residues during steroid cyclization being arguably the most spectacular example.^3^ The search for an anionic counterpart of steroid cyclizations led us to consider polyether cascade cyclizations to elaborate on transfer of electrons rather than holes over long distances with anion−π catalysis.^14−16^ Charismatic in chemistry and biology, these giant cascades, forming ten and more fused cyclic ethers in one step, have been explored by many giants in organic chemistry.^17−33^

**Figure 2:**
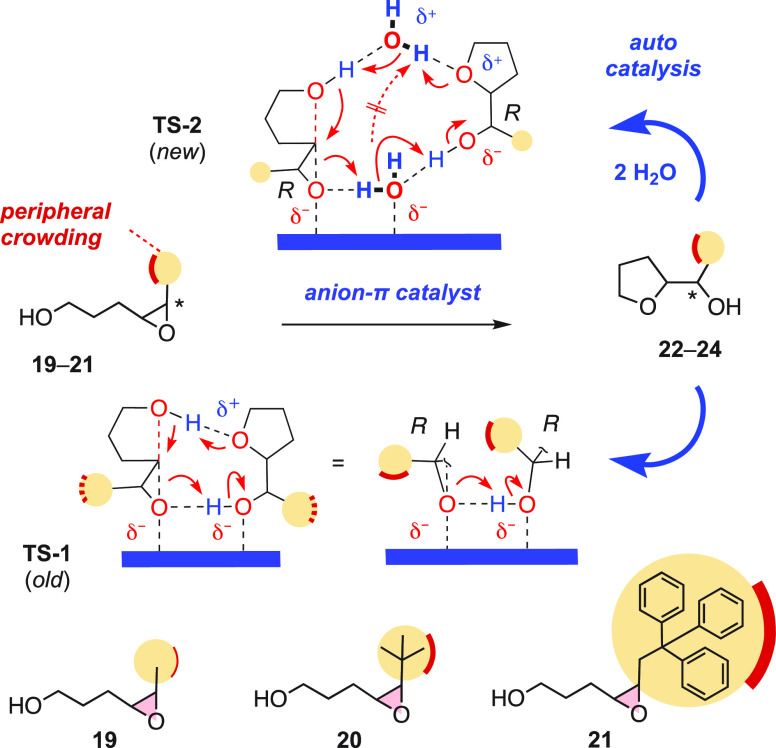
Peripheral crowding (orange circles) introduced to probe the original mechanism of autocatalysis of epoxide-opening ether cyclizations on anion−π catalysts in aprotic solvents (**TS-1**) and a proposed revised transition state **TS-2** including two molecules of water. For the original **TS-1**, computational models indicate matched crowding for the *R* substrate and *R* product.

On π-acidic surfaces, polyether cascades have been realized so far up to the cyclization of tetra-epoxide **17** into tetra-THF **18** (Scheme 1).^14−16^ These cascades occurred also in the absence of a secondary activation group attached to the active π surface, like a Brønsted base, and showed autocatalytic behavior (Figure 2).^14−16^ This behavior is unique for anion−π catalysis, a leading example for the emergent properties that are expected from the integration of unorthodox interactions in catalysis. While molecular models and working hypothesis **TS-1** were quickly realized (Figure 2),^14^ the molecular basis of anion−π autocatalysis has remained unexplored, and efforts toward asymmetric autocatalysis^34−42^ have not been fruitful so far.^43^ To elucidate the mechanism of anion−π autocatalysis, substrates **19**–**21** with increasing peripheral crowding will be introduced in the following. Based on the results, a new **TS-2** will be proposed to account for anion−π autocatalysis (Figure 2). This new **TS-2** contains two molecules of water sandwiched between substrate and product on the π-acidic aromatic surface to form two short hydrogen-bonded chains that transmit activation of nucleophile and leaving group by the product. Moreover, the two catalytic water molecules delocalize the positive and negative charge generated during the cyclization, the latter being stabilized by a formal “anion−π double bond”.

Peripheral crowding was introduced by Whitesides and co-workers in 1994 to enable the self-assembly of supramolecular rosettes.^44^ In brief, physical repulsion between bulky substituents added at the periphery remotely controls the structure and function at the active center. For the self-assembly of supramolecular rosettes, the challenge is to suppress the competing linear assembly into supramolecular polymers that precipitate and thus shift the coupled equilibria in their favor. Peripheral crowding added to the monomeric sectors elegantly solved this problem, with physical repulsion occurring only in the linear supramolecular polymers but not in the circular rosettes. Without being explicitly named as such, peripheral crowding can be seen at work contributing to structure and function of many molecular and supramolecular systems. In this study, peripheral crowding was considered to elucidate the mechanism of autocatalysis on the π-acidic aromatic surface of anion−π catalysts (Figure 2).

The results from peripherally crowded substrates **19**–**21** indicate that the presence of two molecules of water included between substrate and product on the π-acidic surface is needed for operational anion−π autocatalysis. For enzymatic catalysis, such participation of water has been widely supported from different points of view, with the impact being more often on protein structure than direct participation in the transformation, unless, of course, water is one of the substrates.^45−49^ In organic synthesis, reactions in or “on” water have received much attention, with often spectacular rate enhancements being observed, first with Diels–Alder reactions, then many more.^50−54^ In the context of this study, epoxide-opening polyether cascade cyclizations in water are most relevant. With an essential intramolecular tetrahydropyran template and water as a solvent, two water molecules were proposed to interact with the template to enforce the conformational changes needed to activate the nucleophile by proton transfer.^18,19^ In heterogeneous catalysis, water bound to the surface of the solid catalyst often contributes to activity.^50,55^

Examples of stoichiometric amounts of water integrated as co-catalysts in apolar solvents are less ubiquitous and usually well hidden in the literature. A topical example concerns the epoxide-opening cyclization of 4,5-epoxyhexanols with methoxymethyl substituents on carbon 4 to coordinate the lanthanoid Lewis acid catalyst La(OTf)_3_.^20,56^ This cyclization worked best with 2 equiv of water. The authors suggested water to coordinate to the large metal and increase the coordination number to preorganize the system for the observed endo regioselectivity, but direct participation in the cyclization has not been excluded (*vide infra*). Closely related to the present topic, anion−π interactions acidifying trace amounts of water or other weak acids, possibly DMSO, for proton transfer to fluoride anions have been evoked to explain the reduction of NDIs in the presence of fluoride, which is not a reducing agent.^57^ In supramolecular catalysis, capsules integrating water into their architecture provide a superb example of this situation.^5,58−61^ Extensive studies on this topic also illustrate the analytical challenge of detecting, characterizing, and understanding the role of active monomeric or oligomeric water in supramolecular catalysts on the molecular level. In the following, water emerges as a co-catalyst in anion−π autocatalysis in aprotic solvents like dichloromethane.

## Results and Discussion

### Design

One of the simplest substrates for autocatalysis on π-acidic surfaces is 4,5-epoxyhexanol **19** (Figure 2).^43^ The epoxide is in the *cis* configuration and is used as a racemic mixture of enantiomers. Epoxide-opening *exo-tet* ether cyclization affords the tetrahydrofuran (THF) or oxolane **22**, which then catalyzes its own formation. In computational models of the autocatalytic transition state **TS-1**, substrate and product bind to the π-acidic surface with lone-pair π interactions. Upon epoxide opening, the released alcoholate engages in shorter anion−π interactions. The product accepts and donates one hydrogen bond each, activating both nucleophile and leaving group of the substrate.

In computational models of the original **TS-1** (see below),^14^ the methyl groups of the *R* substrate **19** and its *R* product **22** are matched (Figure 2). Increasing peripheral crowding at this position could, if **TS-1** is correct, perhaps lead to kinetic resolution through enantioselective substrate recognition by chiral products before completely abolishing autocatalysis.

### Synthesis

**Scheme 2:**
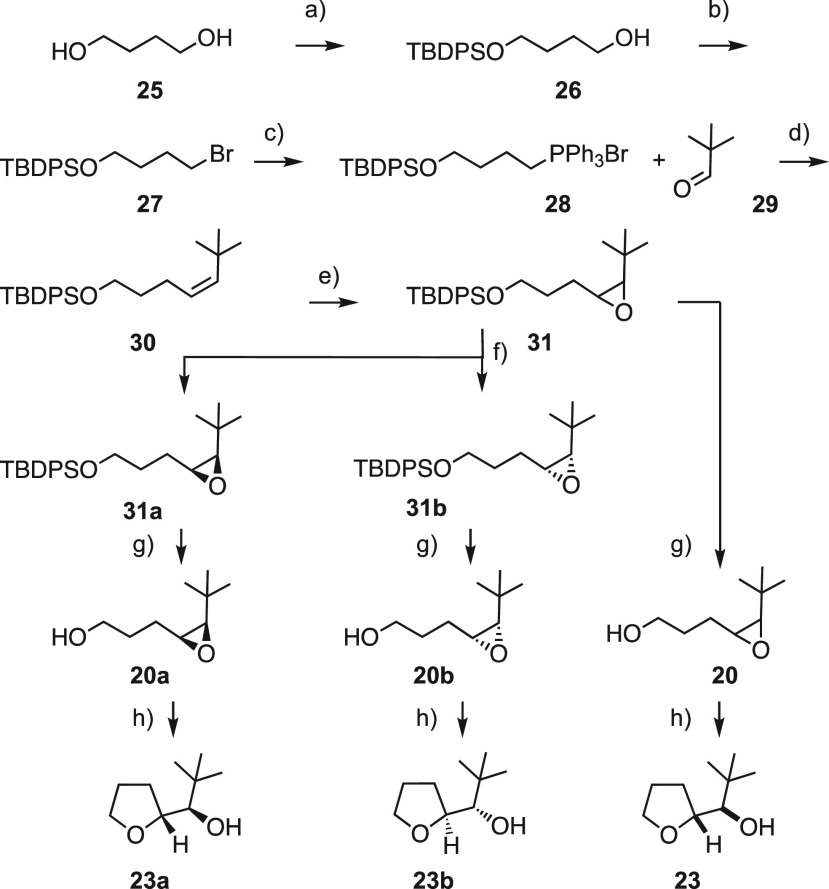
Substrate Synthesis Key: (a) NaH, TBDPSCl, THF, 0 °C, 2.5 h, quant; (b) CBr_4_, PPh_3_, CH_2_Cl_2_, 0 °C to rt, 2 h, 67%; (c) PPh_3_, toluene, 150 °C, 15 h, 65%; (d) (1) LiHDMS, THF, −78 °C, 0.5 h, (2) **29**, −78 °C to rt, 15 h, 79%; (e) mCPBA, CH_2_Cl_2_, 0 °C to rt, 15 h, 91%; (f) chiral HPLC, 89% ee (**31a**), 96% ee (**31b**); (g) TBAF, THF, 0 °C to rt, 2 h, 89% (**20**), 85% (**20a**), 83% (**20b**); (h) SbCl_3_, CD_2_Cl_2_, rt, 2 h, 85% (**23**), 75% (**23a**), 87% (**23b**). While **31a**, **31b**, **20a**, **20b**, **23a**, and **23b** are enantioenriched, their absolute configuration is unknown.

To elaborate on peripheral crowding as a tool to elucidate the mechanism of asymmetric anion−π autocatalysis, epoxides **20** and **21** were considered. Both were synthesized from diol **25** (Scheme 2). To prepare for the central Wittig reaction, silyl protection of one alcohol gave **26** and bromination of the other gave **27**, which was converted into the triphenyl phosphonium salt **28**. For the *tert*-butylated epoxide **20**, pivalaldehyde **29** was used for the Wittig reaction, which was controlled to give the *cis* olefin **30**. Epoxidation with mCPBA gave *cis* epoxide **31**, which was deprotected to yield the desired substrate **20** as a racemic mixture of enantiomers. For studies on stereoselectivity, the two enantiomers of **31** were separated by chiral HPLC into enantioenriched **31a** with 89% ee and **31b** with 96% ee. Deprotection yielded enantiomers **20a** and **20b**, which were cyclized with SbCl_3_^62^ into the respective enantioenriched products **23a** and **23b** of unknown absolute configuration. Epoxide **21** was synthesized analogously using the corresponding triphenylpropanal for the Wittig reaction (Scheme S4).

### Anion−π Autocatalysis, Solvent Dependence

**Figure 3:**
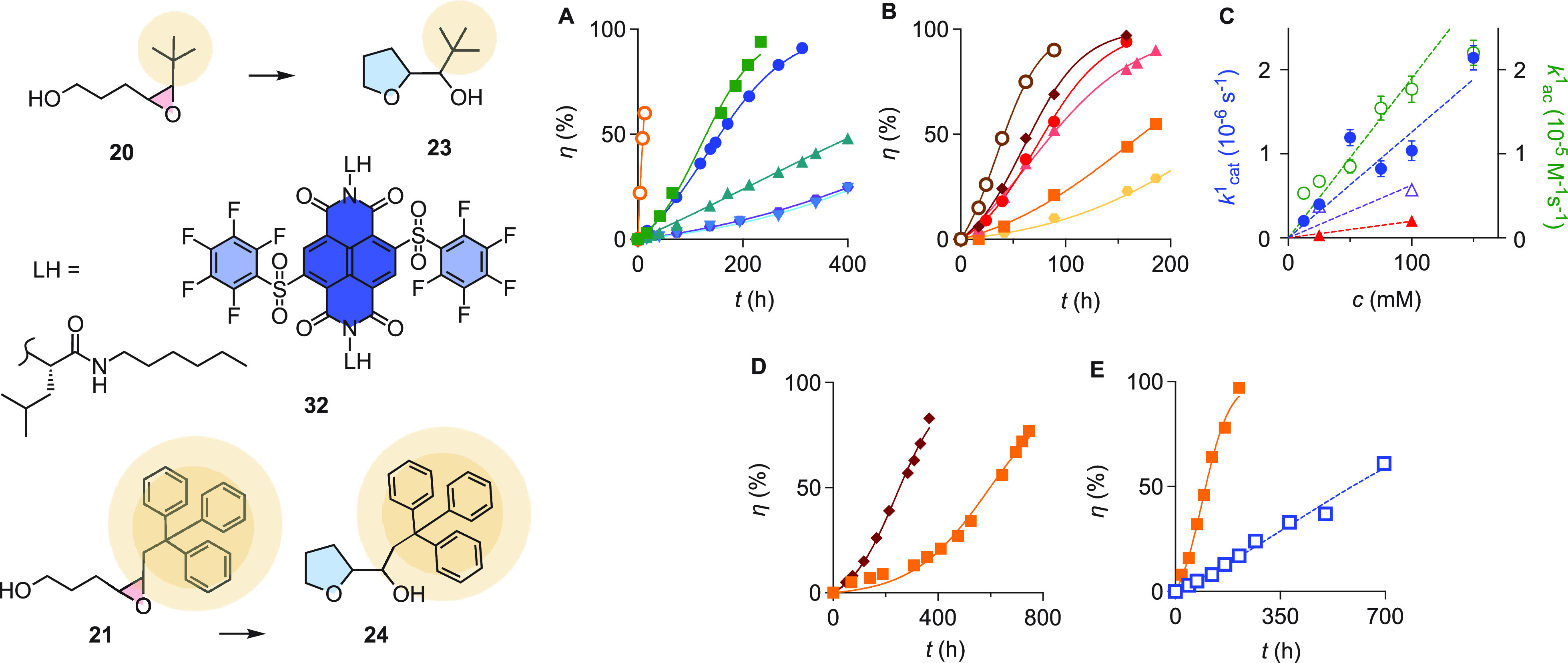
(A) Time course of the conversion η of **20** (840 mM) with **32** (42 mM, 5 mol %) in C_6_F_6_ (orange ○), CD_2_Cl_2_ (green square), DMSO-*d*_6_ (blue circles), CD_3_CN (teal upward triangles), acetone-*d*_6_ (purple hexagons), and THF-*d*_6_ (pale blue downward triangles). (B) Time course of the conversion η of **20** (500 mM) in the presence of catalyst **32** (12.5, 25, 50, 75, 100, and 150 mM, pale orange to brown) in CD_2_Cl_2_ at rt. (C) Pseudo-first-order initial (*k*^1^_cat_, filled symbols) and autocatalytic rate constants (*k*^1^_ac_, open symbols) of catalytic reactions with varying concentrations of catalyst **32** with **20** (circles) or **21** (triangles, both 500 mM); error bars represent SEMs of the best fits. (D) Time course of the conversion η of **21** (500 mM) in the presence of 25 (orange squares) and 100 mM **32** (dark red diamonds) in CD_2_Cl_2_ at rt. (E) Time course of the conversion η of **21** (500 mM) in the presence of **32** (25 mM) in C_6_F_6_/CD_2_Cl_2_ 4:1 at rt (orange filled squares) and 10 °C (blue empty squares).

Anion−π autocatalytic ether cyclization of the peripherally crowded epoxide **20** was explored first with 42 mM or 5 mol % of the powerful NDI catalyst **32** (Figure 3). Sigmoidal kinetics consistent with autocatalysis were observed in most aprotic solvents (Figure 3A). Non-autocatalytic initial and autocatalytic rate constants, *k*_cat_ and *k*_ac_, respectively, were estimated by fitting the data to the autocatalysis rate equation assuming pseudo-first order conditions to give *k*^1^_cat_ and *k*^1^_ac_ and then dividing by the catalyst concentration (eqs S1–S4). Fitting the data for CD_2_Cl_2_ to the autocatalysis rate equation gave *k*_cat_ = 1.2 ± 0.3 × 10^–5^ M^–1^ s^–1^ and *k*_ac_ = 1.3 ± 0.2 × 10^–4^ M^–2^ s^–1^ and, thus, an autocatalytic rate enhancement *k*_ac_/*k*_cat_ ≈ 11 M^–1^ (Figure 3A, green squares, Table 1, entry 1). Catalytic activity decreased with increasing Lewis basicity of the solvent from CD_2_Cl_2_ (10.0 kJ mol^–1^ for complexing BF_3_, *t*_1/2_ ≈ 130 h)^63^ over CD_3_CN (60.4 kJ mol^–1^, teal upward triangles, *t*_1/2_ ≈ 400 h) to acetone*-d*_6_ (76.0 kJ mol^–1^, purple hexagons, *t*_1/2_ ≫ 400 h) and THF*-d*_8_ (90.4 kJ mol^–1^, pale blue downward triangles, *t*_1/2_ ≫ 400 h). Increasing activity with decreasing solvent basicity suggested that these nonprotic solvents compete with substrate and product for lone-pair−π interactions on catalyst **32**.

**Table 1:** Rate Constants for Anion−π Catalysis and Autocatalysis^a^

	C^b^	S^c^	Solvent	*k*_cat_^d^ (10^–5^ M^–1^ s^–1^)	*k*_ac_^e^ (10^–4^ M^–2^ s^–1^)	*k*_ac_/*k*_cat_^f^ (M^–1^)
1	**32**	**20**	CD_2_Cl_2_	1.2 ± 0.3	1.3 ± 0.2	11 ± 4
2	**32**	**20**	CD_3_CN	0.7 ± 0.04	0.2 ± 0.03	2.5 ± 0.6
3	**32**	**20**	(CD_3_)_2_CO	0.2 ± 0.02	0.3 ± 0.03	13 ± 2
4	**32**	**20**	THF-*d*_8_	0.2 ± 0.02	0.3 ± 0.1	20 ± 6
5	**32**	**20**	DMSO-*d*_6_	1.3 ± 0.1	0.9 ± 0.03	6.6 ± 0.5
6	**32**	**20**	C_6_F_6_	44 ± 8	2 ± 3	0.4 ± 0.8
7^g^	**32**	**20**	CD_2_Cl_2_	1.6 ± 0.2	2.7 ± 0.2	17 ± 3
8^g^	**32**	**21**	CD_2_Cl_2_	0.3 ± 0.1	3.8 ± 0.2	120 ± 30
9^g^	**32**	**21**	C_6_F_6_/CD_2_CL_2_^*h*^	2.9 ± 0.4	4.4 ± 0.5	15 ± 4
10^g^	**33**	**20**	CD_2_Cl_2_	0.3 ± 0.02	0.8 ± 0.1	28 ± 4
11^g^	**34**	**20**	DMSO-*d*_6_	0.2 ± 0.02	0.6 ± 0.04	27 ± 4
12^g^	**35**	**20**	CD_2_Cl_2_	1.4 ± 0.2	0.6 ± 0.2	4 ± 2

a Conditions: substrate (**20**) 840 mM, catalyst (**32**) 42 mM, rt. For complete data with errors, see the Supporting Information.

b Catalysts (Figures 2 and 3).

c Substrates (Figure 2).

d Nonautocatalytic rate constant for substrate consumption (eqs S1–S4).

e Autocatalysis rate constant (eqs S1−S4).

f Autocatalytic rate enhancement (eqs S1–S4).

g Substrates (**20** or **21**) 500 mM, catalysts (**32**–**34**) 25 mM.

The exception from this trend was autocatalysis in DMSO-*d*_6_, which was as good as in CD_2_Cl_2_ despite maximal Lewis basicity (105.3 kJ mol^–1^, Figure 3A, blue filled circles, Table 1, entry 5, *t*_1/2_ ≈ 150 h). These results implied that DMSO-*d*_6_ is acting more like a co-catalyst than as an inhibitor, probably by simultaneous lone-pair−π interactions and activation of the nucleophile at the sulfur and oxygen atoms.^64^ This interpretation was consistent with reduced autocatalysis in DMSO-*d*_6_ compared to CD_2_Cl_2_ (*k*_ac_ = 0.9 ± 0.03 × 10^–4^ M^–2^ s^–1^, Δ*k*_ac_ = −40%, Figure 3A, Table 1, entry 1 vs 5). Residual autocatalysis suggested that π-surface-bound product **23** can activate the nucleophile and leaving group as outlined in **TS-1** better than DMSO-*d*_6_ (Figure 2).

Exceptional rate enhancements without much autocatalysis were found with catalyst **32** in hexafluorobenzene (*t*_1/2_ ≈ 10 h, *k*_ac_/*k*_cat_ ≈ 0.4 M^–1^, Figure 3A, orange empty circles, Table 1, entry 6). Unlike epoxide **19**, the cyclization of crowded epoxide **20** was not catalyzed by hexafluorobenzene alone without catalyst **32**. The important activation of anion−π catalyst **32** in hexafluorobenzene will be discussed later.

### Concentration Dependence

The velocity of product formation from 500 mM substrate **20** increased with increasing concentration of catalyst **32**, and *t*_1/2_ decreased from 10 days at 12.5 mM or 2.5 mol % to 1 day at 150 mM or 30 mol % catalyst (Figure 3B). The pseudo-first-order *k*^1^_cat_ (Figure 3C, blue filled circles) and *k*^1^_ac_ (Figure 3C, green empty circles) were linearly dependent on the catalyst concentration, as expected. Catalyst concentrations of 25 mM for 500 mM of substrate, that is 5 mol %, were chosen for further studies because of lower error levels.

### Peripheral Crowding

Increasing peripheral crowding with triphenylethyl in epoxide **21** decelerated cyclization on anion−π catalyst **32** in CD_2_Cl_2_. At 25 mM or 5 mol % catalyst **32**, *t*_1/2_ = 6.8 days for *tert*-butyl epoxide **20** increased to *t*_1/2_ = 25 days for epoxide **21** (Figure 3D, orange squares). At 100 mM or 20 mol % catalyst, the substrate half-life shortened to *t*_1/2_ = 10.5 days, still significantly longer compared to *t*_1/2_ = 2.8 days for **20** (Figure 3D, dark red diamonds). With 5 mol % catalyst **32** in hexafluorobenzene/CD_2_Cl_2_ 4:1, important rate enhancements were reproduced with substrate **21** at high crowding (Figure 3E). Half-lives shortened from *t*_1/2_ = 25 days to *t*_1/2_ = 4.2 days, compared to *t*_1/2_ = 10 h for **20** under similar conditions. At low temperature, *t*_1/2_ increased back to 25 days.

Autocatalytic behavior was preserved with the supercrowded substrate **21**. At low catalyst concentration in CD_2_Cl_2_, rate enhancements *k*_ac_/*k*_cat_ = 120 ± 30 M^–1^ were more significant than with substrate **20** (Figure 3D, Table 1, entry 8). Under faster conditions with more catalyst (*k*_ac_/*k*_cat_ = 29 ± 5 M^–1^) and even in hexafluorobenzene/CH_2_Cl_2_ 4:1, residual autocatalysis remained (*k*_ac_/*k*_cat_ = 15 ± 4 M^–1^, Figure 3E, Table 1, entry 9).

Ether cyclization of the original epoxide **19** measured at a higher concentration (0.84 M) with catalyst **32** (0.17 M) gave *t*_1/2_ < 2 days and *k*_ac_/*k*_cat_ ≈ 20 M^–1^.^14^ Increasing peripheral crowding from methyl in **19** to *tert*-butyl in **20** thus had marginal effects on half-life or autocatalysis. However, a further increase in peripheral crowding from *tert-*butyl in **20** to triphenylethyl in **21** deteriorated the activity with about 4 times larger *t*_1/2_ and 5 times smaller *k*_cat_ but without much effect on *k*_ac_. Increasing *t*_1/2_ and decreasing *k*_cat_ with increasing peripheral crowding were not surprising and can have many origins also unrelated to anion−π catalysis such as, for example, intrinsic reactivity. In contrast, essentially preserved autocatalysis *k*_ac_ was unexpected and was inconsistent with the working model **TS-1**, which would predict disappearance of autocatalysis with increasing peripheral crowding (*vide infra*).

### Catalyst Variations

**Figure 4:**
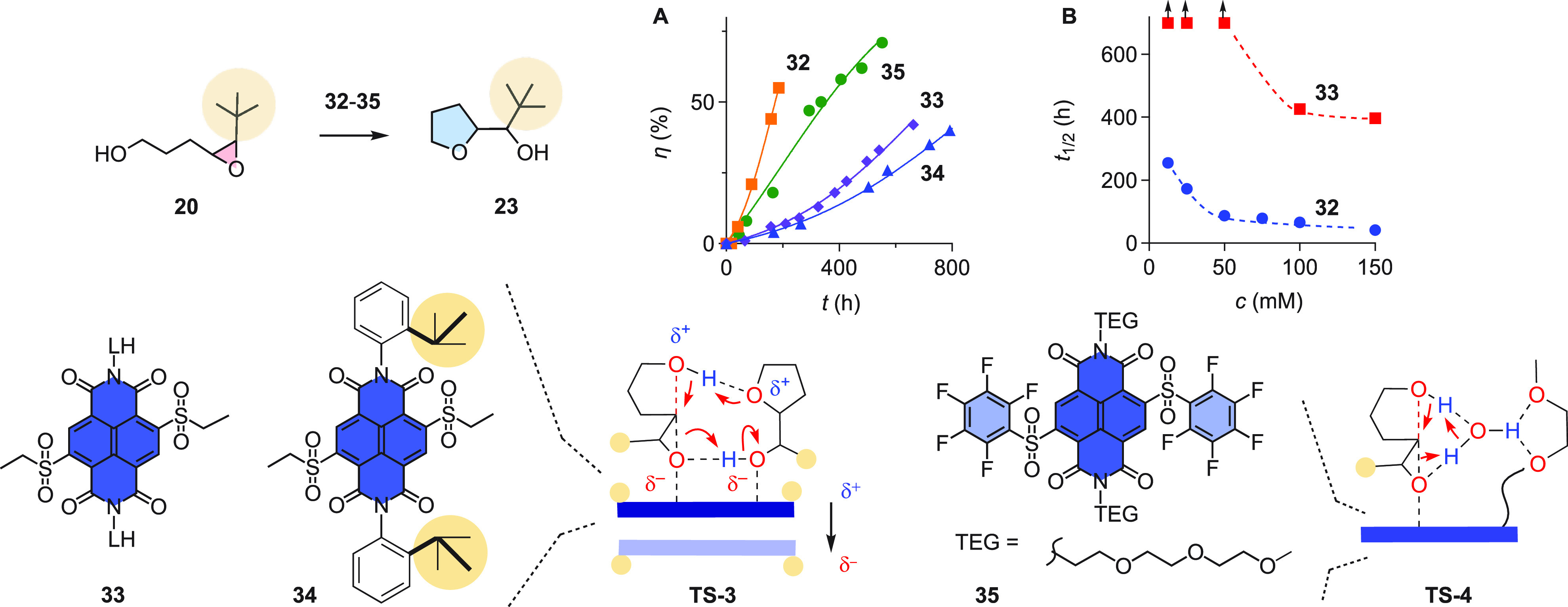
Structures and catalytic activities of catalysts **33**–**35** with notional **TS-3** of catalyst **34** dimers with increased polarizability (black arrow) and peripheral crowding (golden circles) and notional **TS-4** with substrate activation by TEG and, perhaps, water on the anion−π catalyst **35** (instead of autocatalysis in **TS-1**). (A) Kinetics of the conversion η of **20** (500 mM) in the presence of 25 mM (5 mol %) catalyst **32** (orange squares), **33** (purple diamonds), **34** (blue triangles), and **35** (green circles) in CD_2_Cl_2_ (**32**, **33**, and **35**) or DMSO-*d*_6_ (**34**) at rt. (B) Dependence of half-lives (*t*_1/2_) on the concentrations of catalysts **32** and **33**.

Catalyst **33** with two ethyl sulfones in the NDI core has been reported previously (Figure 4).^14^ This catalyst **33** in CD_2_Cl_2_ cyclized substrate **20** only reluctantly (Figure 4A, purple diamonds). To reach a reasonable *t*_1/2_ = 18 days, 100 mM or 20 mol % catalyst was needed. This compared to *t*_1/2_ = 2.8 days for catalyst **32** with two pentafluorophenylsulfones in the NDI core, that is, Δ*t*_1/2_ = 15 days (Figure 4B). Initial rates decreased correspondingly from *k*_cat_ = 1.6 ± 0.2 × 10^–5^ M^–1^ s^–1^ for **32** to *k*_cat_ = 0.3 ± 0.03 × 10^–5^ M^–1^ s^–1^ for **33**, that is, a decrease by 80% at 5 mol % catalyst. This decreasing activity was consistent with decreasing π acidity of the catalysts. LUMO energies have been shown to increase from −4.73 to −4.52 eV upon replacement of the electron-withdrawing pentafluorophenyl in **32** with the ethyl groups in **33**.^65^ Moreover, the pentafluorophenyl groups offer additional π surface for anion−π catalysis, although they are clearly smaller and less π acidic. Despite much slower conversion, catalyst **33** showed preserved autocatalysis (Figure 4A, purple diamonds, Table 1, entry 10, *k*_ac_/*k*_cat_ = 28 M^–1^).

Catalyst **34** with two ethyl sulfones in the NDI core and peripheral crowding at the imides was prepared during the early failed attempts to introduce anion−π catalysis (Figure 4).^66^ Without the secondary functional groups needed at that time, it was not further pursued.^8^ However, since anion−π catalysis of ether cyclization does not require secondary activation,^14^ catalyst **34** deserved reconsideration. The peripheral crowding is attractive because the *tert*-butyl groups prevent rotation around the phenyl-NDI bonds, which allows the isolation of atropisomers.

In the present context, only the *cis* isomer **34** was of interest and used as a mixture of enantiomers. These *cis* isomers have been shown to dimerize under appropriate conditions.^66^ Such dimers were considered for anion−π catalysis because through-space polarizability of face-to-face π stacks strongly increased anion−π catalysis on π-stacked foldamers,^67^ while peripheral crowding of the catalyst could enhance the impact of peripheral crowding of the substrates, as outlined in **TS-3** (Figure 4). However, the catalytic activity of **34**, measured for solubility reasons in DMSO-*d*_6_ (Figure 3A), was with *k*_cat_ = 0.2 ± 0.02 × 10^–5^ M^–1^ s^–1^ slightly weaker than **33** (Figure 4A, blue triangles, Table 1, entry 11 vs 10). This disappointing result left room for interpretations that have not been further developed because the poor activity dwarfed their significance.

Catalyst **35** was newly synthesized (Figure 4). Like **32**, **35** contains the active pentafluorophenylsulfones in the NDI core and product mimicking TEG substituents on the imides. Under standard conditions at 25 mM or 5 mol % **35** in CD_2_Cl_2_, epoxide **20** cyclized in *t*_1/2_ = 15 days and *k*_cat_ = 1.4 ± 0.2 × 10^–5^ M^–1^ s^–1^ (Figure 4A, green circles). Compared to **32**, *t*_1/2_ was 8 days longer, mainly due to the ∼5 times lower *k*_ac_, while *k*_cat_ was only slightly weaker (Table 1, entry 12 vs 7). This reduced autocatalysis was consistent with the TEG acting as a covalent product mimic that is good enough not to be replaced by the emerging product during the reaction. The longer *t*_1/2_ indicated that the covalent product mimic in **35** is not as active as the product itself as a co-catalyst. This was reasonable because it misses the hydrogen-bond donor to activate the leaving group. As outlined in **TS-4**, this could possibly occur intermolecularly with the integration of one molecule of water as a proton shuttle (Figure 4, *vide infra*).

### Co-Catalysts

**Figure 5:**
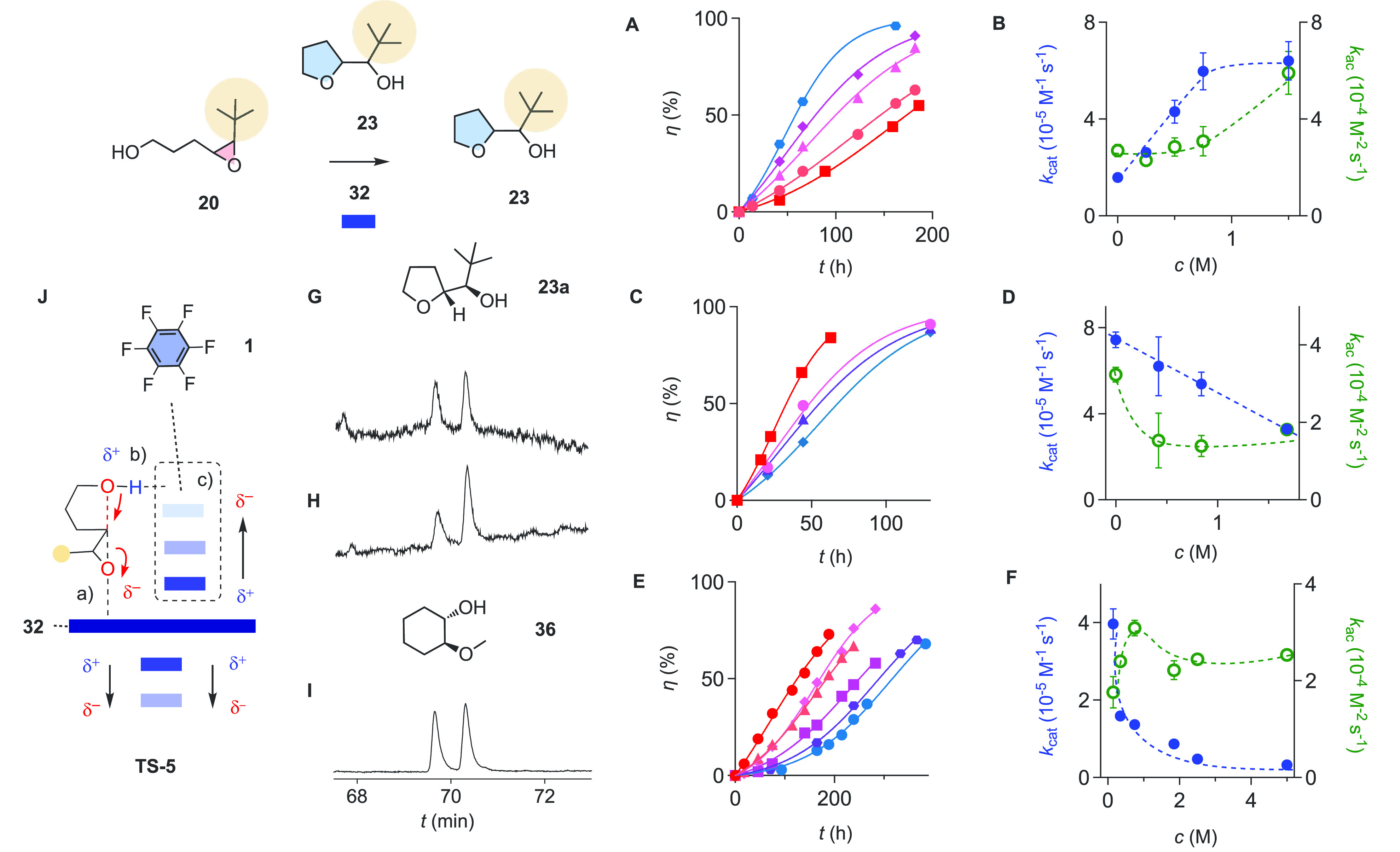
(A) Conversion η of substrate **20** (500 mM) with catalyst **32** (25 mM) and product **23** as co-catalyst (0–1.5 M, red to blue) with time in CD_2_Cl_2_ at rt. (B) Dependence of initial rate (*k*_cat_) and autocatalytic rate (*k*_ac_) on the concentration of **23**. (C) η of **20** (840 mM) with **32** (42 mM) and **23** (0–1.66 M, red to blue) with time in **1** at 10 °C. (D) Dependence of *k*_cat_ and *k*_ac_ on the concentration of **23**. (E) η of **20** (500 mM) with **32** (25 mM) and co-catalyst **36** (0.15–5.0 M, red to blue) with time in **1** at 10 °C. (F) Dependence of *k*_cat_ and *k*_ac_ on the concentration of **36**. (G) Chiral GC trace of residual **20** during reaction with **32** and enantioenriched **23a** (0.38 M; 0.75 equiv) in CD_2_Cl_2_, rt, at 94% conversion. (H) Same for 3.0 equiv of **23a** (1.5 M) at 10 °C, 80% conversion. (I) Same for 1.5 equiv of **36** (0.75 M) at 10 °C, 86% conversion. (J) Notional **TS-5** of anion−π catalyst **32** with stacked C_6_F_6_ to (a) induce π acidity (dark blue) by increasing polarizability (black arrows), (b) perhaps activate the nucleophile, and (c) obstruct autocatalysis (**TS-1**, Figure 2).

The addition of product **23** at the beginning of the reaction accelerated the cyclization of **20** with the best catalyst **32** in CD_2_Cl_2_ significantly (Figure 5A, red to blue). These rate enhancements caused by the presence of the product at the beginning of the reaction are characteristic of autocatalysis^34−42^ and thus confirmed previous results that the sigmoidal kinetics found for polyether cascade cyclizations on π-acidic surfaces^14−16^ originate from autocatalysis and do not involve unrelated phenomena. The expected dependence and independence were found for the initial rate *k*_cat_ (Figure 5B, blue filled circles) and the autocatalysis *k*_ac_ (Figure 5B, green empty circles), respectively, on the concentration of **23** up to ∼1 M.

The above solvent screening revealed that the cyclization of **20** with catalyst **32** accelerates dramatically in hexafluorobenzene **1** (Figure 3A). At 10 °C, the addition of product **23** at the beginning of the reaction decelerated rather than accelerated cyclization (Figure 5C). The underlying decreases of both *k*_cat_ and *k*_ac_ (Figure 5D) suggested that the hexafluorobenzene solvent and product **23** compete for the site on the π surface and that the hexafluorobenzene solvent is the more potent co-catalyst. As with NDI dimers in **TS-3**, hexafluorobenzene was likely to activate the NDI catalyst **32** by providing extended intermolecular polarizability into face-to-face stacks as outlined in **TS-5** (Figure 5Ja). In π-stacked foldamers, such induced anion-(π)_*n*_–π rather than intrinsic anion−π interactions have afforded maximal catalytic activity.^67^ Possibly, π-stacked hexafluorobenzene could also activate the nucleophile through hydrogen bonding (Figure 5Jb). Product addition could then destroy the hexafluorobenzene stacks in **TS-5** to bind to the catalyst surface, which reduces the hexafluorobenzene-mediated extra activity (Figure 5Jc, 5C,D).

The unusual reverse impact or “anti-autocatalysis” of product addition in hexafluorobenzene at the beginning was reproduced with product mimic **36** (Figure 5E). Added as a co-catalyst in place of the product to substrate **20** and catalyst **32** in **1** at 10 °C, **36** caused the decrease of *k*_cat_ without much affecting *k*_ac_ (Figure 5F), indicating its inferiority to product **23** as a co-catalyst.

### Asymmetric Anion−π Autocatalysis

To explore the enantioselective autocatalysis with chiral co-catalysts, the best performing conditions were selected, that is cyclization of racemic **20** with NDI catalyst **32** in CD_2_Cl_2_ or hexafluorobenzene. Different concentrations of enantioenriched product **23a** were added at the beginning, and the consumption of the two substrate enantiomers was monitored throughout the reaction by chiral GC. In most measurements, insignificant differences were found between the consumption of the two substrate enantiomers. Hints toward kinetic resolution could occasionally be observed toward the end of the reaction in hexafluorobenzene. For instance, depending on the concentrations of enantioenriched product **23a**, enantiomeric ratios of the remaining substrate increased from nearly negligible er = 53:47 with 0.75 equiv **23a** at 94% conversion (Figure 5G) to er = 67:33 with 3.0 equiv of **23a** at 80% conversion after 7 days (Figure 5H). With the chiral product mimic **36** as a co-catalyst, results were similar. For example, with 1.5 equiv, er = 56:44 was measured at 86% conversion after 12 days (Figure 5I). Overall, the achieved enantioselectivity was only minor in these studies toward kinetic resolution by anion−π autocatalysis. Most importantly, the observations were not consistently reproducible, and the appearance of enantioselectivity was somehow erratic. This puzzling behavior at increased peripheral crowding suggested that the active structure of the autocatalytic system might be more complex than assumed in **TS-1** (Figure 2).

### The Importance of Water

The overall poor responsiveness of anion−π autocatalysis to peripheral crowding called for an explanation. Decreased *k*_cat_ was as expected from **TS-1** and could also be explained with unrelated effects. However, the persistence of autocatalysis with increasing crowding was inconsistent with **TS-1**. Moreover, at preserved autocatalysis, **TS-1** would suggest an increase in enantioselectivity, but kinetic resolution remained negligible and erratic.

**Figure 6:**
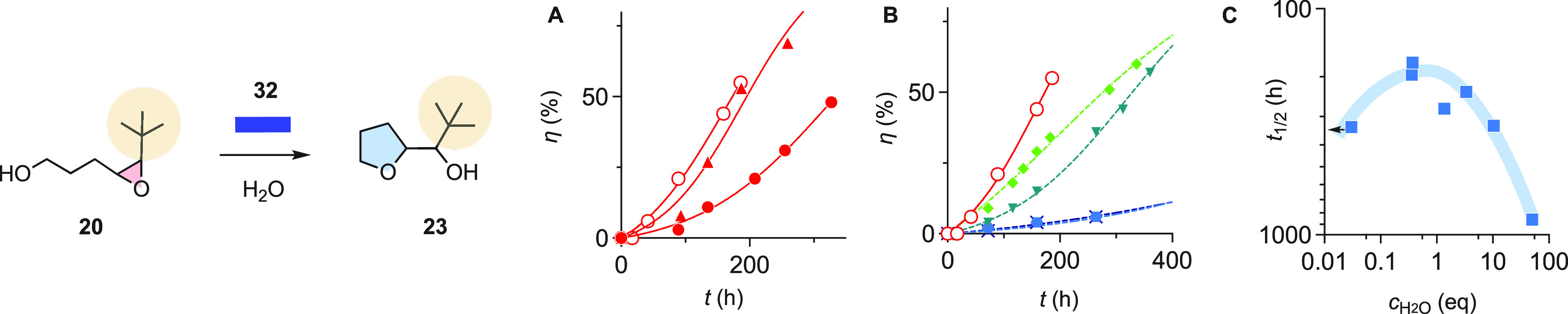
Conversion η of substrate **20** (500 mM) in the presence of catalyst **32** (25 mM) in (A) “dry” CH_2_Cl_2_ (red triangles), further dried CH_2_Cl_2_ (red filled circles), and “wet” CD_2_Cl_2_ (red empty circles) with (B) additional 1, 10, 50, and 100 equiv of water (compared to substrate, green to blue). (C) Dependence of *t*_1/2_ on the concentration of H_2_O in dichloromethane. H_2_O concentrations in dichloromethane were estimated from ^1^H NMR spectra.

The insertion of water between the substrate and product in **TS-1** could conceivably explain these observations. It has been previously observed with several substrates that in specially dried CD_2_Cl_2_, anion−π autocatalysis could decelerate, sometimes even vanish. However, these observations were long considered less relevant and not further followed up. For the cyclization of the peripherally crowded epoxide **20** with catalyst **32**, the comparison of rates obtained in commercially available “dry” CH_2_Cl_2_ (*k*_cat_ = 0.9 ± 0.3 × 10^–5^ M^–1^ s^–1^, *k*_ac_ = 3.2 ± 0.5 × 10^–4^ M^–2^ s^–1^) with those in further dried CH_2_Cl_2_ using molecular sieves (*k*_cat_ = 0.44 ± 0.06 × 10^–5^ M^–1^ s^–1^, *k*_ac_ = 2.1 ± 0.2 × 10^–4^ M^–2^ s^–1^, Figures 6A, C) confirmed that water removal can significantly decelerate anion−π autocatalysis. Prior observations support that it is not unlikely that vigorous water exclusion throughout the entire process would completely suppress anion−π autocatalysis. The addition of more water to not dried CD_2_Cl_2_ decreased the catalytic activity (Figures 6B,C). At ≥ 50 equiv, upon appearance of water droplets, catalytic activities almost disappeared. The limitation of top activity around 0.5–3 equiv of water was important to clarify that this is not another example for catalysis in water^50−54^ but for the less common function of monomeric or oligomeric water as co-catalyst in, by definition, supramolecular catalysis.^5,58−61^ At concentrations below visible phase separation and above the solubility limit (83 mM at 20 °C in CH_2_Cl_2_),^68^ water in CD_2_Cl_2_ probably exists as small hydrogen-bonded clusters stabilized by the facially amphiphilic substrates and products in reversed micelle-like structures. Without anion−π catalysts, cyclizations do not occur in these substrate–product–water oligomers. However, it is quite likely that these mini-micelle-like oligomers bind preassembled and preorganized to the catalytic π surface to undergo cyclization, thereby lowering the entropy penalty expected for the assembly of a five-component transition state like **TS-2**. In general and also below the solubility limit, the repulsion between water and aprotic solvents will contribute favorably to the formation of **TS-2**, which hides the water molecules from the hydrophobic environment.

While the general overall trends were well reproducible, individual kinetics data could vary erratically for often unknown reasons. In this situation, we considered the determination of kinetic isotope effects as not meaningful. High precision control of water concentrations under glovebox conditions was beyond the scope of this study on a qualitative understanding of origin of anion−π autocatalysis rather than the application of the lessons learned to reach full control. While future studies might clarify whether or not such quantitative control is intrinsically possible, the complexity of the active supramolecular system outlined in this study implies that the required efforts will be high.

### Molecular Modeling

**Figure 7:**
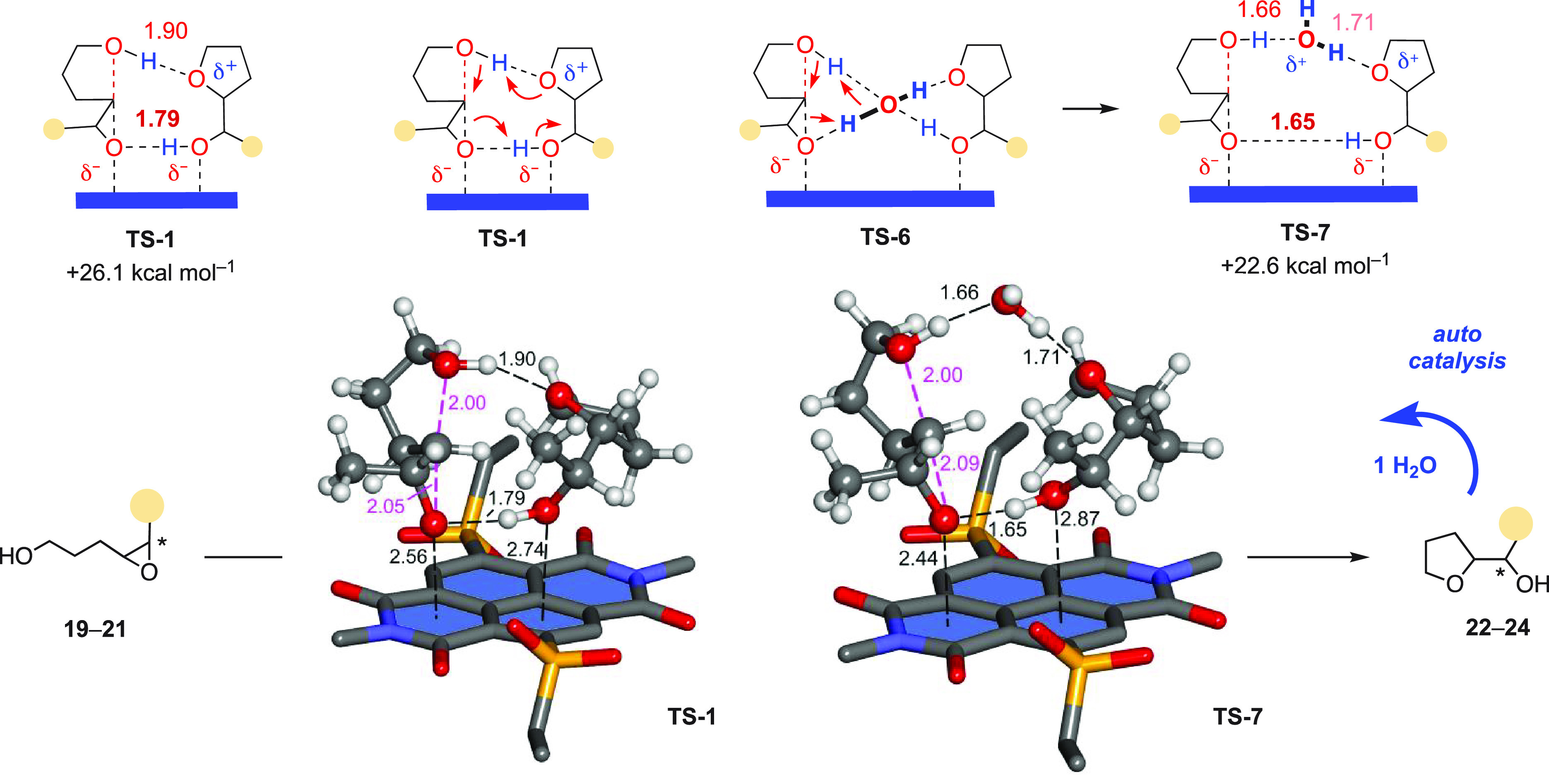
Energy-minimized structure of the original transition state **TS-1** for anion−π autocatalysis showing the transition state accessed from substrate **19** (*S,R* enantiomer) and product **22** (*R,R*) on the surface of anion−π catalyst **33′** (Me instead of LH) and **TS-7** obtained by minimizing **TS-6** with the water included between substrate and product moving from the closed proton shuttle in **TS-6** to the surface of **TS-7**, with pertinent distances and step barriers.

Taken together, these results on peripheral crowding, stereoselectivity, and the importance of water provided compelling support that the original **TS-1** is incorrect (Figures 2 and 7). In **TS-1**, the product binds to the catalytic π surface with a lone-pair−π interaction and stabilizes the transition state with two hydrogen bonds (Figure 7). The product and transition state can be visualized as reminiscent of two dancers on stage holding hands. The hydrogen-bond acceptor activates the nucleophile. The hydrogen-bond donor contributes to the activation of the intramolecular leaving group. The anion−π interaction emerges from this epoxide opening. The negative charge of the resulting alcoholate intermediate is then delocalized to the secondary alcohol in product. This secondary alcohol is also bound to the π surface and therefore develops another partial anion−π interaction upon charge delocalization along the linear hydrogen-bonding relay. The length of the hydrogen bond connecting the “anion−π double bond” is comparably long, suggesting that the charge delocalization is not perfect and the structure is slightly strained (1.79 Å). This strain in **TS-1** is further supported by the long hydrogen bond activating the nucleophile (1.90 Å)

The most appealing way to integrate one molecule of water into **TS-1** would transmit dual substrate activation by the product in a closed proton shuttle that bypasses charge separation in the transition state by transferring the proton from nucleophile to leaving group in a concerted manner. In this **TS-6**, the product would serve only as a scaffold to correctly position the water co-catalyst (Figure 7). The same would be true for the π-acidic surface, which does not interact directly with the water.

Molecular models of **TS-6** were computed using the B3LYP functional^69−72^ adding Grimme’s D3 dispersion correction^73^ and combined with the 6-31+G* basis set.^74^ Solvent effects (CH_2_Cl_2_) were considered using a polarized continuum model (PCM).^75^ During minimization of the hypothetical **TS-6**, the water molecule moved from the central cyclic relay to the periphery of the complex. In the resulting **TS-7**, the water activates only the nucleophile through a linear relay to the hydrogen-bond donor of the product. The water has, however, lost contact with the epoxide, and does thus not contribute to the activation of the leaving group. This rate-limiting leaving group activation is achieved by the hydrogen-bond coupled anion−π double bond as in **TS-1**. The length of the hydrogen bond connecting the anion−π double bond in **TS-7** (1.65 Å) is shorter than in **TS-1** (1.79 Å), suggesting, possibly, that the charge delocalization is better and the structure less strained. In the accepting hydrogen-bonded chain, the first hydrogen bond shortened from 1.90 Å in **TS-1** to 1.66 Å in **TS-7**. The second hydrogen bond in the accepting chain is almost as short (1.71 Å), supporting that the inclusion of one molecule of water in **TS-7** improves not only the activation of the leaving group (−0.14 Å) but also the activation of the nucleophile (−0.24 Å). These differences in hydrogen bonding were reflected in the computed step barriers, which decreased from *E*_a_ = +26.1 kcal mol^–1^ for the original working model **TS-1** without to *E*_a_ = +22.6 kcal mol^–1^ for **TS-7** with one water molecule.

**Figure 8:**
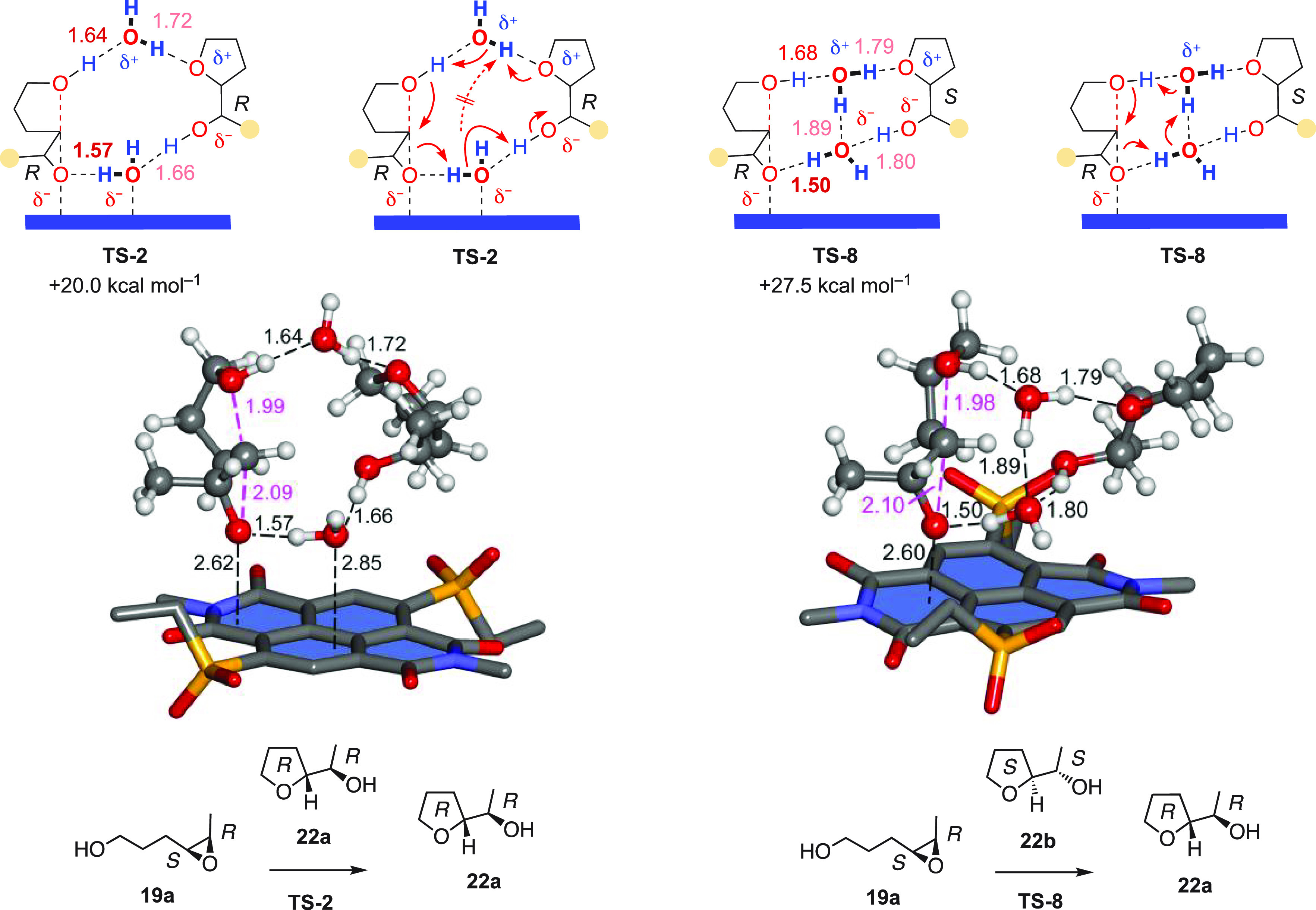
Energy-minimized structure of the new transition state **TS-2** for anion−π autocatalysis showing two water molecules included between substrate **19** (*S,R* enantiomer) and product **22a** (*R,R*) on the surface of anion−π catalyst **33′** (Me instead of LH) to transmit dual activation, delocalize charges in decoupled hydrogen-bonded chains, and stabilize negative charge density with an anion−π double bond, compared to **TS-8** with product **22b** (*S,S*) to transmit dual activation in a closed proton shuttle without explicit stabilization of negative charge density and an anion−π single bond only, both with pertinent distances and step barriers.

The misbalanced **TS-7** could insert another water molecule into the second hydrogen bond between product and transition state. In the resulting **TS-2**, this second water molecule activates the epoxide opening as a hydrogen-bond donor and connects this activation to the hydrogen-bond donor in the product with a linear relay (Figure 8). The second water molecule has no contact with the first water molecule activating the nucleophile. This excluded the cyclic proton shuttle without transient charge separation considered in the nonexistent **TS-6**. The result are two antiparallel hydrogen-bonded chains that enable product and substrate to communicate over a long distance without direct physical contact and launch autocatalysis by remote control. In the charge-separated intermediate emerging in **TS-2**, the interaction of the second water with the aromatic surface is thus also a partial anion−π interaction. The length of the hydrogen bond connecting the anion−π double bond in **TS-2** (1.57 Å) is very short, shorter than in **TS-7** (1.65 Å, + 0.08 Å) with one water molecule and much shorter than in **TS-1** (1.79 Å, + 0.22 Å) without a water molecule, implying that charge delocalization is best and the structure most relaxed. The hydrogen-bond accepting chain in **TS-2** (1.64, 1.72 Å) is almost unchanged for **TS-7** (1.66, 1.71 Å). The short length of both but particularly the first hydrogen bond supported that already in **TS-7**, the accepting chain is nearly ideal to activate the nucleophile and delocalize the emerging positive charge density.

These conclusions were supported by the smallest computed step barrier, i.e., *E*_a_ = +20.0 kcal mol^–1^ for **TS-2** compared to the slightly less favored **TS-7** with *E*_a_ = +22.6 kcal mol^–1^ and the more disfavored **TS-1** with *E*_a_ = +26.1 kcal mol^–1^ (Figures S16–S21, Table S11). The consistent trends obtained for activation energies compared to hydrogen-bond length were remarkable considering the complexity of the transition-state structures. Their multicomponent architectures are part of a conformational landscape that is far too complex to be explored comprehensively at a sufficient level of theory. Moreover, five-component transition states appear unfavorable for a rather high entropy penalty. However, for anion−π autocatalysis, preassembly and preorganization of substrate–product–water complexes as mini-micelles in CD_2_Cl_2_ are expected to minimize the entropy penalty for the assembly of the active **TS-2** from five components (and raise the enthalpy barrier for **TS-7** and **TS-1** by ejecting water into hydrophobic environments). More precise theoretical predictions of activation energies are hindered by the complexity of different multicomponent intermediates preceding the different transition states, presumably including water solubilizing reverse micelle effects that are difficult to compute at high level.

Long-distance autocatalysis without physical contact between substrate and product was in agreement with negligible stereoselectivity (Figure 5). Computational simulations of the (*S*,*R*)-substrate **19a** subjected to autocatalysis with the enantiomer of its (*R*,*R*)-product **22a**, that is (*S*,*S*)-**22b**, gave **TS-8**. This **TS-8** is almost complementary to **TS-2** with the (*S*,*R*)-substrate **19a** subjected to autocatalysis with its (*R*,*R*)-product **22a**. The hydrogen-bonded chains form a closed circle to shuttle protons in a concerted manner without separation of charges in the transition state and pertinent reactive intermediates. Complementary to **TS-2**, this concerted proton shuttle mechanism does not involve significant anion−π interactions in the transition state. It was thus meaningful that only the epoxide oxygen of the substrate keeps contact with the π surface to assist in activation of the leaving group, whereas the water oxygen uses both lone pairs to close the hydrogen-bonded chain and accept the hydrogen bond from the product. The primary hydrogen bonds from the two waters activating nucleophile and leaving group in “racemic”^76^**TS-8** (1.50, 1.68 Å) were as good as in the “enantiopure” **TS-2** (1.57, 1.64 Å), maybe even slightly better. Continuing charge delocalization was, however, less convincing. The very long hydrogen bond (1.89 Å) closing the cyclic proton shuttle in **TS-8** confirmed the general trend that water–water hydrogen bonding is not favored in the autocatalytic transition state, while continuing charge delocalization to the product (1.80, 1.79 Å) was necessarily weaker in **TS-8** compared to **TS-2** with decoupled antiparallel hydrogen-bonded chains (1.66, 1.72 Å). These observations were perfectly reflected in a high step barrier of *E*_a_ = +27.5 kcal mol^–1^ of proton shuttle **TS-8** for “racemic”^76^ anion−π autocatalysis, clearly above the “anion−π” **TS-2** for asymmetric anion−π autocatalysis with *E*_a_ = +20.0 kcal mol^–1^.

Computational simulations thus confirmed experimental results that chiral resolution with anion−π autocatalysis should in principle be possible (Figure 5). The overall small energy differences in computational models as well as their complexity including two water molecules reflect well the difficulties to find stable systems that would be needed to achieve significant stereoselectivity. Moreover, it is understood that computed energies should not be overrated, considering the impact of other contributions, including desolvation and other entropy losses in complex multicomponent architectures. However, the striking difference in energy as well as mechanism between the diastereomeric **TS-2** and **TS-8** supports that long-distance autocatalysis through hydrogen-bonded chains without physical contact between substrate and product is not *a priori* incompatible with asymmetric anion−π autocatalysis.

The final **TS-2** is a supramolecular architecture of beautiful complexity. It contains two short, decoupled hydrogen-bonded chains of opposite directionality. They integrate two molecules of water that transmit dual activation of the substrate by the product co-catalyst in a stereoselective manner. Similar hydrogen-bonded chains have been identified at work in supramolecular catalysis^5,58−61^ as well as in other functional systems, particularly as proton wires in transmembrane transport.^77^ Besides the activation of nucleophile and leaving group by the product co-catalysts, the short, bidirectional hydrogen-bonded chains in **TS-2** serve to establish anion−π double bonds with connecting hydrogen bonds as short as possible for transition-state stabilization, i.e., anion−π catalysis (Figure 8). The length of the hydrogen bond building the anion−π double bond correlated particularly well with the step barrier of the respective transition states with two (**TS-2**, 1.57 Å, +20.0 kcal mol^–1^), one (**TS-7**, 1.65 Å, +22.6 kcal mol^–1^), and zero molecules of water (two (**TS-1**, 1.79 Å, +26.1 kcal mol^–1^). These maximized anion−π double bonds in **TS-2** were of particular interest because they make use of the charge-delocalized nature that has been described as the distinguishing characteristic of anion−π interactions.^8^ This was intriguing because the integration of distinguishing characteristics of unorthodox interactions into catalytic systems is generally expected to provide access to new properties. The anion−π double bond with an ultrashort hydrogen-bond connector in the new theoretical model for anion−π autocatalysis, i.e., **TS-2**, thus provides a wonderful illustration that these high expectations can be met.

## Conclusions

The general objective of this study was to elucidate the mechanism of the autocatalytic ether cyclizations on π-acidic aromatic surfaces. This fascinating process is appreciated as an example for new properties that can emerge with the integration of unorthodox interactions into functional systems. However, how exactly autocatalysis takes place on π-acidic surfaces has remained a mystery. The present study combines peripheral crowding, stereoselectivity, the dependence on water, and computational simulations to explore the origin of anion−π autocatalysis. Besides intriguing extras like DMSO and hexafluorobenzene as co-catalysts, the key results support an active structure with two molecules of water included between the transition state and product co-catalyst on the catalytic π-acidic surface. They create two independent antiparallel hydrogen-bonded chains that transmit nucleophile and leaving group activation by the product and stabilize the emerging charges by a charge delocalization that extends into formal anion−π double bonds. The resulting remote control of autocatalysis by long distance communication without direct physical contact is consistent with the identified characteristics, highlights unique advantages from the delocalized nature of anion−π interactions, satisfies scientific curiosity and drives the structural complexity of anion−π catalysis to an unprecedented level of sophistication.

## Supporting Information Available

Detailed experimental procedures, materials and methods, compound synthesis and characterization, reaction kinetics, kinetics analysis, dependence on solvents, catalysts, co-catalysts, enantioenriched co-catalysts, catalyst concentrations, and temperatures, theoretical methods, Cartesian coordinates, frequency calculations, Gibbs free energies, activation energies, and original NMR spectra (PDF)
