# Supplementary material for: The Origin of Anion−π Autocatalysis
Source: JACS Au. 2023 Mar 17;3(4):1039–51. doi: 10.1021/jacsau.2c00656 (PMC10131205; doi:10.1021/jacsau.2c00656)
Supplement: Supplementary file 1 — au2c00656_si_001.pdf [file au2c00656_si_001.pdf]

Supporting Information

The Origin of Anion-π Catalysis

M. Ángeles Gutiérrez López,[†,‡] Mei-Ling Tan, [†,‡] Antonio Frontera,[§] and Stefan Matile*[,†,‡]

[†]Department of Organic Chemistry, University of Geneva, CH-1211 Geneva, Switzerland

[‡]National Centre of Competence in Research (NCCR) Molecular Systems Engineering (MSE), CH-4002 Basel, Switzerland

[§]Departament de Química, Universitat de les Illes Balears, SP-07122 Palma de Mallorca, Spain

E-mail: stefan.matile@unige.ch

Table of Contents

1. Materials and Methods S3

2. Synthesis S4

   2.1. Synthesis of Crowded Substrates S4

   2.2. Synthesis of Crowded Products S6

   2.3. Synthesis of Enantioenriched Crowded Products S7

   2.4. Synthesis of Overcrowded Substrates S9

   2.5. Synthesis of Overcrowded Products S12

   2.6. Synthesis of Anion-π Catalysts S13

3. Catalysis S15

   3.1. Reaction Kinetics Measurements by NMR Spectroscopy S15

   3.2. Reaction Kinetics Analysis S15

   3.3. Dependence on Solvents S16

   3.4. Dependence on Catalyst Concentration S17

   3.5. Dependence on Temperature S19

   3.6. Dependence on Anion-π Catalysts S19

   3.7. Dependence on Co-Catalysts S20

   3.8. Dependence on Enantioenriched Co-Catalysts S32

   3.9. Dependence on Water S37

4. Theoretical Methods S37

   4.1. Frequency Calculations and Six First Frequencies Values S38

   4.2. Gibbs Free Energies and Activation Energy Barriers S42

   4.3. Cartesian Coordinates, Gibbs Free Energies and ZPE Values in Hartree S42

5. NMR Spectra S53

6. Supporting References S68

1. Materials and Methods

As in ref. S1, Supporting Information. Reagents for synthesis were purchased from Merck, Apollo Scientific, Broadpharm and Acros. Column chromatography was carried out on silica gel (SiliaFlash® P60, SILICYCLE, 230–400 mesh). Flash column chromatography was performed on a Biotage Isolera^{TM} system. Analytical (TLC) and preparative thin layer chromatography (PTLC) were performed on silica gel 60 F254 (Merck) and silica gel (SiliCycle, 1000 μm), respectively. μW reactions were performed in a Discover SP-Microwave Synthesizer (CEM). Temperature of the kinetic reactions was maintained using a minichiller 300 OLÉ for RT (25 °C) and PSL 2500 temperature controller for 10 °C. Chiral HPLC were performed on a LC-4000 system from JASCO. Melting points (Mp) were measured on a Melting Point M-565 (BUCHI). Chiral Gas chromatography (GC) was performed on Agilent 6850 Series gas chromatographs equipped with a split-mode capillary injection system and flame ionization detectors using chiral stationary Hydrodex Gamma DiMOM column (50 m x 0.25 mm ID). Separation parameters: 60 °C, 1 °C/min, until 170 °C, then hold at 170 °C for 20 min (Speed: 60 cm/s H_2, injector temperature: 170 °C). Alfa-D values were measured on a Polarimeter P-1030 (Jasco). IR spectra were recorded on a Perkin Elmer Spectrum One FT-IR spectrometer (ATR, Golden Gate) and are reported as wavenumbers ν in cm^{-1} with band intensities indicated as s (strong), m (medium), w (weak). ^{1}H, ^{19}F, and ^{13}C NMR were recorded (as indicated) either on a Bruker 300 MHz, 400 MHz, or 500 MHz spectrometer and are reported as chemical shifts (δ) in ppm relative to TMS (δ = 0). Spin multiplicities are reported as a singlet (s), doublet (d), triplet (t) and quartet (q), with coupling constants (*J*) given in Hz, or multiplet (m). Broad peaks are marked as br. Accurate mass determinations using ESI (HR ESI-MS) were performed on Xevo G2-S Tof (Waters).

Abbreviations. *m*-CPBA: *meta*-Chloroperoxybenzoic acid; DMP: Dess-Martin periodinane; LiHDMS: Lithium bis(trimethylsilyl)amide; NDI: Naphthalenediimide; RT: Room temperature;

TBAF: Tetra-*n*-butylammonium fluoride; TBDPSCl: *tert*-Butyl(chloro)diphenylsilane; µW: Microwave.

2. Synthesis

Compound 19 was prepared following previously reported procedures described in ref. S2.

Compound 22 was prepared following previously reported procedures described in ref. S3.

Compound 32 was prepared following previously reported procedures described in ref. S4.

Compound 33 was prepared following previously reported procedures described in ref. S5.

Compound 34 was prepared following previously reported procedures described in ref. S6.

2.1. Synthesis of Crowded Substrates

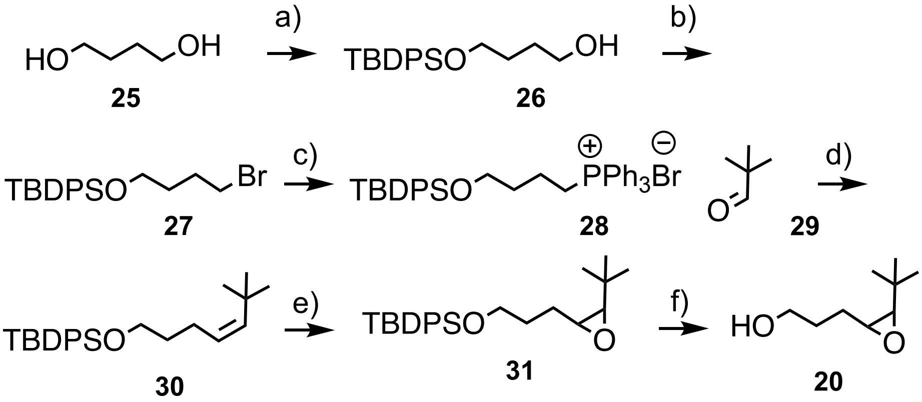

Scheme S1. a) 1. NaH, THF, 0 ºC, 30 min; 2. TBDPSCl, THF, 0 °C, 2 h, quant; b) PPh_3, CBr_4, CH_2Cl_2, 0 °C to RT, 2 h, 67%; c) PPh_3, toluene, 150 °C, 15 h, 65%; d) 1. LiHDMS, THF, -78 °C, 30 min; 2. **29**, -78 °C to RT, 15 h, 79%; e) *m*-CPBA, CH_2Cl_2, 0 °C to RT, 15 h, 91%; f) TBAF, THF, 0 °C to RT, 2 h, 89%.

Compound 26 was prepared following previously reported procedures described in ref. S7.

Compound 27 was prepared following previously reported procedures described in ref. S8.

Compound 28. PPh_3 (4.30 g, 16.2 mmol) was dissolved in toluene (40 mL) in an oven-dried Schlenk flask. The flask was evacuated and back-filled with nitrogen for 3 times. Then, **27** (neat, 6.35 g, 16.2 mmol) was added via syringe and the mixture was stirred for 15 h at 150 °C in the pressure-

tight flask. The mixture was cooled down and diluted with Et_2O (30 mL). The resulting precipitate was filtered, washed with Et_2O (2 x 20 mL), and dried under vacuum to give **28** (10.6 g, 65%) as a colorless solid. R_f (*n*-pentane/EtOAc 5:1): 0.77; Mp: 145 – 146 °C; IR (neat): 2854 (w), 1435 (m), 1110 (s), 995 (w), 840 (w), 746 (m), 701 (s), 689 (s); ^{1}H NMR (400 MHz, $CDCl_3$): 7.84 – 7.75 (m, 9H), 7.71 – 7.63 (m, 6H), 7.54 – 7.50 (m, 4H), 7.42 – 7.27 (m, 6H), 3.92 – 3.81 (m, 2H), 3.72 (t, ^3J = 5.4 Hz, 2H), 1.99 – 1.93 (m, 2H), 1.83 – 1.73 (m, 2H), 0.93 (s, 9H); ^{13}C NMR (101 MHz, $CDCl_3$): 135.4 (CH), 135.0 (d, $^4J_{C\text{-}P}$ = 2.9 Hz, CH), 133.7 (d, $^2J_{C\text{-}P}$ = 9.9 Hz, CH), 133.6 (C), 130.5 (d, $^3J_{C\text{-}P}$ = 11.2 Hz, CH), 129.7 (CH), 127.7 (CH), 118.3 (d, $^1J_{C\text{-}P}$ = 85.8 Hz, C), 62.7 (CH_2), 32.7 (d, $^3J_{C\text{-}P}$ = 16.1 Hz, CH_2), 26.9 (CH_3), 22.4 (d, $^1J_{C\text{-}P}$ = 50.3 Hz, CH_2), 19.4 (d, $^2J_{C\text{-}P}$ = 4.0 Hz, CH_2), 19.2 (C).

Compound 30. Compound **28** (3.65 g, 5.60 mmol) was dissolved in dry THF (40 mL) in an oven-dried Schlenk flask. The flask was evacuated and back-filled with nitrogen for 3 times. Then, the solution was cooled to –20 °C and LiHDMS solution in THF (1.0 M, 11.6 mL, 11.6 mmol) was added via syringe dropwise. The mixture was stirred at the same temperature for 30 min followed by the addition of **29** (0.53 mL, 4.65 mmol) dropwise. The mixture was stirred for 15 h at RT. The reaction mixture was quenched with saturated aqueous NH_4Cl (20 mL) and the organic phase was extracted with EtOAc (3 x 30 mL). The combined organic phases were dried over Na_2SO_4 and concentrated under vacuum. Further purification by flash column chromatography (pentane/EtOAc 49:1) gave **30** (1.40 g, 79%) as a colorless oil. R_f (*n*-pentane/EtOAc 5:1): 0.84; IR (neat): 2956 (w), 2932 (w), 2859 (w), 1473 (w), 1428 (w), 1362 (w), 1106 (s), 823 (m), 699 (s); ^{1}H NMR (500 MHz, $CDCl_3$): 7.72 – 7.64 (m, 4H), 7.49 – 7.34 (m, 6H), 5.32 (dt, ^3J = 12.0, ^4J = 1.7 Hz, 1H), 5.14 (dt, ^3J = 12.0, 7.5 Hz, 1H), 3.69 (t, ^3J = 6.4 Hz, 2H), 2.27 (qd, ^3J = 7.5, ^4J = 1.7 Hz, 2H), 1.66 – 1.58 (m, 2H), 1.10 (s, 9H), 1.05 (s, 9H); ^{13}C NMR (126 MHz, $CDCl_3$): 140.1 (CH), 135.6 (CH), 134.1 (C), 129.5 (CH), 128.4 (CH), 127.6 (CH), 63.6 (CH_2), 33.3 (CH_2), 33.2 (C), 31.1 (CH_3), 26.9 (CH_3), 24.8 (CH_2), 19.2 (C).

Compound 31. Compound **30** (0.34 g, 0.89 mmol) was dissolved in dry CH_2Cl_2 (30 mL) and *m*-CPBA (0.26 g, 1.07 mmol) was added portion wise at 0 °C. After addition, the mixture was stirred for 15 h at RT. The reaction mixture was concentrated under vacuum and purified by flash column chromatography (*n*-pentane/Et_2O 4:1) to give **31** (0.32 g, 91%) as a colorless oil. R_f (pentane/EtOAc 1:1): 0.63; IR (neat): 3073 (w), 2957 (w), 2859 (w), 1473 (w), 1428 (w), 1363 (w), 1106 (s), 822 (m), 700 (s); ^1H NMR (500 MHz, $CDCl_3$): 7.71 – 7.62 (m, 4H), 7.47 – 7.33 (m, 6H), 3.79 – 3.66 (m, 2H), 2.85 (td, ^3J = 6.1, 4.4 Hz, 1H), 2.64 (d, ^3J = 4.4 Hz, 1H), 1.86 – 1.63 (m, 4H), 1.05 (s, 9H), 1.02 (s, 9H); ^{13}C NMR (126 MHz, $CDCl_3$): 135.7 (CH), 134.0 (C), 129.7 (CH), 127.7 (CH), 65.6 (CH), 63.6 (CH_2), 59.2 (CH), 31.7 (C), 30.7 (CH_2), 28.0 (CH_3), 27.0 (CH_3), 25.2 (CH_2), 19.3 (C).

Compound 20. Compound **31** (0.10 g, 0.26 mmol) was dissolved in dry THF (3 mL) and a solution of TBAF in THF (1.0 M, 0.40 mL, 0.40 mmol) was added dropwise at 0 °C. After addition, the mixture was stirred for 2 h at RT. Then, the solvent was removed under vacuum and the crude was purified by flash column chromatography (*n*-pentane/Et_2O 2:1 to Et_2O) to achieve **20** (0.04 g, 89%) as a colorless oil. R_f (pentane/Et_2O 2:1): 0.08; IR (neat): 3380 (s), 2958 (s), 2870 (s), 1484 (m), 1451 (m), 1364 (m), 1364 (s), 1203 (w), 1060 (s), 921 (s), 833 (m); ^1H NMR (500 MHz, CD_2Cl_2): 3.67 (t, ^3J = 5.3 Hz, 2H), 2.86 – 2.82 (m, 1H), 2.65 (d, ^3J = 4.4 Hz, 1H), 1.86 – 1.65 (m, 4H), 1.00 (s, 9H); ^{13}C NMR (126 MHz, CD_2Cl_2): 65.9 (CH), 62.7 (CH_2), 59.3 (CH), 31.4 (C), 30.8 (CH_2), 28.0 (CH_3), 25.3 (CH_2); HRMS (ESI): calcd. for $C_9H_{18}O_2$ ($[M+Na]^+$): 181.1200, found: 181.1203.

2.2. Synthesis of Crowded Products

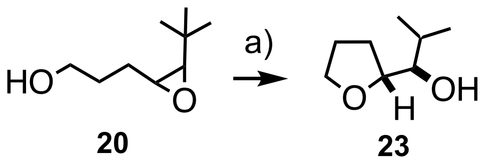

Scheme S2. a) $SbCl_3$, CD_2Cl_2, RT, 2 h, 85%.

Compound 23. Compound **20** (0.10 g, 0.63 mmol) was dissolved in CD_2Cl_2 (0.10 mL) and $SbCl_3$ (0.01 g, 0.63 mmol) was added. After addition, the mixture was stirred for 2 h at RT. Then, the crude mixture was diluted with CD_2Cl_2 (1.0 mL) and washed with saturated aqueous NaOH solution (2 x 1.0 mL) and H_2O (1 x 1.0 mL). The organic phase was dried over Na_2SO_4 and concentrated under vacuum to give **23** (0.09 g, 85%) as a colorless oil. R_f (pentane/Et_2O 1:1): 0.74; IR (neat): 3495 (w), 2953 (s), 2870 (m), 1479 (w), 1395 (w), 1363 (w), 1099 (m), 1055 (s), 998 (m), 894 (m); ^1H NMR (500 MHz, CD_2Cl_2): 3.93 (ddd, ^3J = 8.1, 6.5, 3.3 Hz, 1H), 3.84 – 3.73 (m, 2H), 3.04 (dd, ^3J = 7.2, 3.3 Hz, 1H), 2.52 (d, ^3J = 7.2 Hz, 1H), 2.00 – 1.69 (m, 4H), 0.91 (s, 9H); ^{13}C NMR (126 MHz, CD_2Cl_2): 80.4 (CH), 78.0 (CH), 68.9 (CH_2), 35.0 (C), 31.1 (CH_2), 26.5 (CH_2), 26.4 (CH_3); HRMS (ESI): calcd. for $C_9H_{18}O_2$ ($[M+Na]^+$): 181.1200, found: 181.1203.

2.3. Synthesis of Enantioenriched Crowded Products

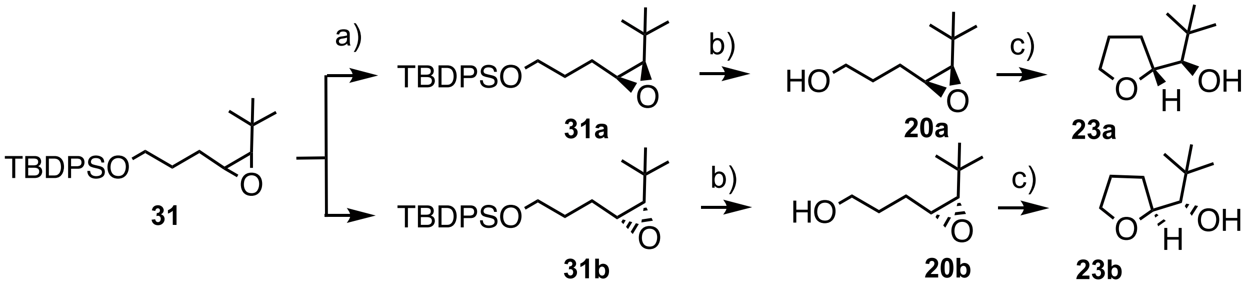

Scheme S3. a) Preparative HPLC: IA-Chiralpak, 2% EtOAc in *n*-hexane, 89% *ee* (**31a**), 96% *ee* (**31b**); b) TBAF, THF, 0 °C to RT, 2 h, 85% (**20a**), 83% (**20b**); c) $SbCl_3$, CD_2Cl_2, RT, 75% (**23a**), 87% (**23b**). While **31a**, **31b**, **20a**, **20b**, **23a** and **23b** are enantioenriched, their absolute configuration is unknown.

Compounds 31a and 31b. Compound **31** (0.50 g, 1.26 mmol) was dissolved in *n*-hexane (4.0 mL) and purified by preparative HPLC (CHIRALPAK® IA (20 mm ø x 250 mm L), 12.8 mL/min, 2% EtOAc in *n*-hexane) to give compounds **31a** (R_t = 6.91 min, 0.20 g, 89% *ee*, 40%) and **31b** (R_t = 7.90 min, 0.19 g, 96% *ee*, 39%) as colorless oils. **31a**: $[\alpha]_D^{20}$ +0.64 (*c* 0.73, CH_2Cl_2). **31b**: $[\alpha]_D^{20}$ -0.54 (*c* 0.75, CH_2Cl_2).

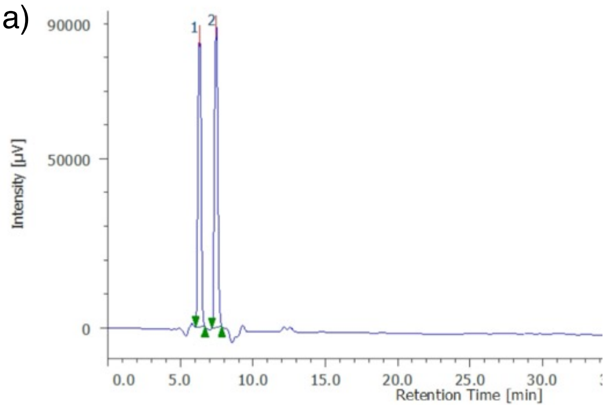

#	Peak Name	CH	Area [µV·sec]	tR [min]	Height [µV]	Area%	Height%
1	Unknown	5	1376877	6.333	85826	48.394	49.056
2	Unknown	5	1468290	7.467	89130	51.606	50.944

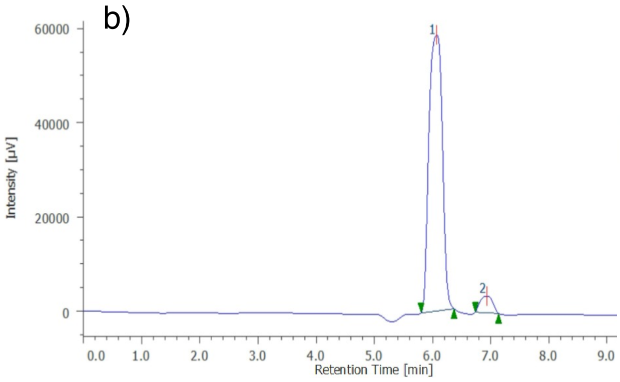

#	Peak Name	CH	Area [µV·sec]	tR [min]	Height [µV]	Area%	Height%
1	Unknown	5	937087	6.067	58580	94.687	94.355
2	Unknown	5	52578	6.933	3505	5.313	5.645

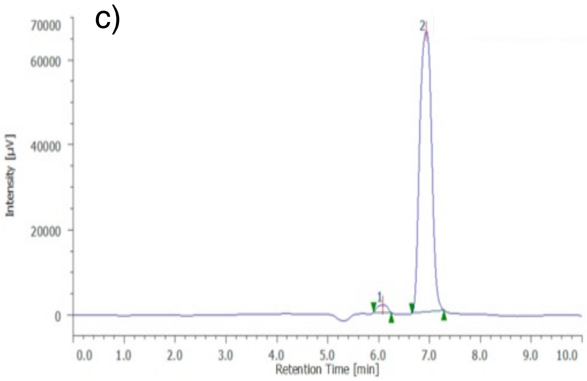

#	Peak Name	CH	Area [µV·sec]	tR [min]	Height [µV]	Area%	Height%
1	Unknown	5	24561	6.083	1702	2.241	2.515
2	Unknown	5	1071215	6.933	65978	97.759	97.485

Figure S1. Analytical HPLC chromatograms of compounds a) **31**, b) **31a**, and c) **31b**. CHIRALPAK® IA (4.6 mm ø x 250 mmL), 0.8 mL/min, 2% EtOAc in *n*-hexane, λ_{abs} = 254 nm.

Compound 20a. Starting from compound **31a** (0.31 g, 0.78 mmol) and following the same procedure as for compound **20**, compound **20a** (0.10 g, 85%) was achieved as a colorless oil. $[\alpha]_D^{20}$ +5.4 (*c* 0.51, CH_2Cl_2).

Compound 20b. Starting from compound **31b** (0.38 g, 0.96 mmol) and following the same procedure as for compound **20,** compound **20b** (0.13 g, 83%) was obtained as a colorless oil. $[\alpha]_D^{20}$ -4.0 (*c* 0.92, CH_2Cl_2).

Compound 23a. Starting from compound **20a** (0.10 g, 0.66 mmol) and following the same procedure as for compound **23**, compound **23a** (0.08 g, 75%) was obtained as a colorless oil.

Compound 23b. Starting from compound **20b** (0.13 g, 0.80 mmol) and following same procedure as for compound **23,** compound **23b** (0.11 g, 87%) was obtained as a colorless oil.

2.4. Synthesis of Overcrowded Substrates

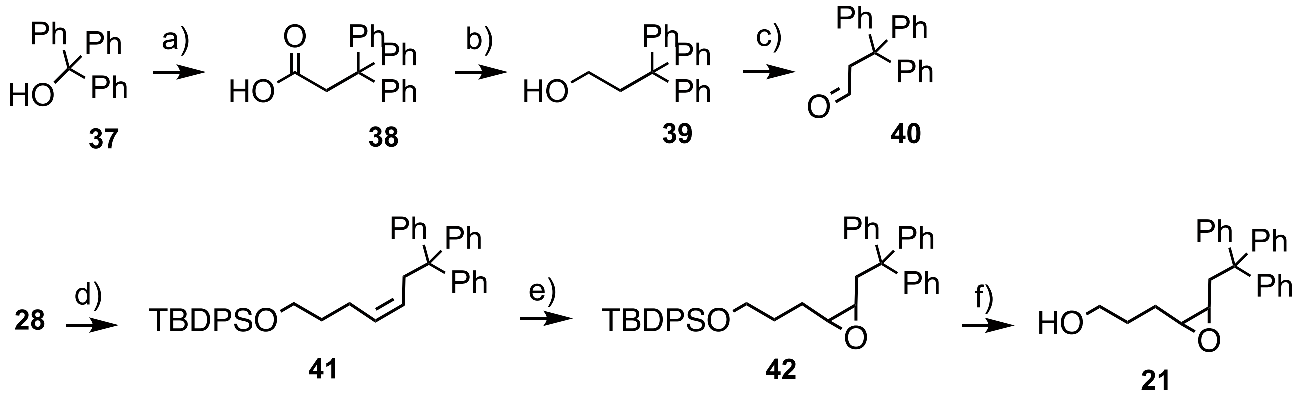

Scheme S4. a) Malonic acid, 150-160 °C, 3 h, 66%; b) $LiAlH_4$, THF, -78 °C to RT, 15 h, 61%; c) DMP, CH_2Cl_2, 0 °C to RT, 15 h, 57%; d) 1. LiHDMS, THF, -78 °C, 30 min; 2. **40**, -78 °C to RT, 15 h, 30%; e) *m*-CPBA, CH_2Cl_2, 0 °C to RT, 15 h, 80%; f) TBAF, THF, 0 °C to RT, 2 h, 95%.

Compound 38 was prepared following previously reported procedures as described in ref. S9.

Compound 39. Compound **38** (11.5 g, 57.8 mmol) was dissolved in dry THF (250 mL) and cooled at -78 °C in an acetone/dry ice bath. $LiAlH_4$ (3.04 g, 76.1 mmol) was added portion-wise and the suspension was stirred for 15 h at RT. Excess of $LiAlH_4$ was quenched with ice, the suspension was filtered, and the filtrate was extracted with EtOAc (2 x 50 mL), washed with brine (1 x 50 mL) and dried over Na_2SO_4. The resulting alcohol **39** (6.21 g, 61%) was used without further purification. R_f (*n*-pentane/EtOAc 8:1): 0.32; IR (neat): 3309 (w), 3055 (w), 3031 (w), 2953 (w), 1594 (m), 1493 (m), 1446 (m), 1392 (m), 1030 (m), 697 (s); ^{1}H NMR (400 MHz, $CDCl_3$): 7.37 – 7.22 (m, 12H), 7.19 – 7.14 (m, 3H), 3.54 – 3.48 (m, 2H), 3.01 – 2.94 (m, 2H), 1.57 (s^{br}, 1H); ^{13}C NMR (101 MHz, $CDCl_3$): 147.0 (C), 129.0 (CH), 128.1 (CH), 126.1 (CH), 60.6 (CH_2), 55.2 (C), 42.9 (CH_2).

Compound 40. Compound **39** (8.00 g, 27.7 mmol) was dissolved in dry CH_2Cl_2 (300 mL) and DMP (14.6 g, 33.3 mmol) was added portion wise at 0 °C. After the addition the mixture was stirred at RT for 6 h. The reaction mixture was quenched with saturated aqueous $NaHCO_3$ (100 mL), and washed with saturated aqueous Na_2SO_3 (100 mL) and brine (100 mL). The organic layer was dried over Na_2SO_4, concentrated under vacuum and purified by flash column chromatography (*n*-pentane/EtOAc 8:1) to give **40** (4.49 g, 57%) as a colorless solid. R_f (*n*-pentane/EtOAc 8:1): 0.15; IR (neat): 1714 (s), 1494 (m), 1444 (m), 1409 (m), 757 (m), 699 (s); ^{1}H NMR (400 MHz, $CDCl_3$): 9.50 (t, ^3J = 2.6 Hz, 1H), 7.35 – 7.20 (m, 9H), 7.18 – 7.14 (m, 6H), 3.63 (d, ^3J = 2.6 Hz, 2H); ^{13}C NMR (126 MHz, $CDCl_3$): 203.2 (CHO), 146.3 (C), 129.1 (CH), 128.5 (CH), 126.8 (CH), 54.6 (C), 54.4 (CH_2).

Compound 41. Compound **28** (5.01 g, 7.66 mmol) was dissolved in dry THF (20 mL) in an oven-dried Schlenk flask. The flask was evacuated and back-filled with nitrogen for 3 times. Then, the solution was cooled to –78 °C and LiHDMS solution in THF (1.0 M, 16.0 mL, 16.0 mmol) was added via syringe dropwise. The mixture was stirred at the same temperature for 30 min followed by the dropwise addition of a solution of **40** (1.83 g, 6.39 mmol) in dry THF (20 mL). The mixture was stirred for 15 h at RT. The reaction mixture was quenched with saturated aqueous NH_4Cl (20 mL) and extracted with EtOAc (3 x 30 mL). The combined organic phases were dried over Na_2SO_4 and concentrated under vacuum. Further purification by flash column chromatography (pentane/EtOAc 49:1) and by preparative HPLC (CHIRALPAK® IA (20 mm ø x 250 mm L), 12.8 mL/min, 2% EtOAc in *n*-hexane) gave **41** (1.10 g, 30%) as a colorless oil. R_f (*n*-pentane/EtOAc 49:1): 0.74; IR (neat): 3056 (w), 2931 (w), 2857 (w), 1494 (w), 1447 (w), 1106 (m), 733 (m), 698 (s), 612 (m); ^{1}H NMR (500 MHz, $CDCl_3$): 7.71 – 7.63 (m, 4H), 7.45 – 7.34 (m, 6H), 7.28 – 7.16 (m, 15H), 5.31 – 5.27 (m, 2H), 3.63 (t, ^3J = 6.3 Hz, 2H), 3.39 (d, ^3J = 4.0 Hz, 2H), 2.07 – 1.99 (m, 2H), 1.56 – 1.49 (m, 2H), 1.06 (s, 9H); ^{13}C NMR (126 MHz, $CDCl_3$): 147.4 (C), 135.7 (CH), 134.2 (C), 130.9 (CH), 129.7

(CH), 129.5 (CH), 127.9 (CH), 127.7 (CH), 127.3 (CH), 126.0 (CH), 63.6 (CH_2), 56.5 (C), 39.0 (CH_2), 32.3 (CH_2), 27.0 (CH_3), 24.1 (CH_2), 19.4 (C).

Compound 42. Compound **41** (1.10 g, 1.89 mmol) was dissolved in dry CH_2Cl_2 (22 mL) and *m*-CPBA (1.03 g, 4.17 mmol) was added portion wise at 0 °C. After addition the mixture was stirred for 15 h at RT. The reaction mixture was concentrated under vacuum and purified by flash column chromatography (*n*-pentane/Et_2O 43:7) to give **42** (1.13 g, 80%) as a colorless oil. R_f (pentane/EtOAc 5:1): 0.77; IR (neat): 2931 (w), 2857 (w), 1494 (w), 1447 (w), 1428 (w), 1106 (m), 732 (m), 698 (s); ^{1}H NMR (400 MHz, CD_2Cl_2): 7.66 – 7.64 (m, 4H), 7.42 – 7.32 (m, 6H), 7.28 – 7.25 (m, 12H), 7.22 – 7.18 (m, 3H), 3.72 – 3.59 (m, 2H), 3.05 (dd, ^2J = 14.8, ^3J = 3.6 Hz, 1H), 2.94 (dt, ^3J = 5.9, 3.6 Hz, 1H), 2.75 – 2.69 (m, 1H), 2.65 (dd, ^2J = 14.8, ^3J = 5.9 Hz, 1H), 1.68 – 1.47 (m, 2H), 1.36 – 1.23 (m, 2H), 1.03 (s, 9H); ^{13}C NMR (126 MHz, $CDCl_3$): 147.0 (C), 135.7 (CH), 134.1 (C), 129.8 (CH), 129.3 (CH), 128.2 (CH), 127.8 (CH), 126.4 (CH), 63.5 (CH_2), 58.2 (CH), 56.0 (C), 55.2 (CH), 39.0 (CH_2), 29.6 (CH_2), 27.0 (CH_3), 24.5 (CH_2), 19.4 (C).

Compound 21. Compound **42** (0.91 g, 1.52 mmol) was dissolved in dry THF (12 mL) and TBAF solution in THF (1.0 M, 2.28 mL, 2.28 mmol) was added dropwise at 0 °C. After addition the mixture was stirred for 2 h at RT. Then, the solvent was removed under vacuum and the crude purified by flash column chromatography (*n*-pentane/Et_2O 2:1 to Et_2O) to achieve **21** (0.52 g, 95%) as a colorless oil. R_f (pentane/Et_2O 1:1): 0.22; IR (neat): 3381 (w), 3056 (w), 2930 (w), 2868 (w), 1493 (m), 1446 (m), 1061 (w), 748 (m), 698 (s); ^{1}H NMR (500 MHz, CD_2Cl_2): 7.32 – 7.26 (m, 12H), 7.25 – 7.19 (m, 3H), 3.61 – 3.53 (m, 2H), 3.03 (dd, ^2J = 14.8, ^3J = 3.9 Hz, 1H), 2.90 (dt, ^3J = 5.9, 3.9 Hz, 1H), 2.71 – 2.65 (m, 2H), 1.63-1.52 (m, 2H), 1.28 – 1.21 (m, 2H); ^{13}C NMR (126 MHz, CD_2Cl_2): 147.3 (C), 129.5 (CH), 128.4 (CH), 126.6 (CH), 62.6 (CH_2), 58.2 (CH), 56.3 (C), 55.4 (CH), 39.0 (CH_2), 30.1 (CH_2), 24.8 (CH_2); HRMS (ESI): calcd. for $C_{25}H_{26}O_2$ ($[M+Na]^+$): 381.1825, found: 381.1813.

2.5. Synthesis of Overcrowded Products

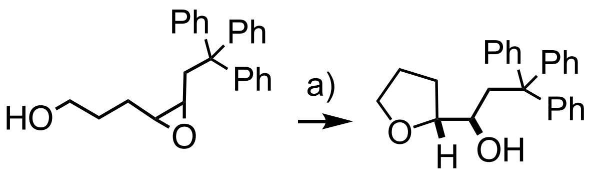

21 **24**

Scheme S5. a) $SbCl_3$, CD_2Cl_2, RT, 2 h, 85%.

Compound 24. Compound **21** (0.05 g, 0.14 mmol) was dissolved in CD_2Cl_2 (0.03 mL) and $SbCl_3$ (0.02 g, 0.07 mmol) was added. After addition the mixture was stirred for 2 h at RT. Then, the crude mixture was diluted with CD_2Cl_2 (1.00 mL) and washed with NaOH saturated solution (2 x 1.00 mL) and H_2O (1 x 1.00 ml). The organic phase was dried over Na_2SO_4 and concentrated under vacuum to give **24** (0.05 g, quantitative) as a colorless oil. R_f (pentane/Et_2O 1:1): 0.51; IR (neat): 3457 (w), 2970 (w), 1493 (m), 1447 (m), 1060 (m), 747 (m), 703 (s); ^{1}H NMR (500 MHz, CD_2Cl_2): 7.41 – 7.37 (m, 6H), 7.30 – 7.25 (m, 6H), 7.20 – 7.16 (m, 3H), 3.80 – 3.65 (m, 2H), 3.50 – 3.44 (m, 1H), 3.36 – 3.32 (m, 1H), 3.00 (ddd, ^2J = 14.8, ^3J = 7.2, ^4J = 1.7 Hz, 1H), 2.69 (ddd, ^2J = 14.8, ^3J = 7.2, ^4J = 1.4 Hz, 1H), 1.77 – 1.71 (m, 2H), 1.68 – 1.61 (m, 2H), 1.44 – 1.36 (m, 1H); ^{13}C NMR (126 MHz, CD_2Cl_2): 147.8 (C), 129.6 (CH), 128.2 (CH), 126.3 (CH), 82.7 (CH), 70.8 (CH), 68.8 (CH_2), 56.8 (C), 44.8 (CH_2), 27.7 (CH_2), 26.5 (CH_2); HRMS (ESI): calcd. for $C_{25}H_{26}O_2$ ($[M+Na]^+$): 381.1825, found: 381.1813.

2.6. Synthesis of Anion-π Catalysts

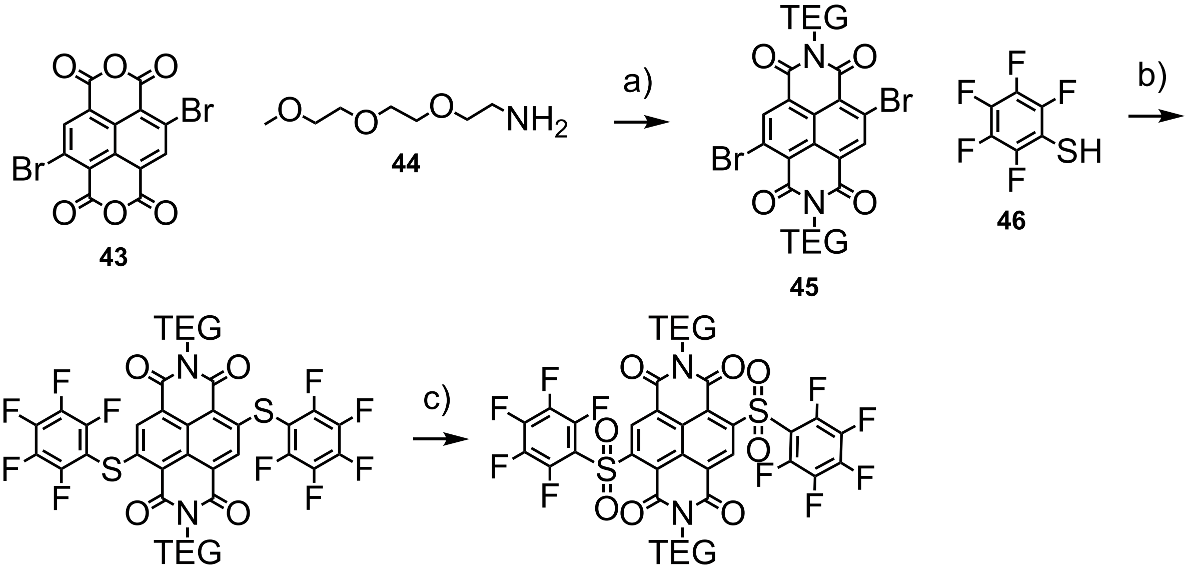

Scheme S6. a) **43**, AcOH, μW, 130 °C, 20 min, 35%; b) **46**, Cs_2CO_3, $CHCl_3$, μW, 85 °C, 1 h, 55%; c) *m*-CPBA, CH_2Cl_2, RT, 15 h, 19%.

Compound 43 was prepared following previously reported procedures as described in ref. S10.

Compound 45. To a suspension of compound **43** (0.43 g, 1.0 mmol) in acetic acid (8.00 mL) in a microwave vial, **44** (0.33 g, 2.00 mmol) was added slowly. Then the mixture was heated at 130 °C for 20 min in a sealed vessel under microwave irradiation. After cooled to room temperature, the mixture was partitioned into CH_2Cl_2 (50 mL) and H_2O (50 mL) phases. The organic phase was washed with brine (3 × 50 mL), dried over Na_2SO_4, concentrated under reduced pressure and purified by flash column chromatography (CH_2Cl_2/acetone 5:1 to 3:1) to give **45** as an orange solid (0.25 g, 35%). R_f (CH_2Cl_2/acetone 3:1): 0.47; Mp: 139 – 141 °C; IR (neat): 2872 (w), 1703 (m), 1654 (s), 1435 (m), 1106 (s), 1076 (m), 786 (m); ^{1}H NMR (300 MHz, CD_2Cl_2): 8.92 (s, 2H), 4.37 (t, ^3J = 6.0 Hz, 4H), 3.74 (t, ^3J = 5.9 Hz, 4H), 3.59 – 3.56 (m, 4H), 3.52 – 3.47 (m, 4H), 3.46 – 3.44 (m, 4H), 3.40 – 3.32 (m, 4H), 3.21 (s, 6H); ^{13}C NMR (101 MHz, CD_2Cl_2): 161.3 (CO), 161.2 (CO), 139.3

(CH), 128.5 (C), 128.2 (C), 125.8 (CBr), 124.6 (C), 72.3 (CH_2), 70.8 (CH_2), 70.8 (CH_2), 70.7 (CH_2), 68.0 (CH_2), 59.0 (CH_3), 40.5 (CH_2); LC-MS (ESI+): 717 (100, $[M+H]^+$).

Compound 47. To a suspension of compound **45** (0.15 g, 0.21 mmol), Cs_2CO_3 (1.02 g, 3.14 mmol) in $CHCl_3$ (7.98 mL) in a microwave vial, **46** (0.26 mL, 2.09 mmol) was added slowly. Then the mixture was heated at 85 °C for 40 min in a sealed vessel under microwave irradiation. The mixture was filtered, then the filtrate was concentrated and purified by flash column chromatography (CH_2Cl_2/acetone 12:1 to 4:1) to give product **47** (110.2 mg, 55%) as an orange to red solid. R_f (CH_2Cl_2/acetone 10:1): 0.54; Mp: 127 – 128 °C; IR (neat): 2877 (w), 1701 (m), 1658 (m), 1491 (s), 1316 (m), 1093 (s), 983 (s), 855 (m); ^{1}H NMR (300 MHz, CD_2Cl_2): 7.97 (s, 2H), 4.35 (t, ^3J = 5.9 Hz, 4H), 3.73 (t, ^3J = 6.0 Hz, 4H), 3.58 – 3.55 (m, 4H), 3.50 – 3.46 (m, 4H), 3.45 – 3.43 (m, 4H), 3.39 – 3.31 (m, 4H), 3.20 (s, 6H); ^{19}F NMR (282 MHz, CD_2Cl_2): -130.5 – -130.6 (m, 4F), -147.2 – -147.4 (m, 2F), -158.6 – -158.9 (m, 4F); ^{13}C NMR (101 MHz, CD_2Cl_2): 164.0 (CO), 162.0 (CO), 148.5 (d, $^1J_{C\text{-}F}$ = 246.4 Hz, CF), 144.7 (CS), 144.0 (d, $^1J_{C\text{-}F}$ = 260.6 Hz, CF), 138.8 (d, $^1J_{C\text{-}F}$ = 252.5 Hz, CF), 129.0 (CH), 126.3 (C), 125.2 (C), 120.7 (C), 105.7 (t, $^2J_{C\text{-}F}$ = 21.9 Hz, CS), 72.2 (CH_2), 70.8 (CH_2), 70.7 (CH_2), 68.0 (CH_2), 59.0 (CH_3), 40.4 (CH_2); LC-MS (ESI+): 955 (100, $[M+H]^+$).

Compound 35. To a solution of compound **47** (0.05 g, 0.05 mmol) in CH_2Cl_2 (3.00 mL), *m*-CPBA (0.13 g, 0.52 mmol) was added. Then the mixture was stirred at room temperature for 10 days. The mixture was concentrated (water bath ≤ 30 °C) then purified by flash column chromatography (CH_2Cl_2/CH_3OH 15:1 to 7:1) to give product **35** (9.7 mg, 19%) as a yellow solid. R_f (CH_2Cl_2/CH_3OH 10:1): 0.61; Mp: 244 – 246 °C; IR (neat): 2877 (w), 1720 (w), 1670 (m), 1499 (m), 1157 (m), 1096 (s), 989 (m); ^{1}H NMR (300 MHz, CD_2Cl_2): 9.69 (s, 2H), 4.33 (s, 4H), 3.71 (t, ^3J = 5.7 Hz, 4H), 3.57 – 3.49 (m, 4H), 3.45 – 3.44 (m, 4H), 3.42 – 3.41 (m, 4H), 3.37 – 3.28 (m, 4H), 3.19 (s, 6H); ^{19}F NMR (282 MHz, CD_2Cl_2): -136.9 – -136.9 (m, 4F), -144.9 – -145.1 (m, 2F), -159.5 – -159.6 (m, 4F); ^{13}C NMR (126 MHz, CD_2Cl_2, -30 °C): 160.3 (CO), 146.4 (CS), 144.9 (CF, $^1J_{C\text{-}F}$ = 259.6 Hz), 144.6 (CF, $^1J_{C\text{-}F}$ = 263.3 Hz), 137.6 (CF, $^1J_{C\text{-}F}$ = 255.8 Hz), 132.6 (CH), 129.6 (C), 127.9 (C), 125.6 (C), 117.1

(CS), 71.7 (CH_2), 70.2 (CH_2), 70.1 (CH_2), 69.7 (CH_2), 67.2 (CH_2), 58.7 (CH_3), 40.0 (CH_2); HRMS (ESI+) calc. for $C_{40}H_{32}F_{10}N_2O_{14}S_2$ $[M+Na]^+$: 1041.1028, found: 1041.0986. *Note*: ^{13}C NMR spectrum was measured at -30 °C to enhance NDI signals.

3. Catalysis

3.1. Reaction Kinetics Measurements by NMR Spectroscopy

Mixtures of substrate (**20-21**) and catalyst (**32-35** or C_6F_6) were prepared in different solvents under an ambient atmosphere in a closed 1.5 mL glass vial and stirred at RT or 10 °C. Aliquots (1 drop ~ 5 μL) of the mixture were taken at varying time intervals using a glass Pasteur pipette and ^1H NMR spectra of the diluted sample in CD_2Cl_2 were recorded (Figures S3-S12). The substrate conversion was determined by comparing the integral of pertinent resonance (**20**: 2.84 ppm and **21**: 3.57 ppm) with that of the corresponding product (**23**: 3.04 ppm and **24**: 3.65-3.79 ppm) in the crude NMR spectrum. In all cases, the starting material was converted only into Baldwin cyclic ethers. Plots of substrate conversion against reaction time show the effects of catalyst loading and reactivities of different anion-π catalysts (Figures 2A-E and 3A).

3.2. Reaction Kinetics Analysis

The obtained reaction kinetics were analyzed for autocatalysis.[S11] Assuming pseudo-first-order conditions, the reaction rate (r) can be expressed as

$$r = k^1_{cat}[R] + k^1_{ac}[R][P] \qquad (S1)$$

where k^1_{cat} and k^1_{ac} are the rate constants corresponding to the non(auto)catalytic and the (auto)catalytic mechanisms, respectively. Assuming first order in both reactant (R) and autocatalytic product (P), and

$$[P] = [R]_0 - [R] \qquad (S2)$$

then,

$$[P] = [R]_0 \times \left(1 - \frac{b+k_{cat}^1}{b+k_{cat}^1 \exp(k_{cat}^1+b)t}\right) \quad (S3)^{S12}$$

where,

$$b = [R]_0 k^1_{ac} \quad (S4)$$

The rate constants k^1_{cat} and k^1_{ac} were obtained by fitting the data to Equations (S3) and (S4) and converted to k_{cat} and k_{ac}, respectively, by dividing by the catalyst concentrations. The substrate half-life times ($t_{1/2}$) were estimated using Equation (S5).

$$t_{1/2} = \ln(b/k^1_{cat}+2)/(b+k^1_{cat}) \quad (S5)$$

3.3. Dependence on Solvents

General procedure. Solutions of substrate **20** (840 mM) and anion-π catalyst **32** (42 mM) in DMSO-d_6, CD_3CN, $(CD_3)_2CO$, THF-d_8, CD_2Cl_2, dry CH_2Cl_2 or C_6F_6 (0.1 mL) were stirred at room temperature in a closed 1.5 mL glass vial.

Table S1. Reaction kinetics for substrate **20** with catalyst **32** in different solvents.[a]

Entry	Solvent[b]	$t_{1/2}$[c] (h)	k_{cat}[d] (10^{-5} M^{-1} s^{-1})	k_{ac}[e] (10^{-4} M^{-2} s^{-1})	k_{ac} / k_{cat}[f] (M^{-1})
1	DMSO-d_6	156	1.30 ± 0.05	0.86 ± 0.03	6.6 ± 0.5
2	CD_3CN	417	0.72 ± 0.04	0.18 ± 0.03	2.5 ± 0.6
3	$(CD_3)_2CO$	642	0.22 ± 0.02	0.29 ± 0.03	13 ± 2
4	THF-d_8	634	0.17 ± 0.02	0.34 ± 0.05	20 ± 6
5	CD_2Cl_2	130	1.2 ± 0.3	1.3 ± 0.2	11 ± 4
6	C_6F_6	10	44 ± 8	2 ± 3	0.4 ± 0.8

[a]Conditions: Solvent screening of substrate **20** (840 mM) with catalyst **32** (42 mM) at RT. [b]Solvent. [c]Reaction half-lifetime. [d]Non-autocatalytic rate constant. [e]Autocatalytic rate constant. [f]Autocatalytic rate enhancement.

3.4. Dependence on Catalyst Concentration

Solutions of substrate **20** or **21** (500 mM) and anion-π catalyst **32** or **33** (25 mM) in CD_2Cl_2 (0.1 mL) were stirred at room temperature in a closed 1.5 mL glass vial.

Table S2. Reaction kinetics for substrate **20** with catalyst **32** at different concentrations.[a]

Entry	[cat][b] (mM)	$t_{1/2}$[c] (h)	k^1_{cat}[d] (10^{-7} s^{-1})	k^1_{ac}[e] (10^{-6} M^{-1} s^{-1})	k_{ac} / k_{cat}[f] (M^{-1})
1	12.5	264	2.0 ± 0.1	5.3 ± 0.2	26 ± 2
2	25	173	4.0 ± 0.4	6.7 ± 0.6	17 ± 3
3	50	88	12 ± 1	8.5 ± 0.8	7 ± 1
4	75	79	8 ± 1	15 ± 1	19 ± 4
5	100	66	10 ± 1	18 ± 2	17 ± 3
6	150	42	21 ± 1	22 ± 2	10 ± 1

[a]Conditions: Substrate **20** (500 mM), catalyst **32** (concentrations as indicated in column [cat]) in CD_2Cl_2 at RT. [b]Catalyst concentration. [c]Reaction half-life time. [d]Non-autocatalytic pseudo first-order rate constant. [e]Autocatalytic pseudo second-order rate constant. [f]Autocatalytic rate enhancement.

Table S3. Reaction kinetics for substrate **20** with catalyst **33** at different concentrations.[a]

Entry	[cat][b] (mM)	$t_{1/2}$[c] (h)	k^1_{cat}[d] (10^{-7} s^{-1})	k^1_{ac}[e] (10^{-6} M^{-1} s^{-1})	k_{ac} / k_{cat}[f] (M^{-1})
1	12.5	987	0.80 ± 0.05	1.1 ± 0.1	13 ± 3
2	25	726	0.71 ± 0.06	2.0 ± 0.1	28 ± 4
3	50	758	0.63 ± 0.06	2.0 ± 0.2	32 ± 6
4	100	426	0.60 ± 0.06	4.7 ± 0.2	80 ± 10
5	150	397	1.9 ± 0.2	2.7 ± 0.2	14 ± 2

[a]Conditions: Substrate **20** (500 mM), catalyst **33** (concentrations as indicated in column [cat]) in CD_2Cl_2 at RT. [b]Catalyst concentration. [c]Reaction half-lifetime. [d]Non-autocatalytic pseudo first-order rate constant. [e]Autocatalytic rate constant. [f]Autocatalytic rate enhancement.

Table S4. Reaction kinetics for substrate **21** with catalyst **32** at different concentrations.[a]

Entry	[Cat][b] (mM)	$t_{1/2}$[c] (h)	k^1_{cat} (10^{-7} s^{-1})[d]	k^1_{ac}[e] (M^{-1} s^{-1})	k_{ac} / k_{cat}[f] (M^{-1})
1	25	598	0.32 ± 0.06	3.8 ± 0.2	120 ± 30
2	100	253	2.0 ± 0.2	5.7 ± 0.4	29 ± 5

[a]Conditions: Substrate **21** (500 mM), catalyst **32** (see column [Cat]) in CD_2Cl_2 at RT. [b]Catalyst concentration. [c]Reaction half-life time. [d]Non-autocatalytic pseudo first-order rate constant. [e]Autocatalytic rate constant. [f]Autocatalytic rate enhancement.

3.5. Dependence on Temperature

Solutions of substrate **21** (500 mM) and anion-π catalyst **32** (25 mM) were mixed in 0.1 mL C_6F_6/CD_2Cl_2 (4:1) and stirred at different temperatures in a closed 1.5 mL glass vial.

Table S5. Reaction kinetics for substrate **21** with catalyst **32** at different temperatures.[a]

Entry	Temp[b]	$t_{1/2}$[c] (h)	k_{cat} (10^{-5} M^{-1} s^{-1})[d]	k_{ac}[e] (10^{-4} M^{-2} s^{-1})	k_{ac} / k_{cat}[f] (M^{-1})
1	RT	101	2.9 ± 0.4	4.4 ± 0.5	15 ± 4
2	10 °C	589	0.8 ± 0.1	0.4 ± 0.1	6 ± 2

[a]Conditions: Substrate **21** (500 mM), catalyst **32** (25 mM) in C_6F_6/CD_2Cl_2 (4/1) at different temperatures (see column Temp). [b]Temperature. [c]Reaction half-life time. [d]Non-autocatalytic rate constant. [e]Autocatalytic rate constant. [f]Autocatalytic rate enhancement.

3.6. Dependence on Anion-π Catalysts

Solutions of **20** (500 mM) and catalysts **32**, **33**, **34** or **35** (25 mM) in 0.1 mL CD_2Cl_2 (for **32**, **33**, and **35**) or DMSO-d_6 (for **34**) were stirred at room temperature in a closed 1.5 mL glass vial.

Table S6. Reaction kinetics for substrate **20** with different catalysts.[a]

Entry	Cond[b]	$t_{1/2}$[c] (h)	k_{cat}[d] (10^{-5} M^{-1} s^{-1})	k_{ac}[e] (10^{-4} M^{-2} s^{-1})	k_{ac} / k_{cat}[f] (M^{-1})
1	**32**, CD_2Cl_2	173	1.6 ± 0.2	2.7 ± 0.2	17 ± 3
2	**33**, CD_2Cl_2	726	0.28 ± 0.02	0.80 ± 0.06	28 ± 4
3	**34**, DMSO-d_6	918	0.23 ± 0.02	0.62 ± 0.04	27 ± 4
4	**35**, CD_2Cl_2	354	1.4 ± 0.2	0.6 ± 0.2	4 ± 2

[a]Conditions: Substrate **20** (500 mM), catalyst (see column cond, 25 mM) in CD_2Cl_2, DMSO-d_6 or C_6F_6 (see column Cond) at RT. [b]Catalyst and solvent. [c]Reaction half-life time. [d]Non-autocatalytic rate constant. [e]Autocatalytic rate constant. [f]Autocatalytic rate enhancement.

3.7. Dependence on Co-Catalysts

General procedure in CD_2Cl_2 with 23 as Co-Catalyst. Solutions of substrate **20** (500 mM) and different amount of the corresponding product **23** were mixed with **32** (25 mM) in CD_2Cl_2 (0.1 mL) and stirred at RT in a closed 1.5 mL glass vial. 1 drop (~ 5.0 µL) aliquots of the mixture were taken at varying time intervals using a glass Pasteur pipette and ^1H NMR spectra of the diluted sample in CD_2Cl_2 were recorded.

Table S7. Reaction kinetics for substrate **20** with catalyst **32** and co-catalyst **23**.[a]

Entry	Cond[b]	$t_{1/2}$[c] (h)	k_{cat}[d] (10^{-5} M^{-1} s^{-1})	k_{ac}[e] (10^{-4} M^{-2} s^{-1})	k_{ac} / k_{cat}[f] (M^{-1})
1	0	173	1.6 ± 0.2	2.7 ± 0.2	17 ± 3
2	0.5	146	2.6 ± 0.2	2.2 ± 0.2	9 ± 1
3	1.0	100	4.3 ± 0.5	2.8 ± 0.4	7 ± 2
4	1.5	79	6.0 ± 0.8	3.0 ± 0.6	5 ± 2
5	3.0	58	6.4 ± 0.8	5.9 ± 0.9	9 ± 3

[a]Conditions: Substrate **20** (500 mM), catalyst **32** (25 mM) in CD_2Cl_2 at RT with increasing eq. of **23** (see column Cond). [b]Equivalents of **23**. [c]Reaction half-life time. [d]Non-autocatalytic rate constant. [e]Autocatalytic rate constant. [f]Autocatalytic rate enhancement.

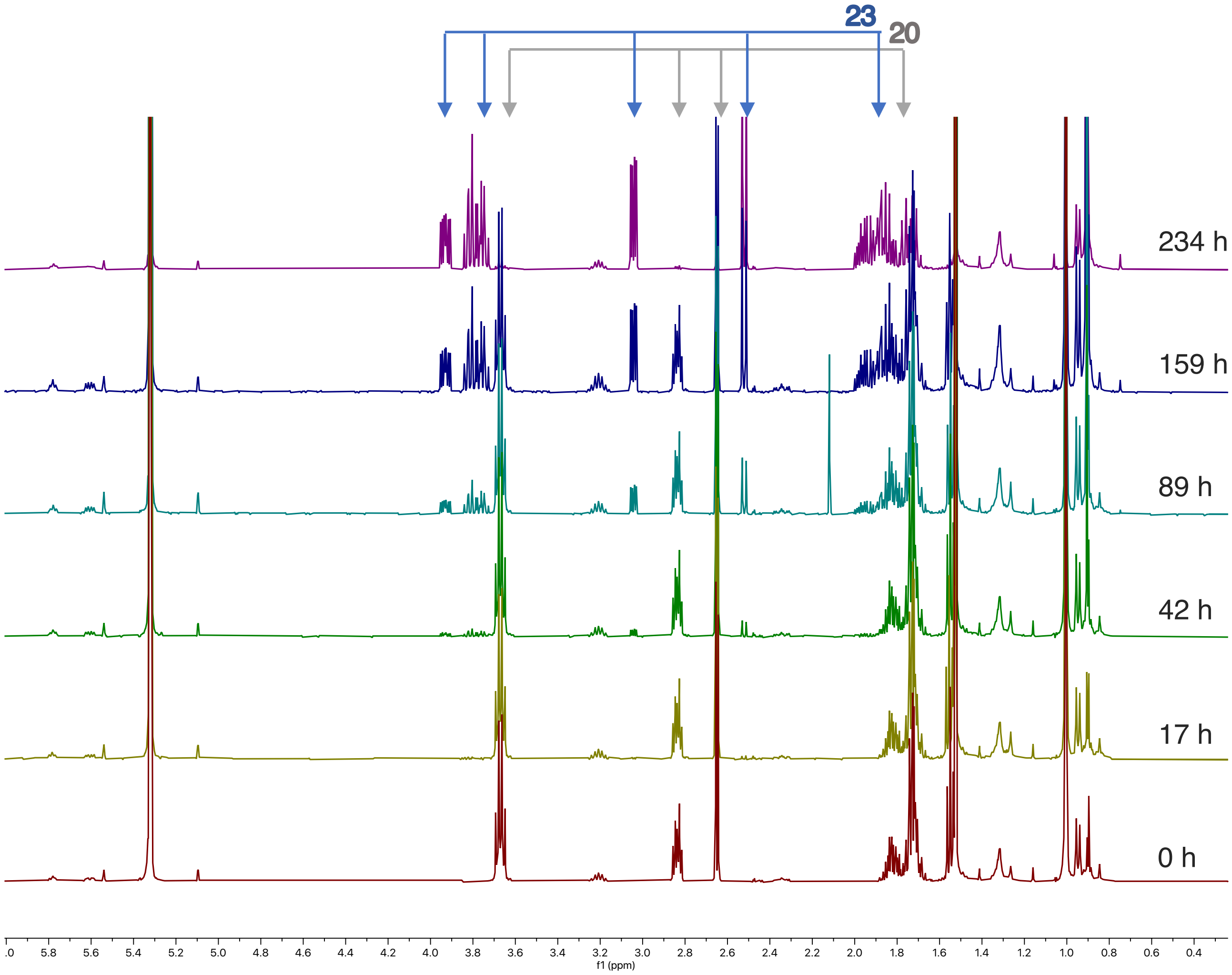

Figure S2. ^{1}H NMR spectra of reaction kinetics at room temperature for the conversion of **20** into **23** in the presence of anion-π catalyst **32** in CD_2Cl_2.

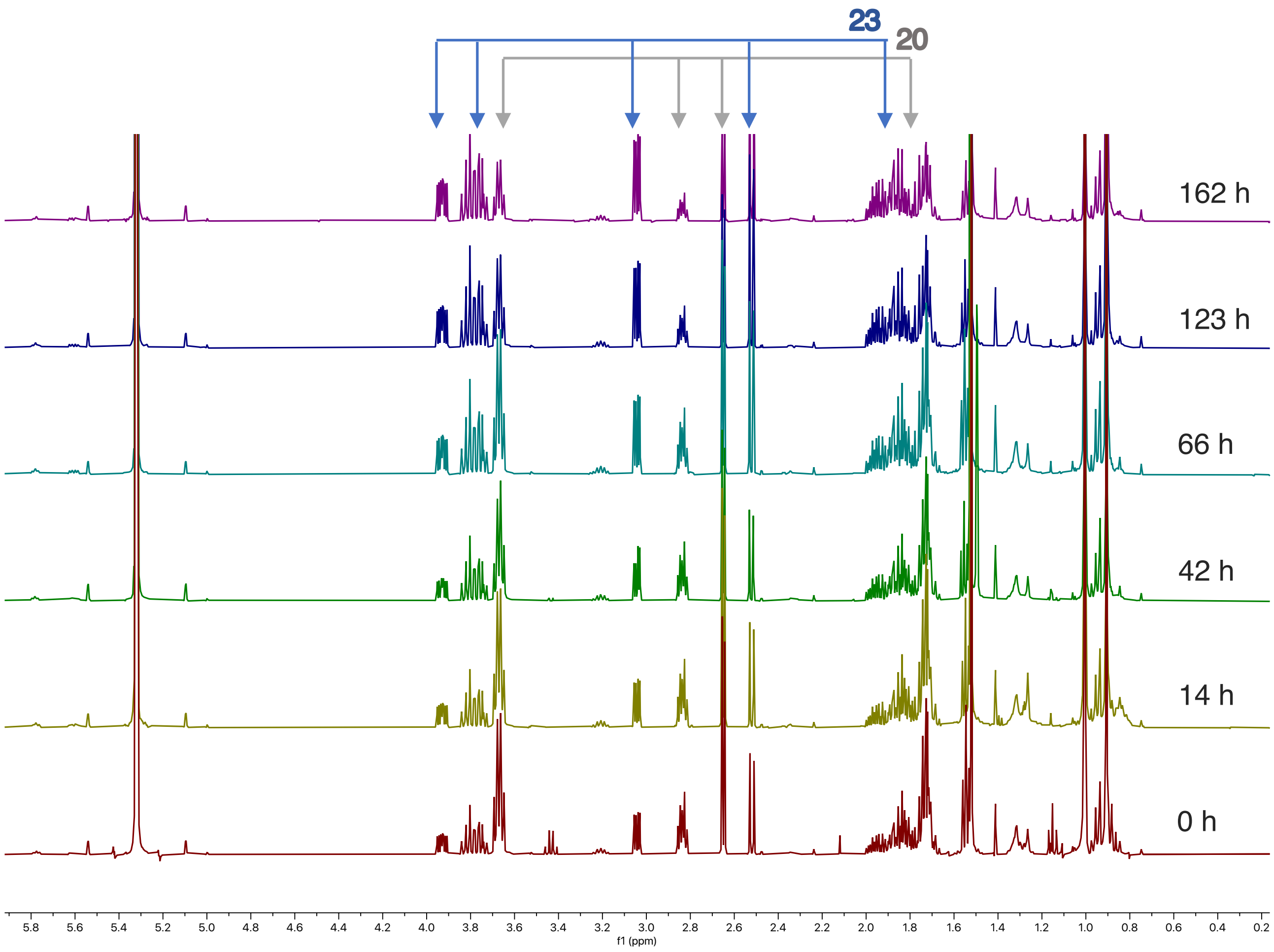

Figure S3. ^{1}H NMR spectra of reaction kinetics at room temperature for the conversion of **20** into **23** in the presence of anion-π catalyst **32** and 0.5 equivalents of **23** added at the beginning of the reaction in CD_2Cl_2.

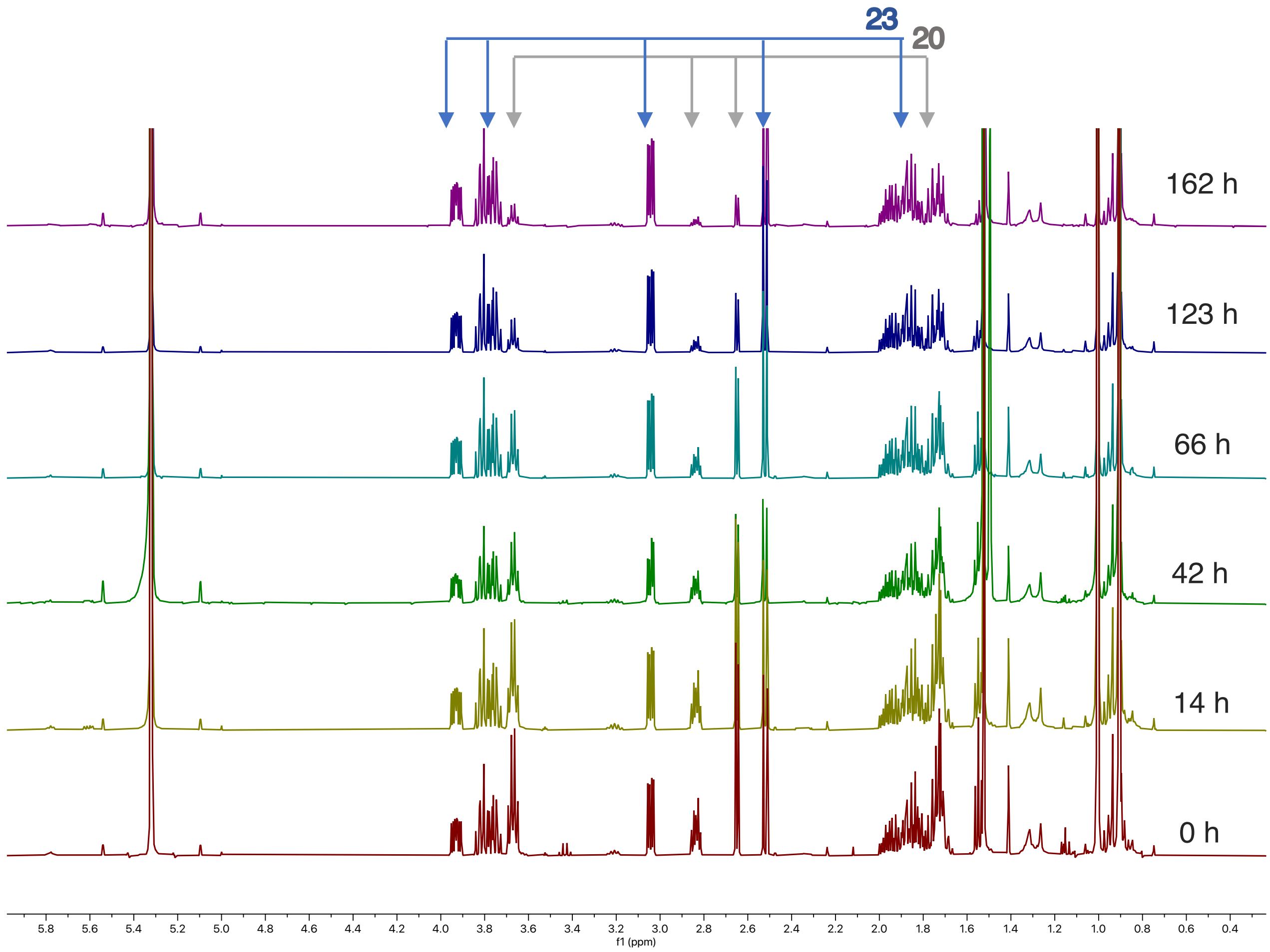

Figure S4. ^{1}H NMR spectra of reaction kinetics at room temperature for the conversion of **20** into **23** in the presence of anion-π catalyst **32** and 1.0 equivalents of **23** added at the beginning of the reaction in CD_2Cl_2.

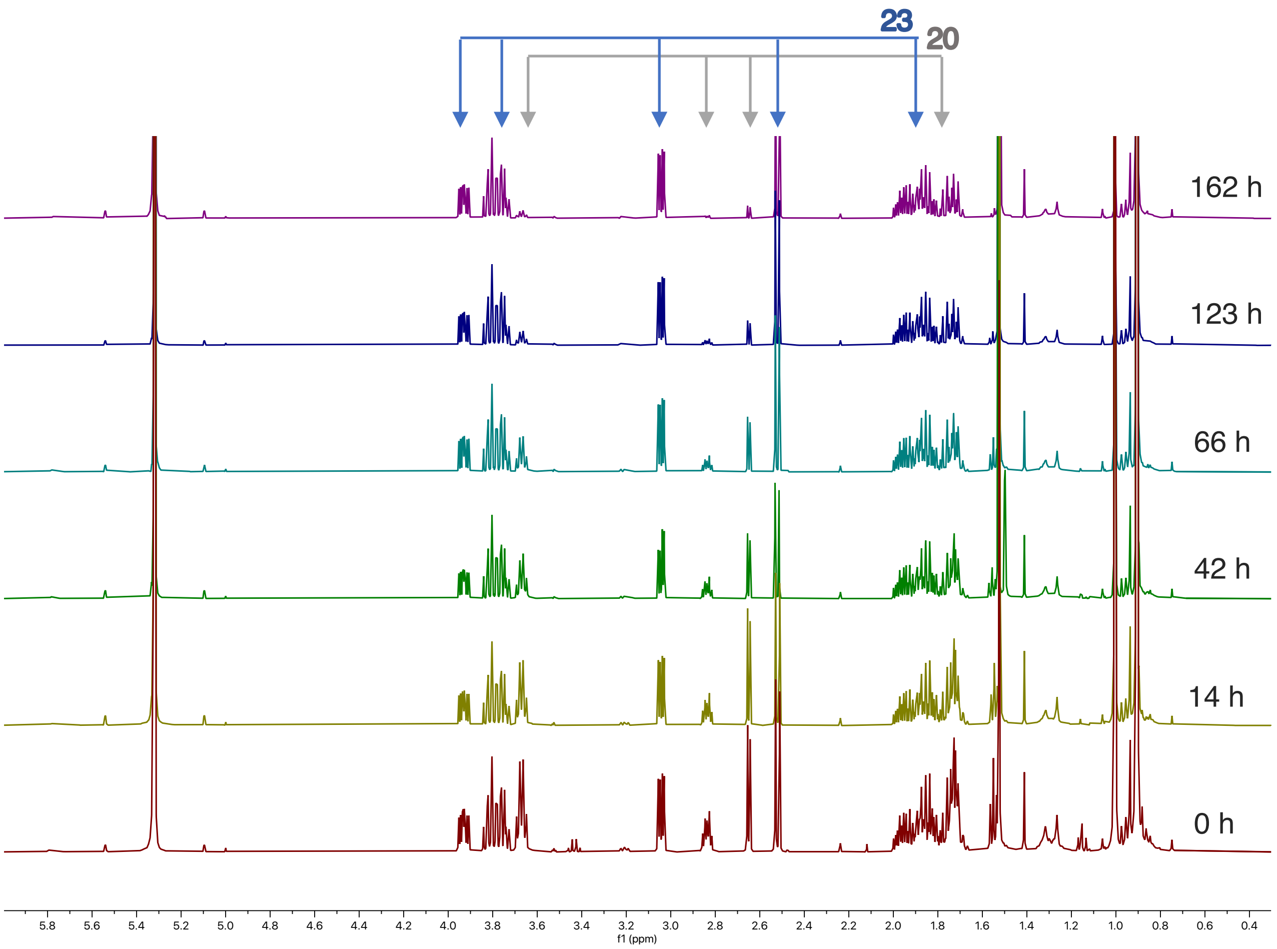

Figure S5. ^{1}H NMR spectra of reaction kinetics at room temperature for the conversion of **20** into **23** in the presence of anion-π catalyst **32** and 1.5 equivalents of **23** added at the beginning of the reaction in CD_2Cl_2.

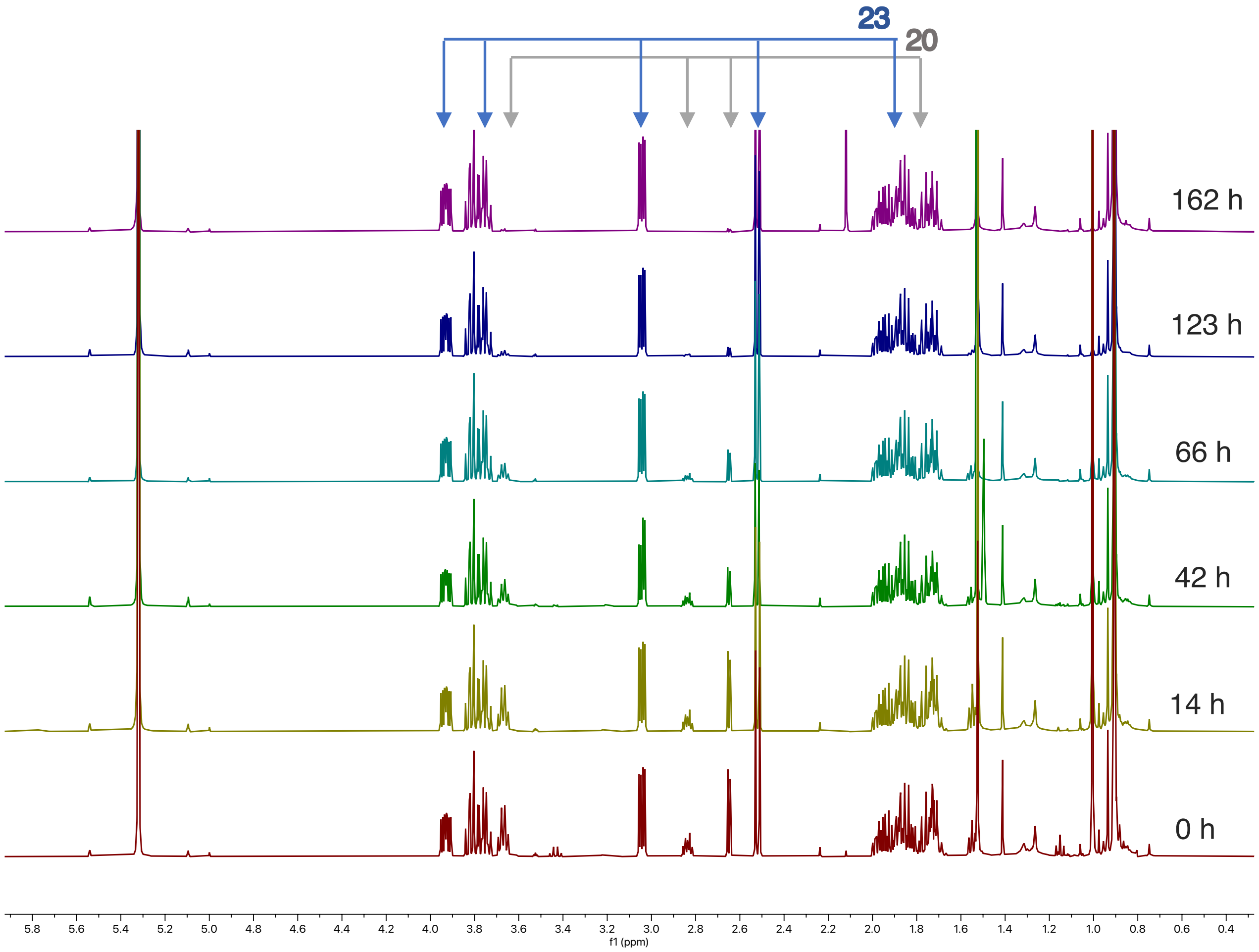

Figure S6. ^{1}H NMR spectra of reaction kinetics at room temperature for the conversion of **20** into **23** in the presence of anion-π catalyst **32** and 3.0 equivalents of **23** added at the beginning of the reaction in CD_2Cl_2.

General procedure in C_6F_6 with 23 as Co-Catalyst: Solutions of substrate **20** (840 mM) and different amount of the co-catalyst **23** were mixed with **32** (42 mM) in C_6F_6 (0.1 mL) and stirred at 10 °C in a closed 1.5 mL glass vial. 1 drop (~ 5 µL) aliquots of the mixture were taken at varying time intervals using a glass Pasteur pipette and ^{1}H NMR spectra of the diluted sample in CD_2Cl_2 were recorded.

Table S8. Reaction kinetics for substrate **20** with catalyst **32** in C_6F_6.[a]

Entry	Cond[b]	$t_{1/2}$[c] (h)	k_{cat}[d] (10^{-5} M^{-1} s^{-1})	k_{ac}[e] (10^{-4} M^{-2} s^{-1})	k_{ac} / k_{cat}[f] (M^{-1})
1	0	33	7.4 ± 0.4	3.2 ± 0.2	4.3 ± 0.5
2	0.5	49	6.2 ± 1.4	1.5 ± 0.7	3 ± 2
3	1.0	55	5.4 ± 0.6	1.4 ± 0.3	2.6 ± 0.8
4	2.0	68	3.3 ± 0.2	1.8 ± 0.1	5.5 ± 0.5

[a]Conditions: Substrate **20** (840 mM), catalyst **32** (42 mM) in C_6F_6 at 10 °C with increasing eq. of **23** (see column Cond). [b]Equivalents of **23**. [c]Reaction half-life time. [d]Non-autocatalytic rate constant. [e]Autocatalytic rate constant. [f]Autocatalytic rate enhancement.

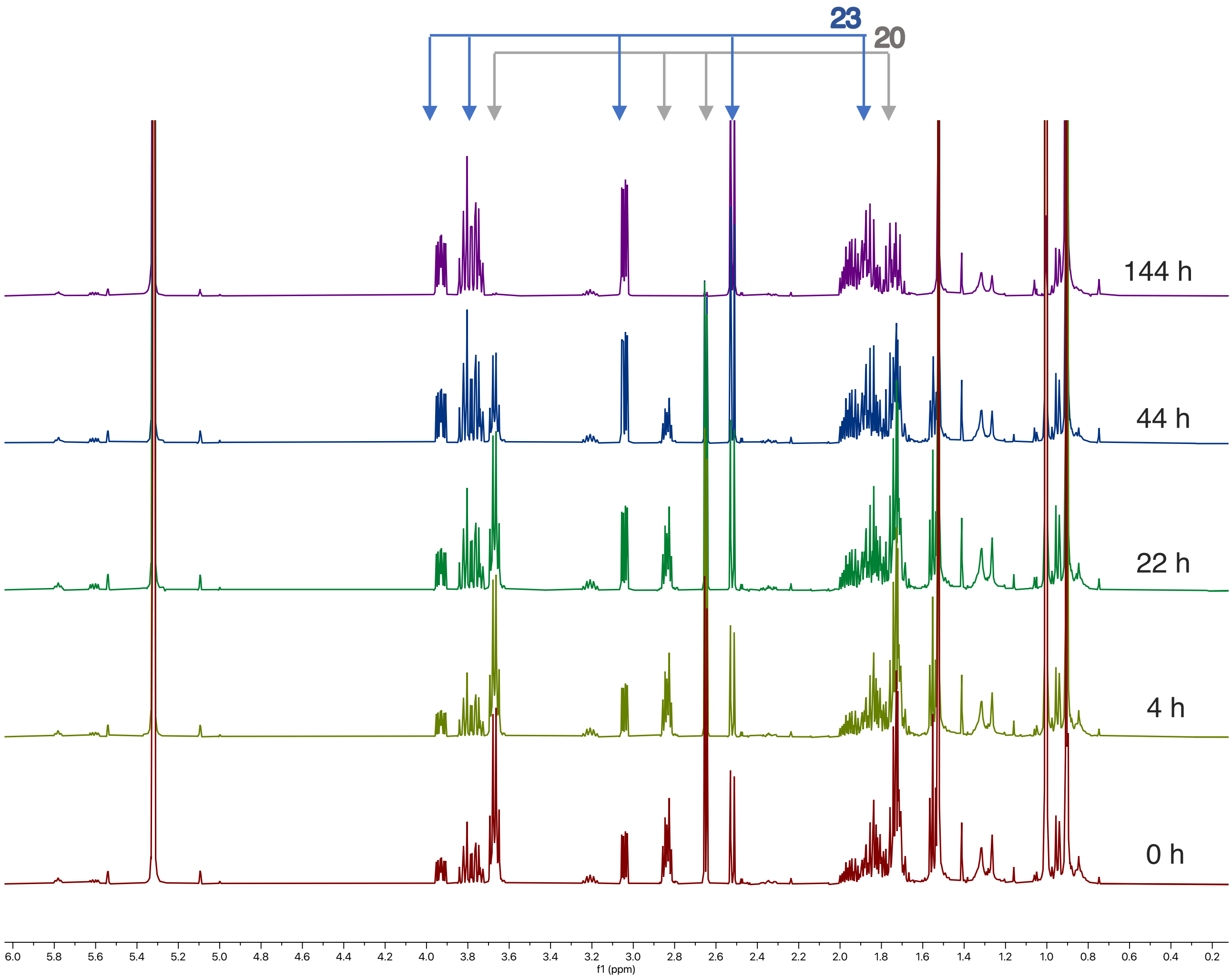

Figure S7. ^{1}H NMR spectra of reaction kinetics at 10 °C for the conversion of **20** into **23** in the presence of **32** in C_6F_6 as solvent.

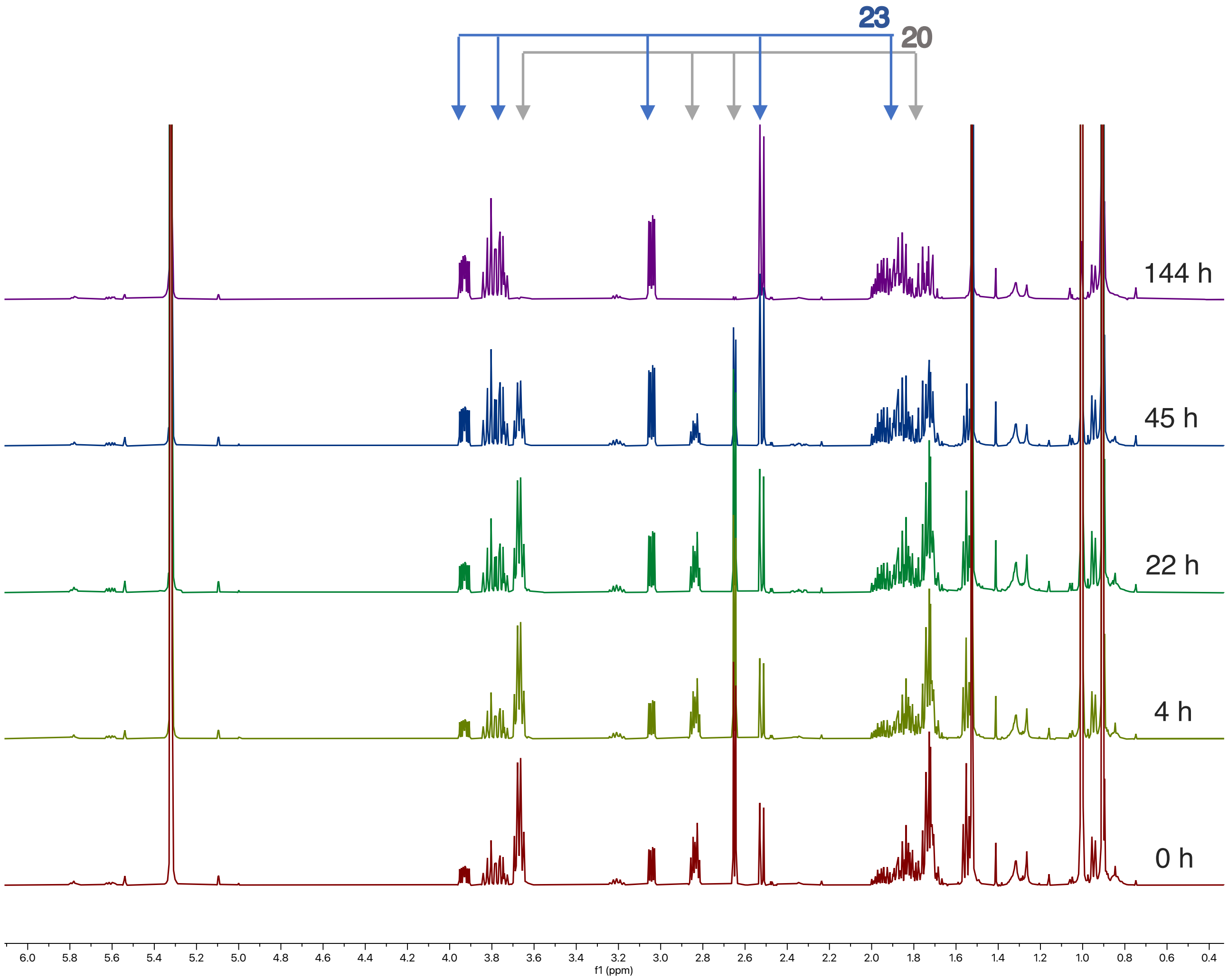

Figure S8. ^{1}H NMR spectra of reaction kinetics at 10 °C for the conversion of **20** into **23** in the presence of **32** and 0.50 equivalents of **23** added at the beginning of the reaction in C_6F_6 as solvent.

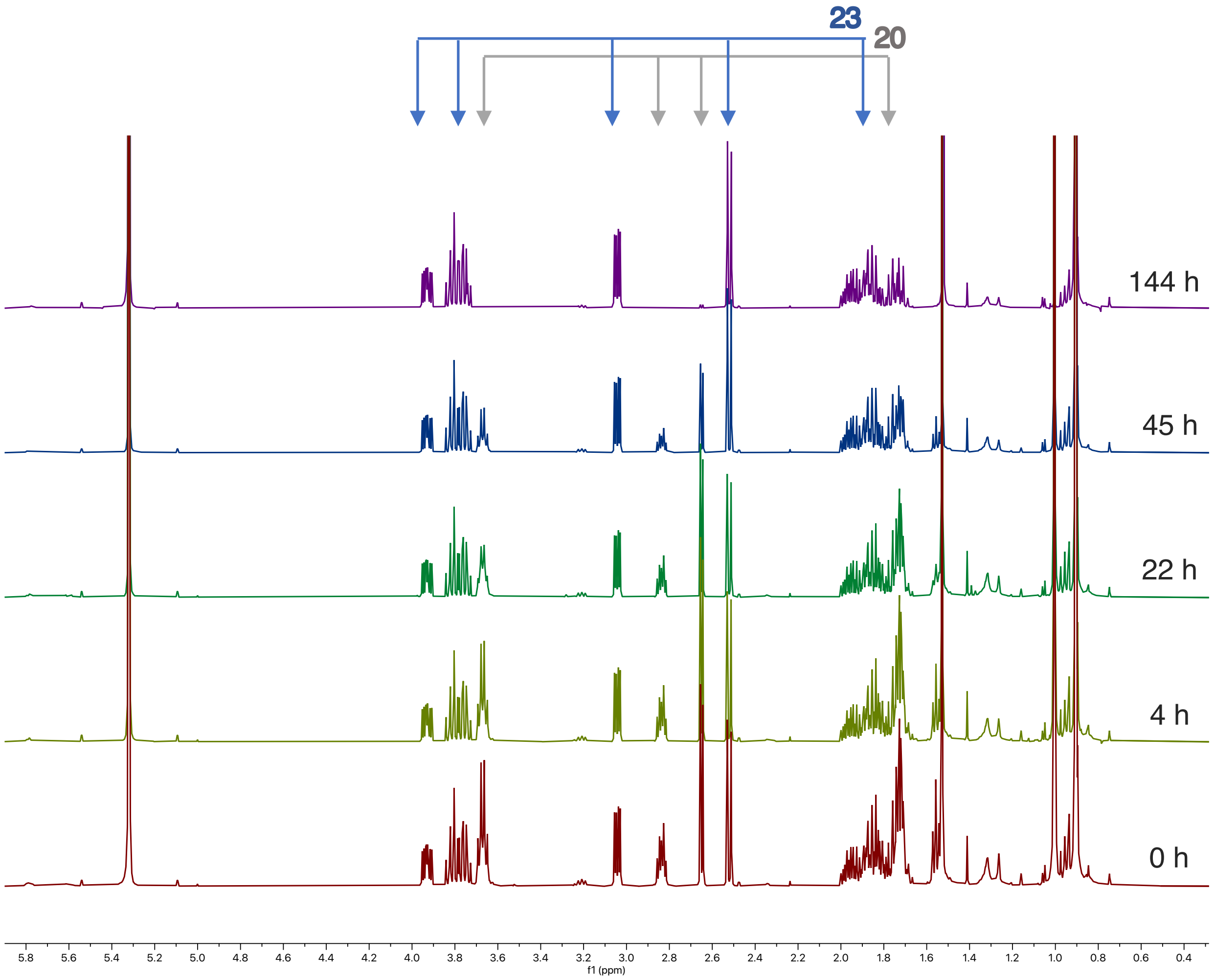

Figure S9. ^{1}H NMR spectra of reaction kinetics at 10 °C for the conversion of **20** into **23** in the presence of **32** and 1.0 equivalents of **23** added at the beginning of the reaction in C_6F_6 as solvent.

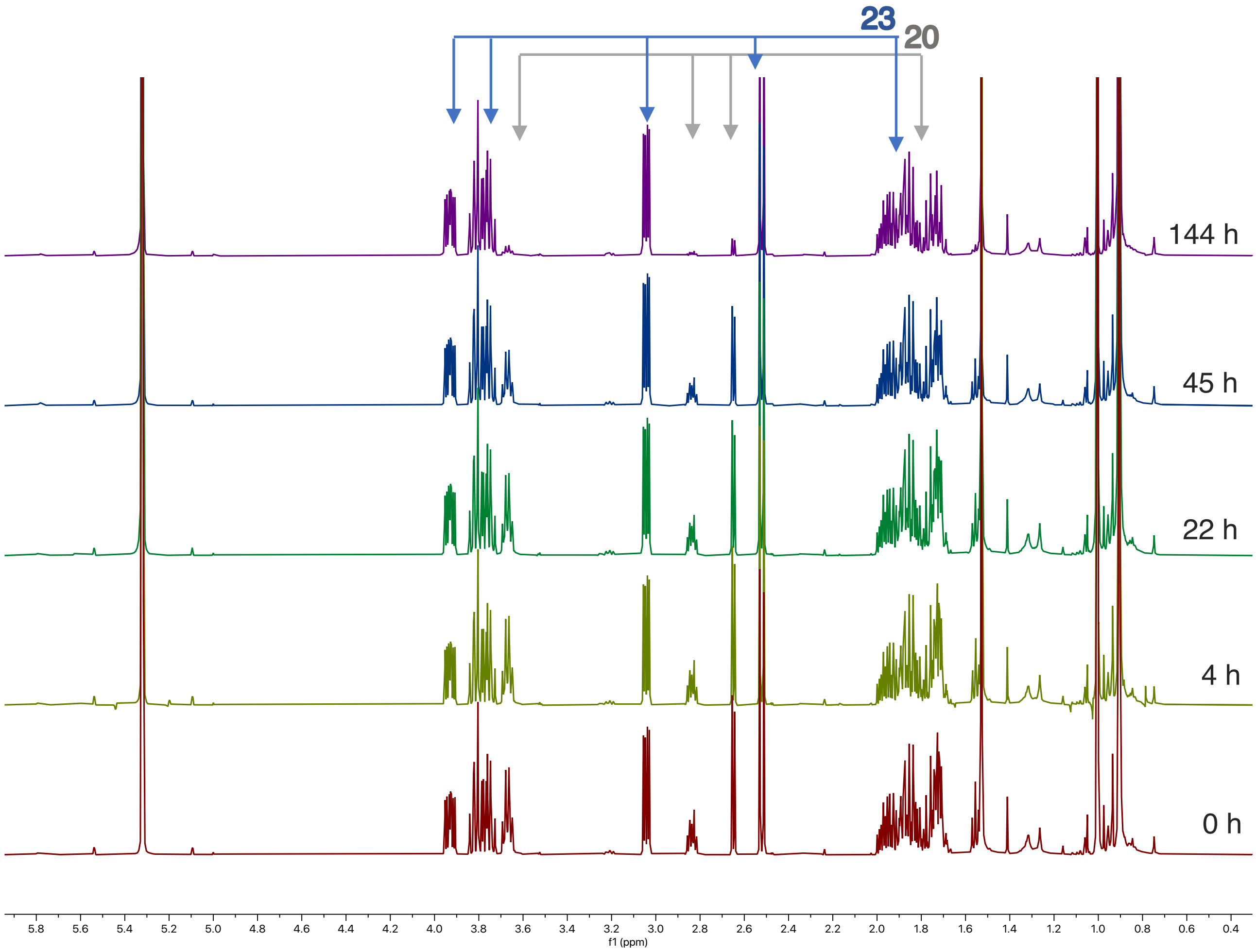

Figure S10. ^{1}H NMR spectra of reaction kinetics at 10 °C for the conversion of **20** into **23** in the presence of **32** and 2.0 equivalents of **23** added at the beginning of the reaction in C_6F_6 as solvent.

Catalysis with C_6F_6. A solution of **20** (1.0 M) in C_6F_6 (0.1 mL) was stirred at room temperature in a closed 1.5 mL glass vial. 1 drop (~ 5 µL) aliquots of the mixture were taken at varying time intervals using a glass Pasteur pipette and ^1H NMR spectra of the diluted sample in CD_2Cl_2 were recorded.

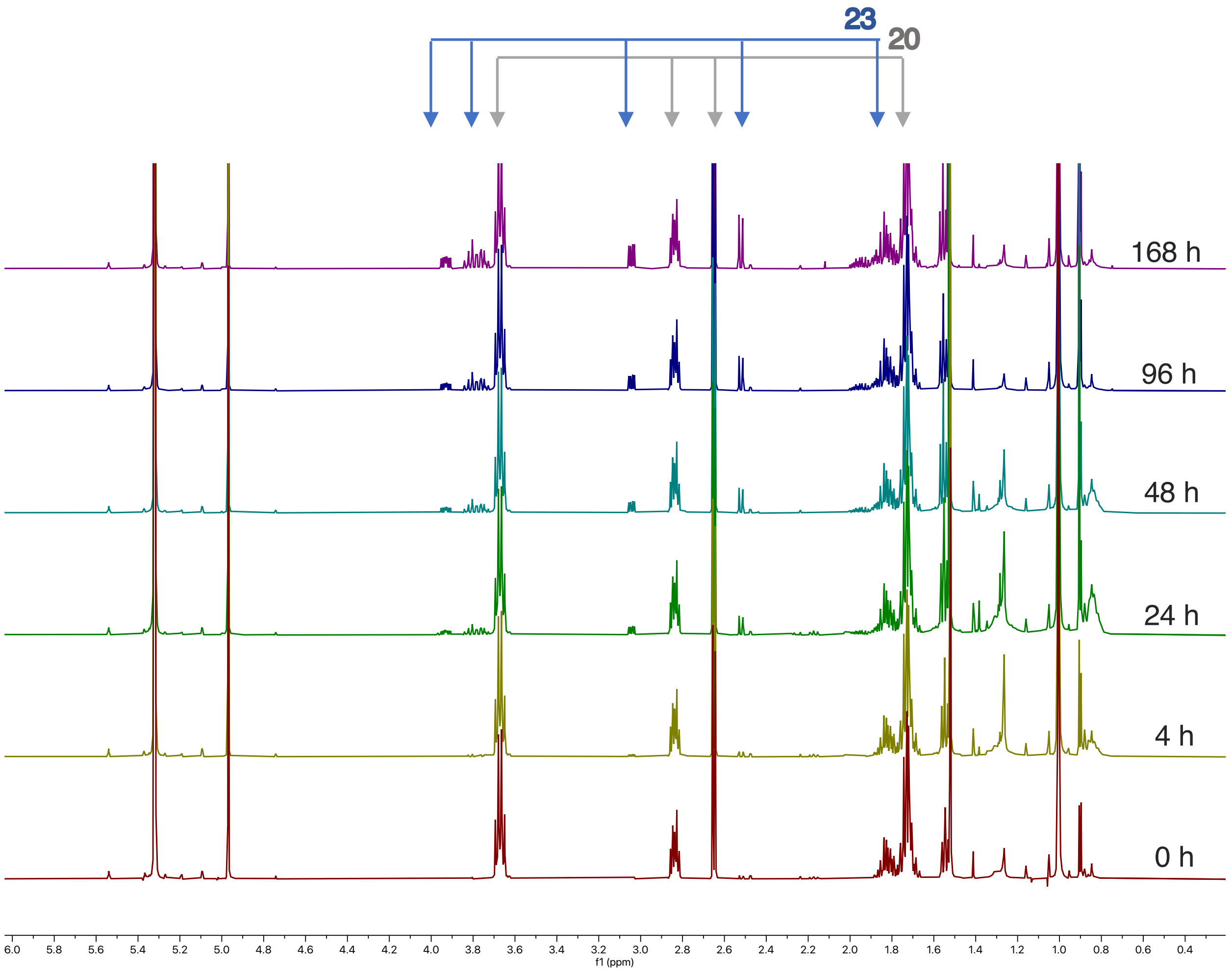

Figure S11. ^1H NMR spectra of reaction kinetics at room temperature for the conversion of **20** into **23** in C_6F_6 as solvent catalyst.

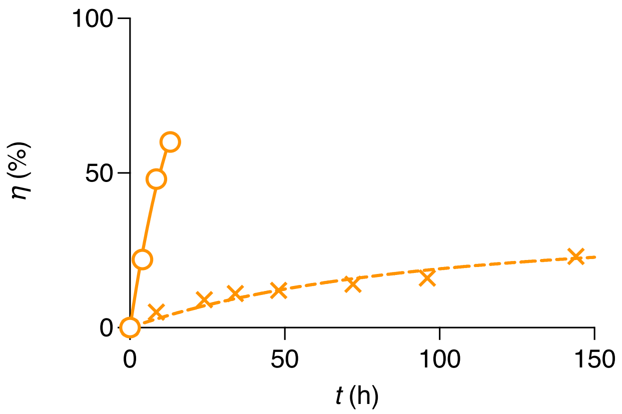

Figure S12. Time course of the conversion η of **20** (840 mM) with **32** (42 mM) in C_6F_6 (orange ○), and of **20** (1.0 M) in C_6F_6 (orange ×).

3.8. Dependence on Enantioenriched Co-Catalysts

General procedure. To a solution of (*rac*)-cis-**20** (500 mM) and increasing equivalents of enantio-enriched product (**23a** or **36**) in C_6F_6 (0.1 mL) was added **32** (25 mM), then the mixture was stirred at 10 °C in a closed 1.5 mL glass vial. 1 drop (~ 5 µL) aliquots of the mixture were taken at varying time intervals using a glass Pasteur pipette and ^{1}H NMR spectra of the diluted sample in CD_2Cl_2 were recorded to estimate the conversion. An aliquot of the reaction mixture (10 µL) was added to a glass vial with Et_2O (0.5 mL) after indicated time, then the sample was analyzed by chiral GC.

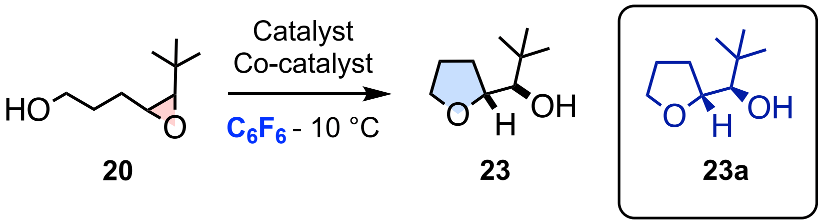

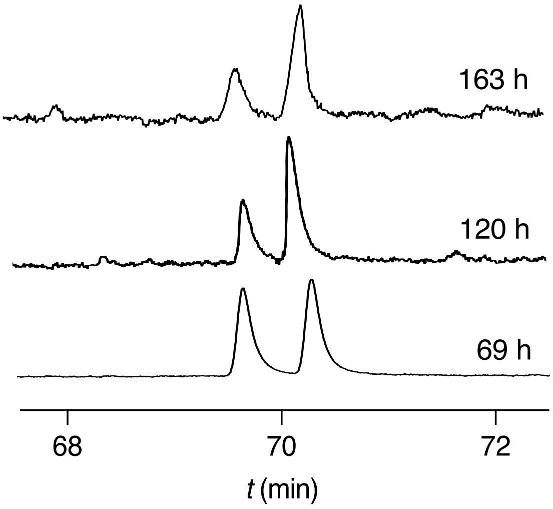

Peak #	RetTime [min]	Type	Width [min]	Area [pA*s]	Height [pA]	Area %
1	69.704	MM	0.1747	41.25670	3.93679	46.68875
2	70.360	MM	0.1823	47.10871	4.30636	53.31125
1	69.724	MM	0.2013	6.94105	5.74582e-1	33.93207
2	70.369	MM	0.1980	13.51466	1.13773	66.06793
1	69.712	MM	0.2002	4.21047	3.50600e-1	32.68267
2	70.372	MM	0.1900	8.67240	7.60834e-1	67.31733

Figure S13. Chiral GC profiles of the remaining substrate **20** after 69 h (50% conversion, 7% ee), 120 h (75% conversion, 32% ee) and 163 h (80% conversion, 35% ee) (top to bottom) in the presence of anion-π catalyst **32** and 3 equivalents of **23a** (89% ee, assuming the same value as of compound **31a**).

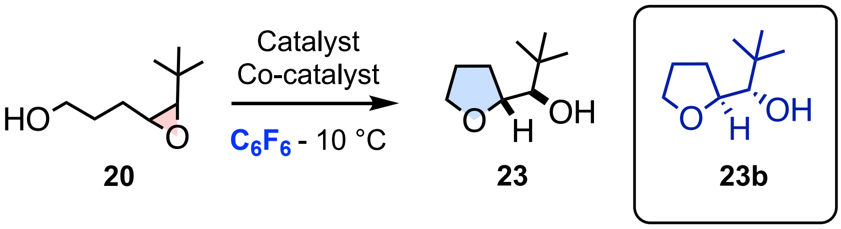

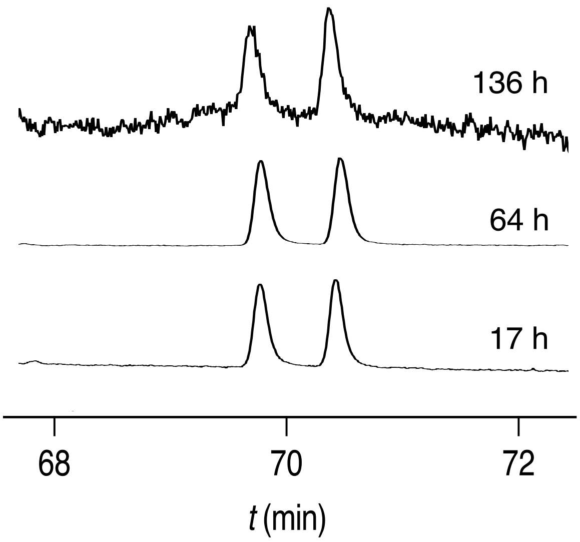

Peak #	RetTime [min]	Type	Width [min]	Area [pA*s]	Height [pA]	Area %
1	69.690	MM	0.1467	55.91029	6.35324	50.00019
2	70.328	MM	0.1445	55.90986	6.44949	49.99981
1	69.715	MM	0.1469	21.92725	2.48709	49.85146
2	70.364	MM	0.1401	22.05791	2.62437	50.14854
1	69.184	MM	0.1489	2.14617	2.40155e-1	46.90663
2	69.831	MM	0.1375	2.42924	2.94507e-1	53.09337

Figure S14. Chiral GC profiles of the remaining substrate **20** after 17 h (4% conversion, 0% ee), 64 h (35% conversion, 0% ee), and 136 h (94% conversion, 6% ee) (top to bottom) in the presence of anion-π catalyst **32** and 0.75 equivalents of **23b** (96% ee, assuming the same value as of compound **31b**).

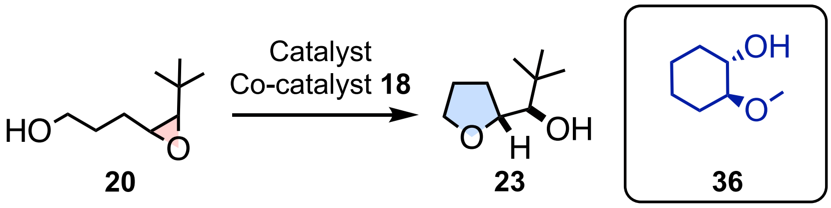

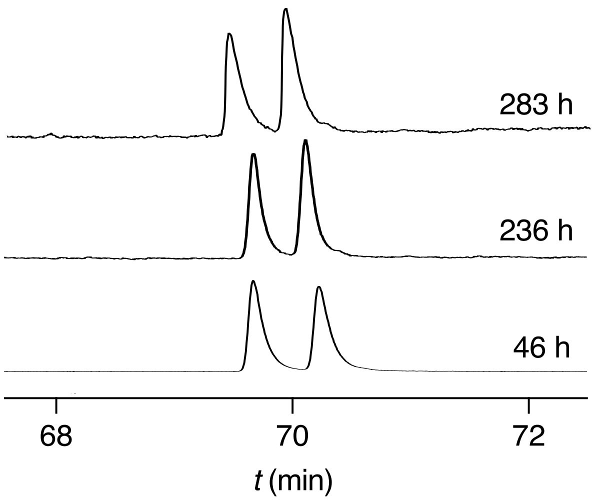

Peak #	RetTime [min]	Type	Width [min]	Area [pA*s]	Height [pA]	Area %
1	69.611	MM	0.1755	220.19875	20.90746	50.16520
2	70.272	MM	0.1898	218.74844	19.20999	49.83480
1	69.653	MM	0.1922	39.29373	3.40813	45.54902
2	70.314	MM	0.2025	46.97319	3.86668	54.45098
1	69.679	MM	0.2169	29.62707	2.27668	44.42689
2	70.323	MM	0.2193	37.06018	2.81671	55.57311

Figure S15. Chiral GC profiles of the remaining substrate **20** after 46 h (7% conversion, 0% ee), 236 h (76% conversion, 9 % ee) and 283 h (86% conversion, 11% ee) (top to bottom) in the presence of anion-π catalyst **32** and 1.50 equivalents of **36.**

General procedure in C_6F_6 with 36 as Co-Catalyst. Solutions of substrate **20** (500 mM) and different amount of the corresponding co-catalyst **36** were mixed with **32** (25 mM) in C_6F_6 (0.1 mL) and stirred at 10 °C in a closed 1.5 mL glass vial. 1 drop (~ 5 µL) aliquots of the mixture were taken at varying time intervals using a glass Pasteur pipette and ^{1}H NMR spectra of the diluted sample in CD_2Cl_2 were recorded.

Table S9. Reaction kinetics for substrate **20** with catalyst **32** and co-catalyst **36**.[a]

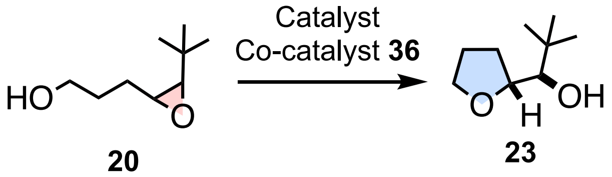

Entry	**36**[b] (eq)	$t_{1/2}$[c] (h)	k_{cat}[d] (10^{-5} M^{-1} s^{-1})	k_{ac}[e] (10^{-4} M^{-2} s^{-1})	k_{ac} / k_{cat}[f] (M^{-1})
1	0.30	125	4.0 ± 0.4	1.8 ± 0.3	4 ± 1
2	0.70	184	1.6 ± 0.1	2.4 ± 0.1	15 ± 1
3	1.50	171	1.4 ± 0.1	3.1 ± 0.2	23 ± 3
4	3.70	250	0.9 ± 0.1	2.2 ± 0.2	26 ± 5
5	5.00	290	0.48 ± 0.04	2.4 ± 0.1	51 ± 7
6	10.0	317	0.33 ± 0.03	2.5 ± 0.1	80 ± 10

[a]Conditions: Substrate **20** (500 mM), catalyst **32** (25 mM) in C_6F_6 at 10 °C with increasing eq. of **36**. [b]Equivalents of **36** relative to **20**. [c]Reaction half-life time. [d]Non-autocatalytic rate constant. [e]Autocatalytic rate constant. [f]Autocatalytic rate enhancement.

3.9. Dependence on Water

To solutions of **20** (500 mM) and catalyst **32** (25 mM) in CD_2Cl_2 or anhydrous CH_2Cl_2 (0.1 mL) were added increasing equivalents of water (Table S10) and the mixtures were stirred at room temperature in a closed 1.5 mL glass vial. 1 drop (~ 5 μL) aliquots of the mixture were taken at varying time intervals using a glass Pasteur pipette and ^1H NMR spectra of the diluted sample in CD_2Cl_2 were recorded.

Table S10. Reaction kinetics for substrate **20** with catalyst **32** and water as co-catalyst.[a]

Entry	Cond[b]	H_2O^e (eq)	$t_{1/2}^f$ (h)	k_{cat}^g (10^{-5} M^{-1} s^{-1})	k_{ac}^h (10^{-4} M^{-2} s^{-1})	k_{ac} / k_{cat}^i (M^{-1})
1	CH_2Cl_2[c]	-	334	0.44 ± 0.06	2.1 ± 0.2	50 ± 10
2	CH_2Cl_2[d]	-	195	0.9 ± 0.3	3.2 ± 0.5	40 ± 20
3	CD_2Cl_2	-	173	1.6 ± 0.2	2.7 ± 0.2	17 ± 3
4	CD_2Cl_2	1.0	276	1.6 ± 0.1	1.0 ± 0.1	6 ± 1
5	CD_2Cl_2	3.0	233	2.8 ± 0.1	0.41 ± 0.09	1.5 ± 0.4
6	CD_2Cl_2	10	330	0.52 ± 0.04	2.0 ± 0.1	38 ± 5
7	CD_2Cl_2	50	857	0.14 ± 0.04	0.9 ± 0.2	60 ± 30
8	CD_2Cl_2	100	1017	0.19 ± 0.02	0.59 ± 0.07	31 ± 6

[a]Conditions: Substrate **20** (500 mM), catalyst **32** (25 mM) in dry CH_2Cl_2 or CD_2Cl_2 (see column Cond) at RT with increasing eq. of H_2O (see column H_2O eq). [b]Reaction solvent. [c]CH_2Cl_2 anhydrous, 99.7+% and dried over molecular sieve type UOP 3A. [d]CH_2Cl_2 anhydrous, 99.7+%. [e]Equivalents of H_2O relative to substrate. [f]Reaction half-life time. [g]Non-autocatalytic rate constant. [h]Autocatalytic rate constant. [i]Autocatalytic rate enhancement.

4. Theoretical Methods

The energy calculations have been performed using the B3LYP functional[S13-S16] adding Grimme's D3 dispersion correction[S17] and combined with the 6-31+G* basis set,[S18] which is an

acceptable compromise between the size of the system (> 95 atoms for the ternary complexes with two water molecules) and the accuracy of the results. We have taken into consideration solvent effects (CH_2Cl_2) using a polarized continuum model (PCM)[S19] as implemented in Gaussian-16.[S20] It uses the integral equation formalism model (IEFPCM).[S21] Therefore the energies used to compute the step barriers were obtained from B3LYP-D3(solvent = dichloromethane)/6-31+G*//B3LYP-D3/6-31G* level of theory. The geometries have been fully optimized at the B3LYP-D3/6-31G* level of theory without symmetry constrains. The full conformational space has not been explored, since it is out of the scope of the present work. The efforts have been focused on the comparison of the rate determining step (epoxide opening) and using the activation barriers depending on the number of water molecules and to rationalize the experimental findings. The transition states have been characterized using the standard procedure and in all cases they present only one negative frequency connecting reactant and product (see below). The values of ΔG correspond to gas phase since the optimized geometries used for the solvent single point energy calculations were calculated in the gas phase.

4.1. Frequency Calculations and Six First Frequencies Values

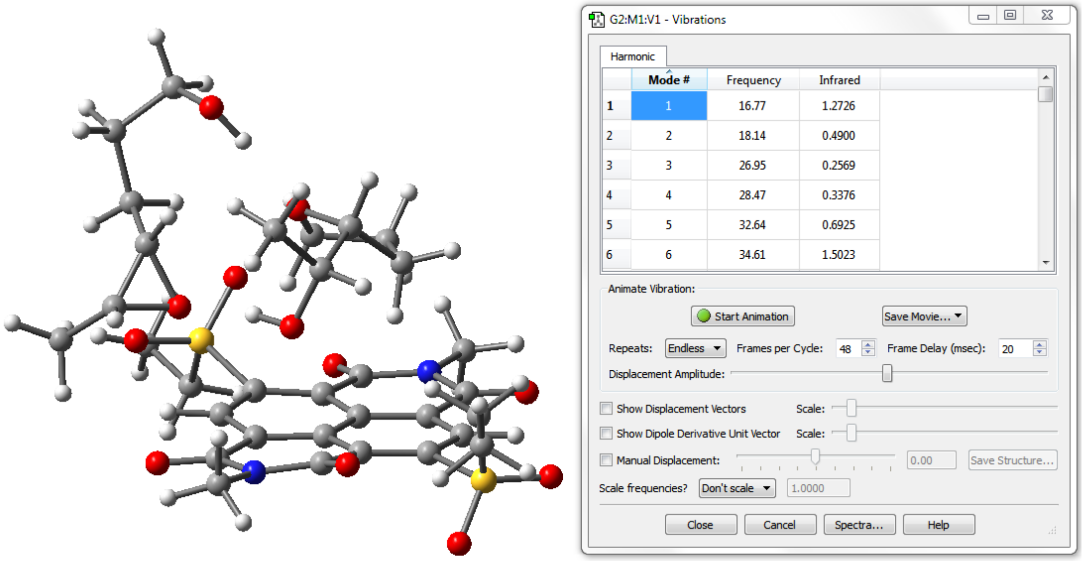

Figure S16. Starting product, 0 water molecules.

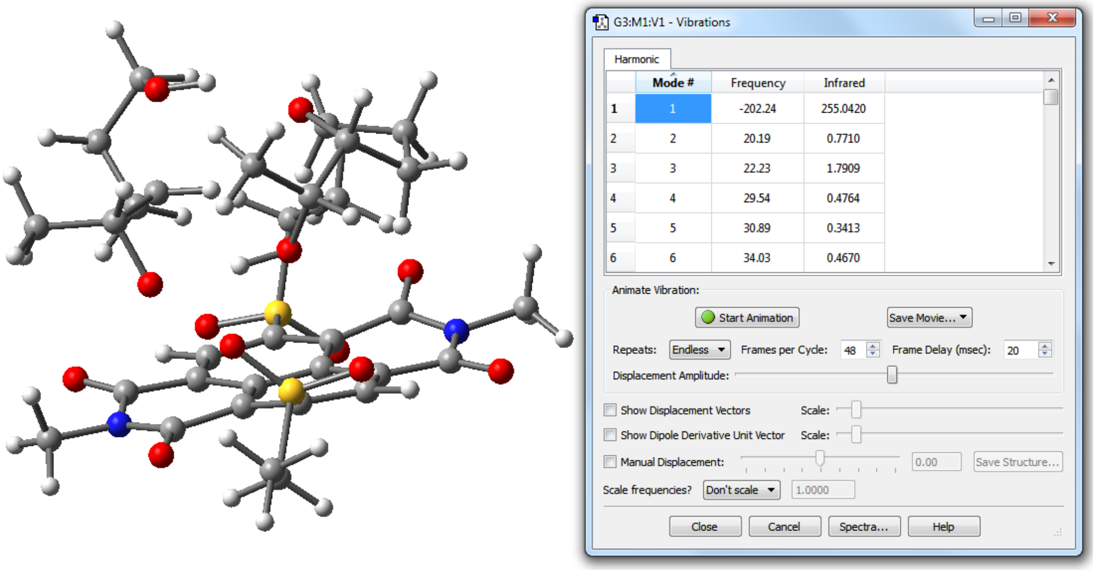

Figure S17. Transition State, 0 water molecules, imaginary frequency -202.24 cm^{-1}.

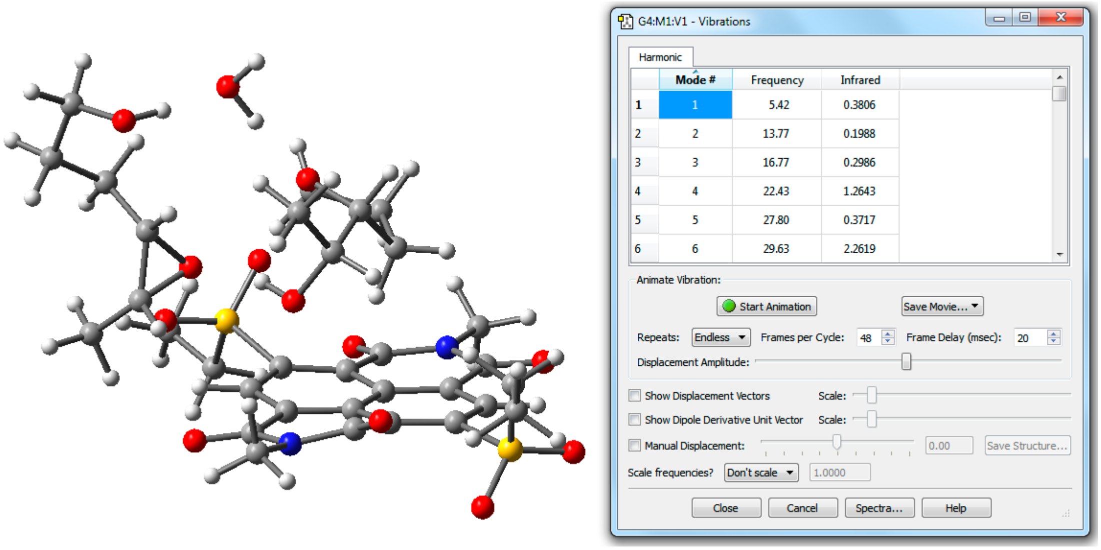

Figure S18. Starting product, 1 water molecule.

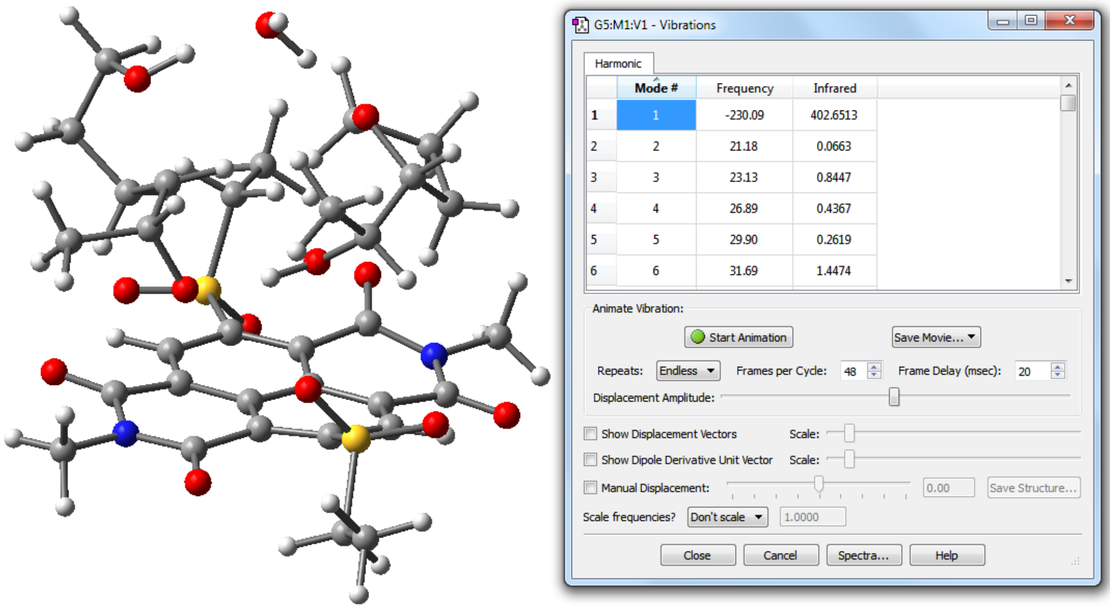

Figure S19. Transition State, 1 water molecule, imaginary frequency -230.09 cm^{-1}.

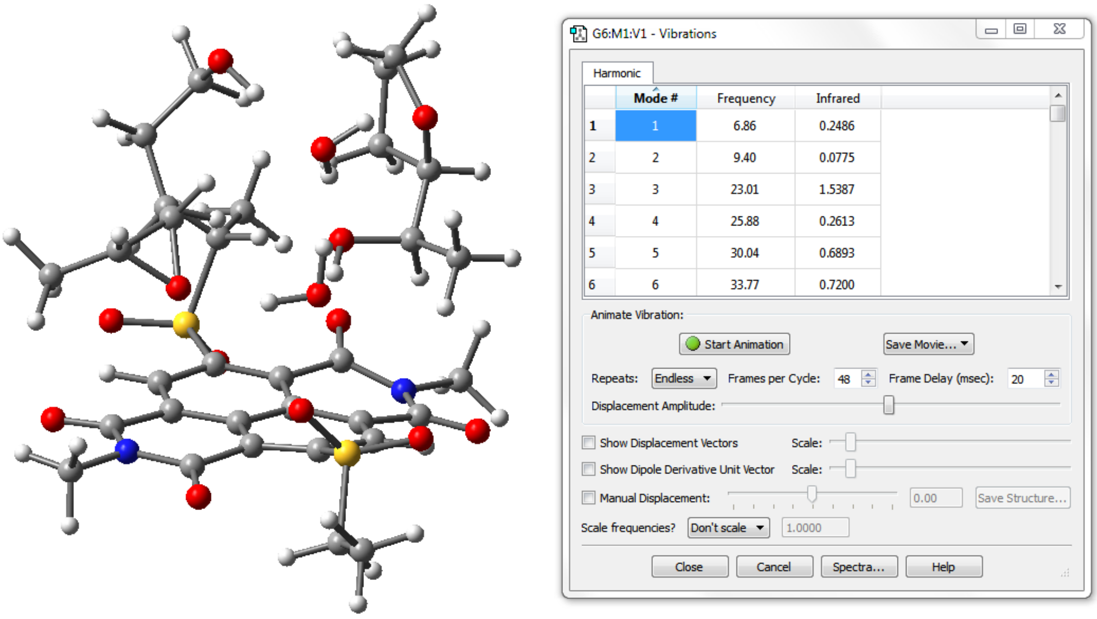

Figure S20. Starting product, 2 water molecules.

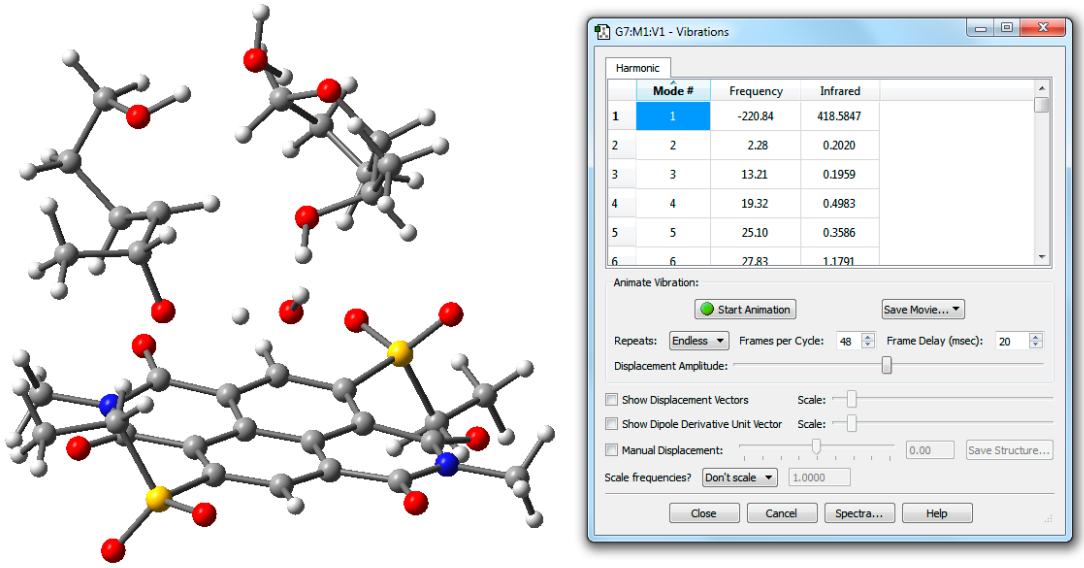

Figure S21. Transition State, 2 water molecules, imaginary frequency -220.84 cm^{-1}.

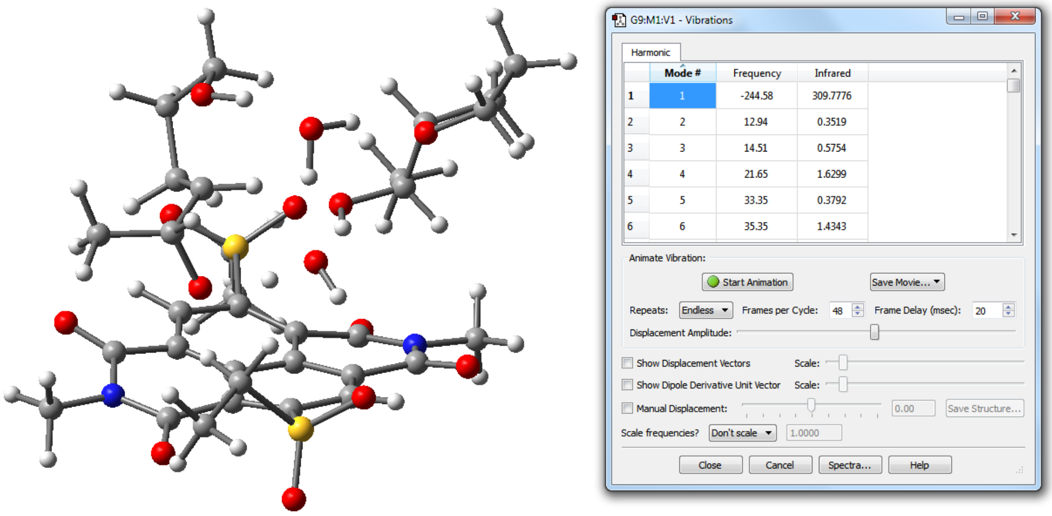

Figure S22. Transition State, 2 water molecules, S-configuration, imaginary frequency -244.58 cm^{-1}.

4.2. Gibbs Free Energies and Activation Energy Barriers

Table S11. Comparison of ΔG barriers (in the gas phase) with Ea barriers (in solvent) at the B3LYP-D3(solvent = dichloromethane)/6-31+G*//B3LYP-D3/6-31G* level of theory.[a]

Entry	TS	ΔG# (kcal mol^{-1})	Ea (kcal mol^{-1})
1	**TS-1**	+34.0	+26.1
2	**TS-7**	+20.4	+22.6
3	**TS-2**	+25.1	+20.0
4	**TS-8**	+30.2	+28.5

[a]Ea rather than ΔG barriers are discussed in the manuscript because the inclusion of solvent was considered most important.

4.3. Cartesian Coordinates, Gibbs free energies and ZPE values in Hartree

Starting product 0 water molecules
G = -3052.676346; ZPE = 0.731051

```
 C                 -0.41232000   -5.33104200   -1.85009500
 C                  2.96057400   -2.04207800   -0.40356000
 C                  3.32699100   -0.86014000    0.27058000
 C                  2.39276000   -0.16747900    1.03606700
 C                  1.07771100   -0.69951100    1.15869800
 C                  0.69522800   -1.87320200    0.44924500
 C                  1.67065000   -2.52846200   -0.34229800
 C                  0.11409600   -0.05349900    1.96300600
 C                 -1.19618800   -0.49001000    1.96788800
 C                 -1.59696200   -1.59029300    1.19115800
 C                 -0.65216000   -2.33002900    0.47385200
 C                 -0.98568100   -3.58898100   -0.25450200
 N                 -0.01276700   -4.13418600   -1.09572700
 C                  1.32529700   -3.71774900   -1.16086800
 C                  2.67006100    1.16698800    1.64950100
 N                  1.74440700    1.66260000    2.56985000
 C                  0.45870400    1.15711800    2.75317700
 O                  3.66488300    1.82008900    1.38411400
 O                 -0.34109600    1.68993800    3.50859600
 O                 -2.05877300   -4.16110500   -0.13713800
 O                  2.14243600   -4.29714000   -1.85655300
 H                  3.69661000   -2.56617800   -1.00264000
 H                 -1.93318700    0.07058600    2.52651900
 C                  2.06938200    2.96584300    3.16275300
 H                  1.36191700    3.15677300    3.96611800
 H                  1.99000200    3.74023100    2.39401900
 H                  3.09253400    2.94290000    3.53819900
 S                 -3.42811200   -1.67650900    0.95176100
 S                  5.11868100   -0.44003000    0.10922800
 C                  5.16598700    1.07338200   -0.92957700
 H                  5.32325900    0.68953600   -1.94097300
 H                  4.18821500    1.55013000   -0.85255100
 C                  6.27855500    2.00540900   -0.46230400
```

H	6.09836600	2.31544300	0.56859500
H	7.25821000	1.52227500	-0.52111900
H	6.29240700	2.89202800	-1.10511400
C	-4.04276500	-3.14856300	1.82991700
H	-3.76331300	-2.98382000	2.87515900
H	-3.51202800	-4.00641600	1.42335600
C	-5.55711900	-3.24263300	1.64331000
H	-6.05865300	-2.35224800	2.03196700
H	-5.80865700	-3.35725400	0.58517900
H	-5.92790200	-4.11852600	2.18502500
O	-3.97562500	-0.52128200	1.68914200
O	5.64722100	-0.19950700	1.45696300
O	5.69210000	-1.52658800	-0.70759000
O	-3.65557400	-1.77274700	-0.49864000
H	0.38900700	-5.57019700	-2.54507600
H	-0.58045200	-6.16611500	-1.16498700
H	-1.34280100	-5.12300900	-2.38027500
C	0.38843000	1.71156200	-2.66085100
C	0.97146000	0.28773300	-2.64013600
C	-0.25907800	-0.60982300	-2.83428800
C	-1.32712500	0.18387700	-2.08628700
H	0.45858700	2.16135100	-3.66229600
H	1.73706600	0.14702700	-3.40846000
H	1.42057000	0.10453700	-1.65825900
H	-0.52466100	-0.68926500	-3.89731500
H	-0.12671400	-1.62154000	-2.43535300
H	-2.34879000	-0.01272000	-2.42867100
H	-1.27785100	0.01821200	-1.00234200
O	-1.02601600	1.56132600	-2.36712600
C	1.02300100	2.64682800	-1.61691000
O	0.85331900	2.12787100	-0.31079700
H	-0.10314800	2.12103500	-0.08410600
C	-2.41940000	3.06924200	1.35596600
C	-2.75324900	2.85628200	-0.06341100
C	-4.04723500	2.26477700	-0.59577400
H	-4.72699900	2.02395900	0.23010700
H	-3.80763300	1.31145000	-1.08529700
C	-4.74326100	3.19636900	-1.60800200
H	-5.80597500	2.92112800	-1.68556700
H	-4.71441200	4.23193100	-1.24140300
C	-4.14668200	3.15787800	-3.02242400
H	-4.73577000	3.81991800	-3.66969700
O	-2.79592500	3.59082000	-3.09931300
H	-2.21072500	2.83534200	-2.89881700
O	-1.85423700	1.95876300	0.62801700
H	-4.25582500	2.13589000	-3.42755300
H	-2.24710700	3.50383100	-0.77567100
C	-3.31163900	2.71367800	2.51937100
H	-2.70924500	2.45245700	3.39781200
H	-3.94728200	3.56429200	2.78445200
H	-3.94904700	1.85553100	2.28416400
H	-1.68911600	3.85092400	1.56686100
C	0.52520700	4.08935200	-1.75088600
H	0.93962400	4.69466800	-0.94247400
H	0.83089400	4.52300700	-2.70825700
H	-0.56759200	4.14244100	-1.70562900
H	2.10596500	2.63376300	-1.79360600

TS, 0 water molecules
G = -3052.622169, ZPE = 0.730265

C	2.86890600	3.38829500	2.64508200
C	-1.38004000	0.97596500	2.16454100
C	-2.12808900	-0.15034300	1.78034300
C	-1.49094000	-1.29286700	1.29019300
C	-0.07082600	-1.31035600	1.23882400
C	0.68280000	-0.15726500	1.59150000
C	-0.00546300	0.99459800	2.02920800
C	0.62304500	-2.45588400	0.78579100
C	1.97538700	-2.38402700	0.52555800
C	2.69988200	-1.20064200	0.76506200
C	2.08942800	-0.11431500	1.38922300
C	2.84365000	1.08779700	1.86008000
N	2.11219000	2.16640400	2.35629100
C	0.72376000	2.26074300	2.30599300
C	-2.22004300	-2.44688800	0.69457500
N	-1.50118300	-3.62603500	0.49356700
C	-0.09823100	-3.71136400	0.47700400

O	-3.39725200	-2.39192600	0.35900100
O	0.46927300	-4.75224000	0.18747000
O	4.06515600	1.12062700	1.88002000
O	0.13414200	3.30560500	2.54665100
H	-1.88713500	1.85703000	2.53891000
H	2.46948800	-3.23526600	0.07243600
C	-2.26582100	-4.79300600	0.03606400
H	-1.65632000	-5.67894800	0.20067200
H	-2.49820600	-4.70095000	-1.02960500
H	-3.19843800	-4.84557000	0.59734400
S	4.29059000	-1.12722700	-0.16474200
S	-3.93089800	-0.00501700	2.12101400
C	-4.73166300	0.16652500	0.47693200
H	-4.80755700	1.24760400	0.33079900
H	-4.05763300	-0.27153700	-0.25817100
C	-6.08449400	-0.53683500	0.46977300
H	-5.95006100	-1.60223900	0.66689300
H	-6.75757000	-0.12030200	1.22469500
H	-6.54939900	-0.41118900	-0.51407900
C	5.64344400	-1.28807200	1.04075700
H	5.47207400	-2.25223400	1.52979500
H	5.52091900	-0.47354600	1.75225000
C	6.98208900	-1.24133000	0.30479200
H	7.05729400	-2.04967200	-0.42784100
H	7.10345800	-0.28470200	-0.21114200
H	7.79374200	-1.34983700	1.03131800
O	4.30579700	-2.36223400	-0.97509700
O	-4.36068100	-1.23611900	2.79561400
O	-4.10061100	1.29456400	2.79810600
O	4.34062700	0.17305600	-0.84764100
H	2.23280400	4.05420400	3.22413600
H	3.77086600	3.12643500	3.19638700
H	3.14666800	3.86959600	1.70206400
C	-0.22602100	-0.91237500	-3.53265000
C	-0.90186700	-2.14994000	-2.91175800
C	-2.39909100	-1.82350600	-2.97165200
C	-2.37610800	-0.31587100	-2.74455000
H	-0.01037200	-1.06700500	-4.59826100
H	-0.63765200	-3.06612000	-3.44768000
H	-0.57848000	-2.24752700	-1.87135400
H	-2.81282900	-2.04609700	-3.96218900
H	-2.99036800	-2.35636200	-2.22075300
H	-3.25680600	0.20442200	-3.13762600
H	-2.26159300	-0.07844700	-1.67734400
O	-1.22930100	0.15242600	-3.47471000
C	1.07627300	-0.52223400	-2.80741200
O	0.78166800	-0.29629400	-1.44517600
H	1.17649800	0.54817800	-1.08033400
C	0.96241300	3.09635400	-1.30109000
C	-0.42708900	2.51618000	-1.44061900
C	-1.56063900	2.92519100	-0.53589800
H	-1.15609100	3.34532500	0.38699200
H	-2.16362900	2.05044100	-0.26383700
C	-2.43995800	3.95063200	-1.28109700
H	-3.37861700	4.14595600	-0.75107200
H	-1.90060700	4.89975200	-1.37633500
C	-2.69270600	3.37569600	-2.67001700
H	-3.10642500	4.11218200	-3.36792800
O	-1.39413800	2.96350800	-3.13822400
H	-1.40612600	2.05063600	-3.50780100
O	1.30878100	2.16890600	-0.32000800
H	-3.37143300	2.51546600	-2.61996800
H	-0.41259400	1.49131600	-1.77209500
C	1.06577600	4.55394400	-0.85118100
H	2.11644900	4.78227900	-0.64402500
H	0.70662200	5.24233900	-1.62764300
H	0.50697200	4.73000300	0.07392300
H	1.52142500	3.00279800	-2.24950300
C	1.78742800	0.63467100	-3.51105500
H	2.64881300	0.96598100	-2.92257800
H	2.14338200	0.31752500	-4.49789000
H	1.11946900	1.48834100	-3.65297000
H	1.72801200	-1.41281700	-2.88986900

Starting product 1 water molecule
G = -3129.078644; ZPE = 0.755881

C	-2.36564700	5.12478400	-2.04524100

C	-3.88044800	0.71416300	-0.44504600
C	-3.65702400	-0.48887200	0.25085800
C	-2.49114000	-0.66962700	0.99029900
C	-1.57018000	0.40787000	1.08958500
C	-1.77224200	1.61114800	0.35581100
C	-2.94442400	1.73050600	-0.42916200
C	-0.39439600	0.26916000	1.85846600
C	0.59069900	1.23268800	1.79382900
C	0.43407700	2.38227900	0.99803600
C	-0.76642700	2.61771700	0.32477900
C	-1.05573900	3.88008600	-0.41795700
N	-2.17099300	3.89726200	-1.25979100
C	-3.17190600	2.91623700	-1.29392500
C	-2.08032600	-1.98851400	1.56219300
N	-1.08349700	-1.98008100	2.53568800
C	-0.16760000	-0.93990300	2.69334600
O	-2.58260200	-3.04076200	1.20146000
O	0.77863500	-1.02919700	3.46098400
O	-0.37129900	4.88593200	-0.31431800
O	-4.16079500	3.03342300	-1.99838600
H	-4.78840800	0.83872100	-1.02447100
H	1.52201700	1.07557000	2.31943000
C	-0.73958100	-3.28026900	3.12517300
H	-0.16040700	-3.10119300	4.02833100
H	-0.14633100	-3.85498500	2.40714300
H	-1.65899600	-3.81989300	3.34883000
S	2.02214300	3.28496700	0.70814600
S	-5.07511200	-1.66743700	0.21308800
C	-4.56121700	-3.02652800	-0.90930900
H	-4.99872000	-2.75169100	-1.87264200
H	-3.47241700	-3.00121800	-0.96535000
C	-5.05878600	-4.36913800	-0.38492900
H	-4.62807500	-4.57015400	0.59768600
H	-6.14944600	-4.38982200	-0.30459800
H	-4.74903300	-5.15777200	-1.07883000
C	1.92175700	4.87487300	1.59002700
H	1.77080900	4.60176800	2.63908200
H	1.05079100	5.39901900	1.20304400
C	3.22462100	5.64542000	1.37498000
H	4.08355300	5.07895000	1.74470600
H	3.37389600	5.86153400	0.31333000
H	3.17000500	6.59425500	1.91799400
O	3.04998500	2.49950000	1.41497400
O	-5.27543900	-2.14831100	1.58542600
O	-6.15861100	-0.93705000	-0.47053500
O	2.12574000	3.47758600	-0.74650300
H	-3.17673500	4.94674500	-2.74726400
H	-2.61577600	5.95758400	-1.38267000
H	-1.43840800	5.36229000	-2.56851200
C	0.28938000	-1.53214900	-2.44907000
C	-0.62939300	-0.32077400	-2.24220800
C	0.19301200	0.84563800	-2.80897100
C	1.61160100	0.45240900	-2.39664500
H	0.10848000	-2.00481900	-3.42623900
H	-1.59711600	-0.45122900	-2.73551900
H	-0.79820400	-0.18874700	-1.16858000
H	0.10978700	0.88352300	-3.90185700
H	-0.10516100	1.81994900	-2.40932400
H	2.39140000	0.83250600	-3.06511200
H	1.84323300	0.75657300	-1.37006800
O	1.63611900	-0.98899200	-2.46794300
C	0.19679400	-2.59291100	-1.34080300
O	0.43357300	-2.02109900	-0.06729600
H	1.27195900	-1.50876700	-0.10291800
C	3.27620400	-1.34685200	1.55397600
C	3.88824300	-1.62576200	0.24213600
C	5.23211400	-1.10551900	-0.21099400
H	5.45688800	-0.17325800	0.31642300
H	5.15978700	-0.85272700	-1.27757300
C	6.35731400	-2.13936200	0.00635100
H	7.32207000	-1.61911000	0.08660400
H	6.19884000	-2.65761700	0.96171900
C	6.46739700	-3.17695800	-1.11975700
H	7.24394600	-3.90838900	-0.86116000
O	5.26497300	-3.89493900	-1.34501300
H	4.74223000	-3.36970500	-1.98723000

O	2.81607700	-0.65315500	0.36368500
H	6.79309900	-2.66873600	-2.04156200
H	3.58138300	-2.55193200	-0.23668400
C	3.88850100	-0.49921700	2.63830500
H	3.10521700	0.03987200	3.18226800
H	4.42163200	-1.13482100	3.35534700
H	4.58868600	0.23524000	2.23512900
H	2.54890100	-2.08116600	1.90597300
C	1.10975800	-3.79307900	-1.61390600
H	1.05777300	-4.49308900	-0.77510300
H	0.80598600	-4.32079600	-2.52750200
H	2.15077100	-3.47967800	-1.73582100
H	-0.84503800	-2.94304200	-1.32099900
O	3.95000800	-2.27523400	-3.18834300
H	3.13398700	-1.80306200	-2.89805100
H	3.64921400	-2.84630300	-3.91531200

TS, 1 water molecule (TS-7)
G = -3129.046099; ZPE = 0.755573

C	2.87298900	3.02977600	3.10703000
C	-1.29976700	0.51191600	2.43214800
C	-2.01091500	-0.58687900	1.92029900
C	-1.34074000	-1.63248100	1.28255500
C	0.07494100	-1.56929200	1.18042300
C	0.78734800	-0.42868700	1.64173600
C	0.06684100	0.61517100	2.25992000
C	0.80000400	-2.60403700	0.54685900
C	2.12601800	-2.41595800	0.21701100
C	2.79948500	-1.22920500	0.56505300
C	2.17177400	-0.27035700	1.35886600
C	2.89078200	0.89777200	1.95182800
N	2.13824100	1.85500600	2.62866600
C	0.74663800	1.86646200	2.68590300
C	-2.03516200	-2.74423400	0.57471800
N	-1.26874800	-3.85957800	0.22611100
C	0.13149700	-3.85824100	0.12920600
O	-3.22244300	-2.71148000	0.27812000
O	0.73922400	-4.82183500	-0.30894400
O	4.10823300	1.00171100	1.92134000
O	0.12111200	2.82452800	3.12013400
H	-1.83350700	1.31133400	2.93174000
H	2.63658100	-3.17494500	-0.36387000
C	-1.99300700	-5.00579600	-0.33691000
H	-1.34104400	-5.87503600	-0.28210700
H	-2.25779700	-4.81181500	-1.38116700
H	-2.90651900	-5.16087400	0.23645800
S	4.35736400	-0.96883900	-0.38520100
S	-3.81890000	-0.52957500	2.25823100
C	-4.59202300	-0.19990600	0.62413200
H	-4.70706600	0.88735600	0.60343800
H	-3.88303100	-0.51905300	-0.13995400
C	-5.91576500	-0.94551500	0.49855500
H	-5.74572400	-2.02092200	0.57748600
H	-6.62257000	-0.64157300	1.27610900
H	-6.36078100	-0.72439000	-0.47765400
C	5.74094600	-1.24265200	0.76375400
H	5.61537300	-2.27091700	1.11717300
H	5.60413400	-0.53857500	1.58279700
C	7.06120300	-1.04185500	0.02055700
H	7.15053200	-1.73748900	-0.81848700
H	7.13646500	-0.01899700	-0.35902700
H	7.89140100	-1.21824100	0.71194200
O	4.39028700	-2.08258600	-1.35537400
O	-4.22743500	-1.83392100	2.79398600
O	-4.03160400	0.68603600	3.06704500
O	4.35117200	0.41171000	-0.88784400
H	2.18064300	3.65371700	3.66721400
H	3.70416000	2.70483100	3.73430800
H	3.27118300	3.58357600	2.25253400
C	-0.15476800	-0.86755300	-3.49677000
C	-0.86376500	-2.13689000	-2.96592900
C	-2.36448600	-1.81981800	-3.09491700
C	-2.37224100	-0.30175900	-2.94073100
H	0.28048800	-1.02477100	-4.49163200
H	-0.57151000	-3.02960600	-3.52630600
H	-0.59852400	-2.28240900	-1.91571900
H	-2.74039400	-2.09293000	-4.08758700

H	-2.97542800	-2.32651900	-2.34164400
H	-3.23778100	0.19524900	-3.39068500
H	-2.27353700	-0.00715200	-1.88875400
O	-1.20831100	0.12106800	-3.67004200
C	0.91942400	-0.34109600	-2.53434200
O	0.28429400	-0.10516500	-1.30019500
H	0.70726600	0.66845400	-0.81545600
C	0.80203500	3.27340300	-0.72598200
C	-0.61265900	2.86741300	-1.01523600
C	-1.68792200	2.90163100	0.04393800
H	-1.21158500	2.90483700	1.02601200
H	-2.29701200	1.99249100	-0.02300900
C	-2.57990700	4.14432300	-0.15854100
H	-3.51582100	4.06753500	0.40516600
H	-2.04995900	5.04053800	0.18322300
C	-2.82963500	4.26231100	-1.65555700
H	-3.28884500	5.21839700	-1.93500100
O	-1.52338100	4.16854400	-2.23794600
H	-1.53926600	3.67745400	-3.11077500
O	1.07081700	2.09204600	-0.03851500
H	-3.46969800	3.44498800	-2.01694000
H	-0.68663000	2.06965000	-1.74410400
C	1.00106300	4.54017700	0.10332200
H	2.05948000	4.62615500	0.37115100
H	0.70391300	5.43497400	-0.45798000
H	0.42989800	4.49501200	1.03692500
H	1.36190100	3.39079500	-1.67547000
C	1.63742100	0.89195800	-3.09242500
H	2.32611600	1.29289800	-2.34342900
H	2.20495700	0.63695100	-3.99577900
H	0.92082800	1.67995000	-3.34669300
H	1.66955600	-1.15145300	-2.43779900
O	-1.40323300	2.70036300	-4.43982800
H	-1.26869700	1.74574800	-4.20670300
H	-0.62975400	2.94265000	-4.97020200

Starting product, 2 water molecules
G = -3205.497897; ZPE = 0.783249

C	4.29460800	3.75472000	-0.26832600
C	-0.09212700	2.49100900	1.57981200
C	-1.01873200	1.52801900	2.01970100
C	-0.64376000	0.18966300	2.12920100
C	0.66702000	-0.18902800	1.72378500
C	1.57313300	0.77192300	1.19782200
C	1.17351600	2.12727200	1.16944600
C	1.08939800	-1.53740100	1.79918700
C	2.27206000	-1.92828500	1.20497500
C	3.12361500	-0.98925900	0.59344100
C	2.82981400	0.37167200	0.66280100
C	3.78986900	1.43512800	0.24229600
N	3.32580400	2.75234200	0.19525600
C	2.08600000	3.18606900	0.67199200
C	-1.56835800	-0.88772100	2.58555000
N	-0.98987100	-2.11242800	2.94487300
C	0.25552400	-2.55755700	2.48032900
O	-2.77867500	-0.74909000	2.66210600
O	0.61307500	-3.71494400	2.62657900
O	4.96108300	1.20788200	-0.01669100
O	1.77945800	4.36799900	0.68430200
H	-0.38507700	3.53358000	1.53462500
H	2.52994300	-2.98085500	1.18389700
C	-1.88172000	-3.10375000	3.55926700
H	-1.26482300	-3.85801300	4.04338700
H	-2.50914000	-3.58107800	2.79910400
H	-2.52418100	-2.59671300	4.27828800
S	4.42318500	-1.75686700	-0.47668100
S	-2.66547600	2.23590100	2.46050300
C	-3.80357800	1.51536500	1.21369000
H	-3.85363100	2.28031500	0.43474900
H	-3.32250700	0.62015100	0.82124400
C	-5.16057200	1.19863200	1.82991800
H	-5.04529700	0.47907700	2.64237400
H	-5.64537600	2.09833200	2.21970400
H	-5.80643700	0.76584500	1.05801700
C	5.98603000	-1.59362900	0.44272800
H	5.81990300	-2.12561400	1.38471400
H	6.13069000	-0.53017600	0.62607800

C	7.11854400	-2.21067800	-0.37790200
H	6.92913100	-3.26931700	-0.57580200
H	7.23062300	-1.68783600	-1.33184700
H	8.05578100	-2.12088600	0.18047300
O	4.09649600	-3.19457000	-0.48593300
O	-3.00587900	1.81689600	3.82526900
O	-2.55960800	3.67422800	2.15012800
O	4.47494900	-1.00716000	-1.73592100
H	3.78704100	4.71535800	-0.31048800
H	5.14073800	3.80323100	0.42157900
H	4.66640800	3.46347900	-1.25243400
C	-3.14497500	-2.95872800	-0.92881500
C	-4.21619100	-1.90089300	-0.63867200
C	-5.19323200	-2.09383000	-1.80632800
C	-4.25128200	-2.45689900	-2.96207900
H	-3.45301600	-3.93416500	-0.51734500
H	-4.67868000	-2.03926800	0.34328700
H	-3.76253100	-0.90569400	-0.66838100
H	-5.88078100	-2.92161900	-1.59694400
H	-5.79080200	-1.20303400	-2.02209400
H	-4.69575600	-3.16936400	-3.66668600
H	-3.93260300	-1.57015500	-3.52452600
O	-3.09095500	-3.06921500	-2.36459100
C	-1.75211600	-2.64563100	-0.37103400
O	-1.38632500	-1.32440100	-0.78760200
H	-0.39582800	-1.24774800	-0.83856800
C	0.80536700	2.68989000	-2.85806100
C	-0.30207000	1.74692400	-2.61306200
C	-1.51728300	2.02845000	-1.76172400
H	-1.26509700	2.80254400	-1.02984400
H	-1.73641300	1.11994600	-1.18369300
C	-2.76294300	2.44811900	-2.56587000
H	-3.46397900	2.94293900	-1.87887000
H	-2.49133700	3.19045700	-3.32796800
C	-3.52420800	1.30196500	-3.24569000
H	-4.47502700	1.69475800	-3.62821800
O	-2.85996600	0.72255200	-4.35559700
H	-2.23056000	0.03725600	-4.04696500
O	0.95518300	1.62154700	-1.89526700
H	-3.77608900	0.54285200	-2.48556600
H	-0.43715000	0.97617400	-3.36858500
C	0.88204200	4.09695000	-2.32369200
H	1.92113100	4.35612100	-2.09122000
H	0.51647800	4.80441500	-3.07739400
H	0.29097800	4.21836500	-1.41321100
H	1.38200300	2.52564300	-3.77192100
C	-0.71371000	-3.68200000	-0.80212300
H	0.27319500	-3.43286800	-0.40085600
H	-0.99671900	-4.67831300	-0.44489600
H	-0.64282700	-3.71461800	-1.89283200
H	-1.84227400	-2.65625800	0.72734900
O	-1.13276900	-1.31322600	-3.47574400
H	-1.40160500	-1.29758900	-2.52001000
H	-1.52246400	-2.15360700	-3.76806200
O	1.13628400	-1.13967600	-1.70171700
H	1.34953400	-0.18387900	-1.75367800
H	0.71262600	-1.32559200	-2.56194100

TS, 2 water molecules (TS-2)

G = -3205.457889; ZPE = 0.780190

C	1.16660000	5.39867200	-2.34510900
C	-2.65419100	2.66598700	-0.87171400
C	-3.22776400	1.66480900	-0.06509000
C	-2.44937000	0.98408000	0.86860400
C	-1.06332500	1.29851800	0.96113000
C	-0.48915100	2.32813100	0.16334100
C	-1.32120800	3.00099300	-0.76148600
C	-0.20989400	0.54427000	1.79595200
C	1.14524200	0.80746500	1.83474500
C	1.70390500	1.85949100	1.08725900
C	0.89744200	2.63431700	0.24969100
C	1.41462200	3.78821600	-0.54389300
N	0.58105500	4.33376200	-1.51986300
C	-0.77809700	4.02958500	-1.68148600
C	-2.99478200	-0.09075300	1.75028700
N	-2.11708400	-0.71113800	2.64778200
C	-0.73357400	-0.56830000	2.62956900

O	-4.16810400	-0.41987300	1.75096200
O	0.00235500	-1.29710100	3.28184200
O	2.52583100	4.27013400	-0.37483600
O	-1.46061400	4.58802300	-2.52501600
H	-3.26659500	3.17036700	-1.61043600
H	1.78260800	0.18934800	2.45503200
C	-2.70844300	-1.76142200	3.48164000
H	-1.94121300	-2.12352600	4.16212800
H	-3.07244200	-2.57564300	2.84777000
H	-3.55710100	-1.34979400	4.03001000
S	3.54217400	1.94785800	1.23123300
S	-5.02445400	1.39824000	-0.41704700
C	-5.11399700	-0.35798900	-0.94322000
H	-5.13265800	-0.28873000	-2.03438000
H	-4.18675200	-0.84192800	-0.62573700
C	-6.35396200	-1.03991900	-0.37690200
H	-6.32979600	-1.02248700	0.71335400
H	-7.27193200	-0.55261800	-0.71862500
H	-6.37284700	-2.08159400	-0.71718800
C	3.92122600	3.49872500	2.11085100
H	3.43377300	3.38868600	3.08421400
H	3.47070600	4.30995700	1.54331200
C	5.43826000	3.63844400	2.23374800
H	5.86901000	2.79251300	2.77654600
H	5.89985400	3.69661700	1.24406600
H	5.66847100	4.55881200	2.77986200
O	3.90801100	0.86961500	2.17335400
O	-5.80382800	1.66722000	0.79723700
O	-5.29256100	2.20740300	-1.62323700
O	4.12432700	1.93402200	-0.12010800
H	0.45347400	5.64230000	-3.12904300
H	1.36978400	6.27959700	-1.73033800
H	2.10893600	5.04655800	-2.76799900
C	4.19359300	-1.70704700	-1.52652400
C	4.63531800	-1.34300700	-0.09077200
C	4.93595700	-2.69961300	0.56609400
C	3.94513100	-3.60662600	-0.15569200
H	4.99096100	-1.52843400	-2.25788800
H	5.49522900	-0.66723800	-0.08780100
H	3.80363100	-0.85269600	0.42303000
H	5.96082200	-3.02357100	0.35164900
H	4.79439400	-2.69353600	1.65113400
H	4.22125900	-4.66624800	-0.15999400
H	2.93205200	-3.49167300	0.25222000
O	3.97459900	-3.14793600	-1.51807400
C	2.92524600	-0.96345300	-1.95749600
O	1.89693800	-1.36695300	-1.07414100
H	1.09055000	-0.79976300	-1.25037000
C	-1.99375600	-2.82735700	-0.92492700
C	-0.62273500	-3.24506900	-0.47724700
C	-0.34726900	-3.63293700	0.95542000
H	-1.08651900	-3.14123700	1.59053100
H	0.64181500	-3.26100000	1.24732700
C	-0.40130000	-5.16583000	1.10638700
H	0.02152300	-5.49391500	2.06228900
H	-1.44060600	-5.50850000	1.06163000
C	0.36582900	-5.74438500	-0.07328400
H	0.19538100	-6.82094400	-0.20036300
O	-0.13295200	-5.04145700	-1.21924500
H	0.62105400	-4.87768400	-1.85507400
O	-1.90096100	-1.59763200	-0.27410400
H	1.44524400	-5.56832100	0.03221500
H	0.18196200	-2.75304000	-1.01164800
C	-3.17065500	-3.68385000	-0.46767800
H	-4.10482300	-3.18417600	-0.74533700
H	-3.14730300	-4.67704100	-0.93304600
H	-3.17040000	-3.79554900	0.62203600
H	-2.01888500	-2.75731400	-2.03166200
C	2.54594700	-1.19586500	-3.42333200
H	1.65716500	-0.60475700	-3.66828400
H	3.35463600	-0.88665100	-4.09717900
H	2.31282900	-2.24790800	-3.60791700
H	3.14742800	0.11201800	-1.82765900
O	1.98124000	-4.52768900	-2.70835700
H	2.63807400	-3.91273600	-2.29127700
H	2.51078900	-5.22691000	-3.11746300

O	-0.33983700	-0.02662900	-1.61913200
H	-1.00983400	-0.57679600	-1.06973100
H	-0.61563700	-0.16761200	-2.53667400

Starting product, 2 water molecules, S,S-configuration
G = -3205.512054; ZPE = 0.780991

C	-3.49376400	0.54596200	-3.89703400
C	1.26480200	0.28981900	-2.61692800
C	2.32647200	-0.35183800	-1.94101300
C	2.07417300	-1.44697000	-1.12641200
C	0.72246500	-1.79658800	-0.85775600
C	-0.33860500	-1.19359700	-1.57720100
C	-0.03459700	-0.15240200	-2.48712200
C	0.41150600	-2.65617100	0.21705300
C	-0.90537200	-2.87844000	0.57128500
C	-1.95716500	-2.34398600	-0.19574100
C	-1.68601000	-1.55167900	-1.31046600
C	-2.72196000	-1.09501400	-2.28046100
N	-2.39717400	-0.00525300	-3.09189700
C	-1.11851600	0.56318800	-3.20438200
C	3.14970100	-2.23717000	-0.46741500
N	2.78227100	-3.12001400	0.54899400
C	1.49098200	-3.22348800	1.06424400
O	4.31902900	-2.15101600	-0.81076700
O	1.26062600	-3.78738300	2.12463400
O	-3.81151000	-1.63497900	-2.39659000
O	-0.92052000	1.56674800	-3.87077300
H	1.46172200	1.16672900	-3.22254700
H	-1.12317800	-3.44609800	1.46830100
C	3.87836300	-3.78300700	1.26311600
H	3.44965600	-4.54816700	1.90662000
H	4.42115300	-3.05358200	1.87158800
H	4.56409400	-4.21753200	0.53571600
S	-3.61815800	-2.51632200	0.57538500
S	3.93790400	0.53192400	-2.15103200
C	4.74776200	0.65876900	-0.49862900
H	4.63028600	1.71940600	-0.26254100
H	4.15656000	0.08114300	0.21398300
C	6.20968900	0.23725800	-0.56610800
H	6.29675000	-0.80659400	-0.86713600
H	6.77297200	0.85490500	-1.27109500
H	6.65198400	0.36037200	0.42900300
C	-4.46573500	-3.82984100	-0.35231000
H	-3.83319600	-4.71589800	-0.23899900
H	-4.47872100	-3.50342500	-1.39191800
C	-5.86587700	-4.03791600	0.22367900
H	-5.81894700	-4.32557800	1.27773500
H	-6.45845800	-3.12326700	0.13319200
H	-6.36808700	-4.83439600	-0.33443900
O	-3.35823800	-3.03520100	1.93231100
O	4.77781300	-0.18043400	-3.11980100
O	3.49815600	1.91901600	-2.45431600
O	-4.32407100	-1.23636400	0.41975900
H	-3.12013900	1.43153200	-4.40579200
H	-3.83408000	-0.19882400	-4.62014000
H	-4.32767700	0.80049100	-3.23906800
C	-2.64081154	2.31813741	-0.00683034
C	-1.38968355	2.20520033	0.75316540
C	-0.05852534	2.81914604	0.40622589
H	-0.01374701	3.07272558	-0.65877297
H	0.69120779	2.04053632	0.58812766
C	0.27555849	4.05255849	1.26844603
H	1.30430053	4.36166138	1.03652697
H	-0.37839445	4.89416127	1.00631113
C	0.14355658	3.82292246	2.78206971
H	0.73172849	4.58954540	3.31211519
O	-1.21408548	3.91426025	3.18585178
H	-1.47543705	3.07696447	3.62959403
O	-1.85202095	1.09641401	-0.08924364
H	0.57272886	2.84199339	3.03757446
H	-1.50311661	1.98307466	1.80689388
C	-2.79951463	3.09298218	-1.28824243
H	-3.52772637	2.60240161	-1.94419318
H	-3.16439014	4.10327207	-1.06899413
H	-1.85312548	3.17146494	-1.82835817
H	-3.56438080	2.19080587	0.56277600
O	-1.95807425	1.42849604	4.22092340

H	-1.19994737	0.90700973	4.55720313
H	-2.30648642	0.84069771	3.52342050
O	-2.08029141	-0.46486698	2.10022320
H	-2.10262614	-0.06173975	1.19368243
H	-2.42530185	-1.36422892	2.00944613
C	1.39408049	0.06046985	4.55881659
C	2.31977828	-0.54994601	5.61564154
C	1.43195834	-0.55045206	6.86886283
C	0.06149775	-0.89528394	6.27882746
H	1.43752718	1.15928185	4.58856719
H	3.24485064	0.01977487	5.73756602
H	2.58701701	-1.57730550	5.33396518
H	1.41220290	0.44856151	7.31943866
H	1.75411280	-1.26349935	7.63365109
H	-0.77955374	-0.46604730	6.83288926
H	-0.08402121	-1.98258645	6.21126946
O	0.05806973	-0.34217879	4.95062631
C	1.62308444	-0.39014383	3.11570153
O	0.66797784	0.19557807	2.24854374
H	-0.22218645	-0.17401625	2.42579754
C	2.99948466	0.02708784	2.60929024
H	3.10700871	-0.26712202	1.56143697
H	3.80209377	-0.43841721	3.19105211
H	3.10787958	1.11636426	2.66787738
H	1.53834205	-1.49122551	3.09873211

TS, 2 water molecules, S,S-configuration (TS-8)
G = -3205.463928; ZPE = 0.781514

C	-3.49376400	0.54596200	-3.89703400
C	1.26480200	0.28981900	-2.61692800
C	2.32647200	-0.35183800	-1.94101300
C	2.07417300	-1.44697000	-1.12641200
C	0.72246500	-1.79658800	-0.85775600
C	-0.33860500	-1.19359700	-1.57720100
C	-0.03459700	-0.15240200	-2.48712200
C	0.41150600	-2.65617100	0.21705300
C	-0.90537200	-2.87844000	0.57128500
C	-1.95716500	-2.34398600	-0.19574100
C	-1.68601000	-1.55167900	-1.31046600
C	-2.72196000	-1.09501400	-2.28046100
N	-2.39717400	-0.00525300	-3.09189700
C	-1.11851600	0.56318800	-3.20438200
C	3.14970100	-2.23717000	-0.46741500
N	2.78227100	-3.12001400	0.54899400
C	1.49098200	-3.22348800	1.06424400
O	4.31902900	-2.15101600	-0.81076700
O	1.26062600	-3.78738300	2.12463400
O	-3.81151000	-1.63497900	-2.39659000
O	-0.92052000	1.56674800	-3.87077300
H	1.46172200	1.16672900	-3.22254700
H	-1.12317800	-3.44609800	1.46830100
C	3.87836300	-3.78300700	1.26311600
H	3.44965600	-4.54816700	1.90662000
H	4.42115300	-3.05358200	1.87158800
H	4.56409400	-4.21753200	0.53571600
S	-3.61815800	-2.51632200	0.57538500
S	3.93790400	0.53192400	-2.15103200
C	4.74776200	0.65876900	-0.49862900
H	4.63028600	1.71940600	-0.26254100
H	4.15656000	0.08114300	0.21398300
C	6.20968900	0.23725800	-0.56610800
H	6.29675000	-0.80659400	-0.86713600
H	6.77297200	0.85490500	-1.27109500
H	6.65198400	0.36037200	0.42900300
C	-4.46573500	-3.82984100	-0.35231000
H	-3.83319600	-4.71589800	-0.23899900
H	-4.47872100	-3.50342500	-1.39191800
C	-5.86587700	-4.03791600	0.22367900
H	-5.81894700	-4.32557800	1.27773500
H	-6.45845800	-3.12326700	0.13319200
H	-6.36808700	-4.83439600	-0.33443900
O	-3.35823800	-3.03520100	1.93231100
O	4.77781300	-0.18043400	-3.11980100
O	3.49815600	1.91901600	-2.45431600
O	-4.32407100	-1.23636400	0.41975900
H	-3.12013900	1.43153200	-4.40579200
H	-3.83408000	-0.19882400	-4.62014000

H	-4.32767700	0.80049100	-3.23906800
C	2.34606800	0.27690100	2.49825200
C	0.89446600	0.61103400	2.66041500
C	-0.19341100	-0.38917700	2.95574800
H	0.21787600	-1.38722200	3.11617100
H	-0.84719100	-0.42005100	2.07879500
C	-0.99625800	0.08645000	4.19501200
H	-2.03545400	-0.25714300	4.15988300
H	-0.53936600	-0.31386900	5.10724100
C	-0.90431500	1.60534800	4.24337100
H	-1.31062900	2.02583100	5.17112800
O	0.50500700	1.86708200	4.17325100
H	0.69859300	2.68755200	3.61942200
O	2.14555300	-0.21065800	1.19520400
H	-1.41777100	2.05755100	3.38353300
H	0.56924000	1.40980700	2.01883600
C	2.94141000	-0.74434800	3.45861900
H	3.96479500	-0.97602200	3.14545400
H	2.96440700	-0.35698600	4.48480900
H	2.37185500	-1.67897000	3.43873900
H	2.95444400	1.20109600	2.54079900
O	1.12023300	3.71531900	2.35566200
H	0.35026200	4.07361500	1.86179800
H	1.57258300	3.19882200	1.64710600
O	1.69952300	2.05688900	0.14524900
H	1.94220000	1.07212000	0.44680700
H	2.24923800	2.26235400	-0.62400500
C	-2.10869100	3.88089600	0.55908100
C	-3.08296600	4.90810300	-0.03287400
C	-2.45397000	6.24707600	0.37847800
C	-0.96218300	5.93731800	0.24080800
H	-2.38159200	3.63485200	1.59686000
H	-4.10322500	4.77333800	0.33599600
H	-3.10455300	4.81731400	-1.12705100
H	-2.69945800	6.47949100	1.42135100
H	-2.77152200	7.08925800	-0.24360600
H	-0.32104200	6.52374600	0.90654700
H	-0.61722900	6.08394000	-0.79268500
O	-0.82461500	4.55032000	0.59226200
C	-1.96682100	2.56905000	-0.21774100
O	-1.01667100	1.72099000	0.40025600
H	-0.10052800	1.96523200	0.12293100
C	-3.28991800	1.80901600	-0.26845600
H	-3.14449900	0.86315500	-0.79736500
H	-4.07299600	2.38248900	-0.77589600
H	-3.62821400	1.57768100	0.74880100
H	-1.64739900	2.82707100	-1.24157100

5. NMR Spectra

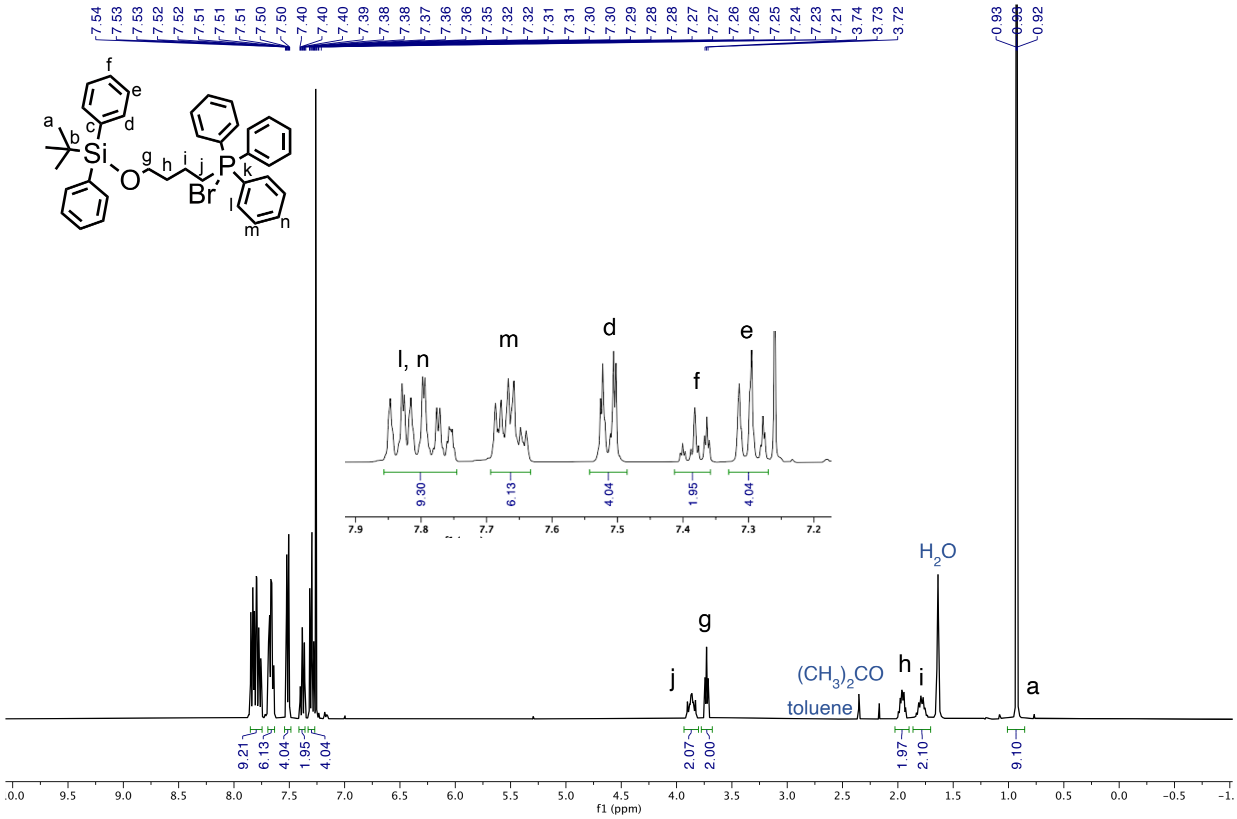

Figure S23. 400 MHz ^{1}H NMR spectrum of **28** in $CDCl_3$.

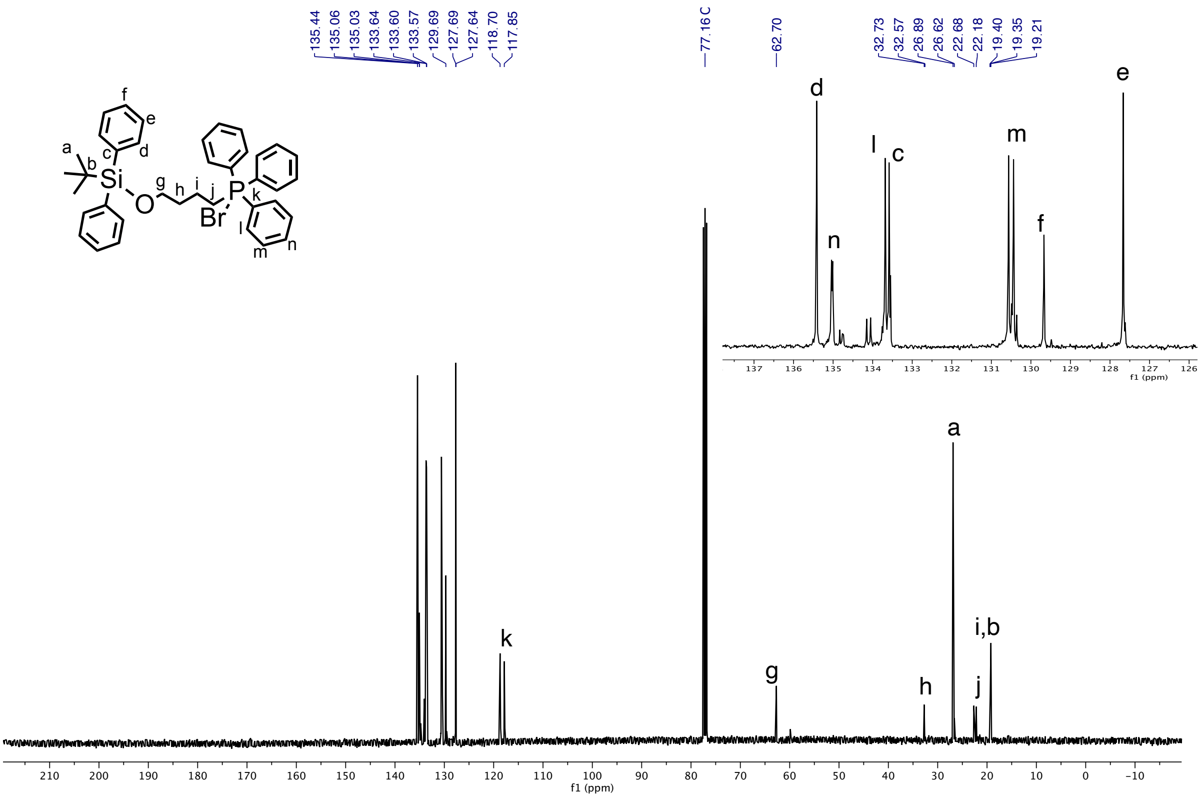

Figure S24. 101 MHz ^{13}C NMR spectrum of **28** in $CDCl_3$.

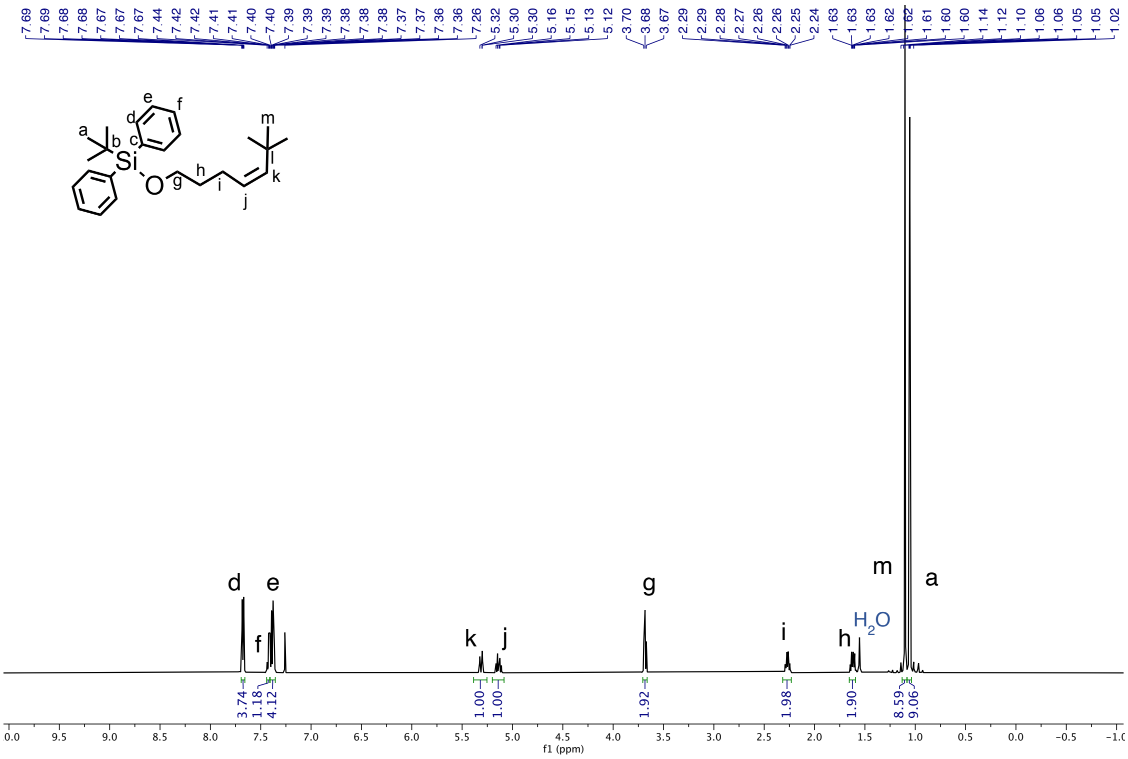

Figure S25. 500 MHz ^{1}H NMR spectrum of **30** in $CDCl_3$.

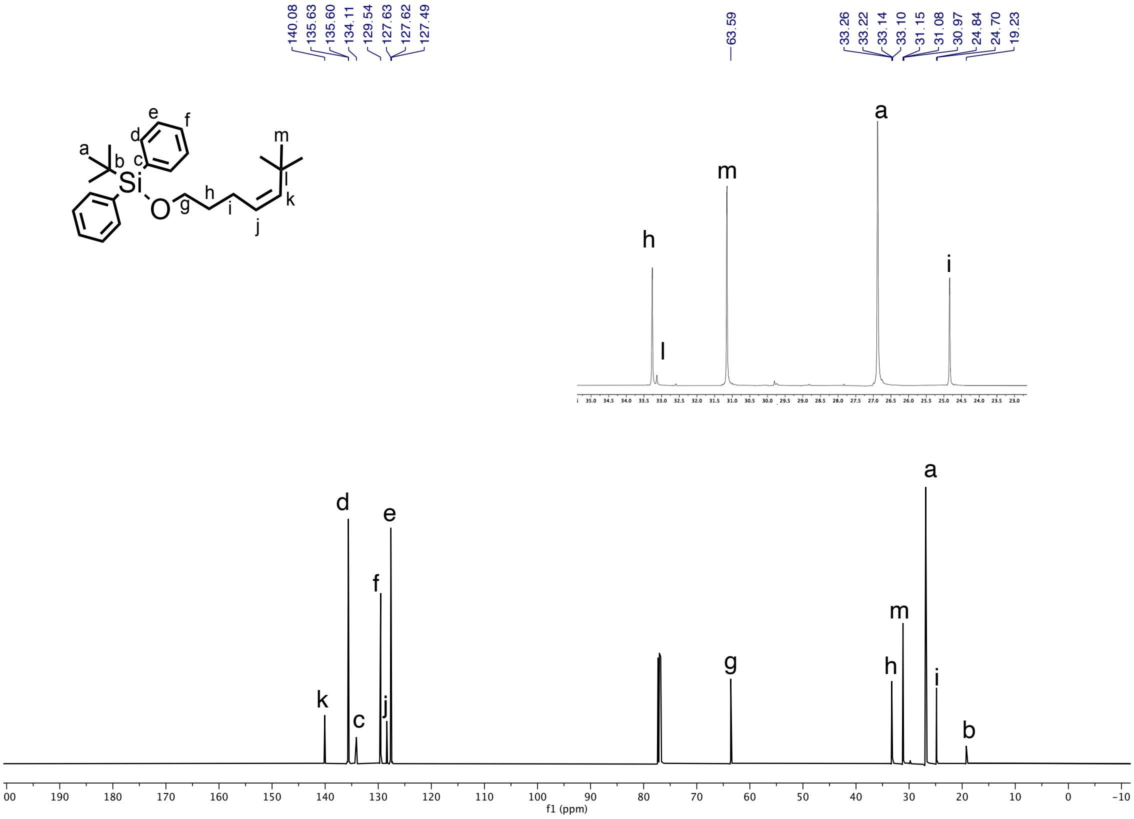

Figure S26. 126 MHz ^{13}C NMR spectrum of **30** in $CDCl_3$.

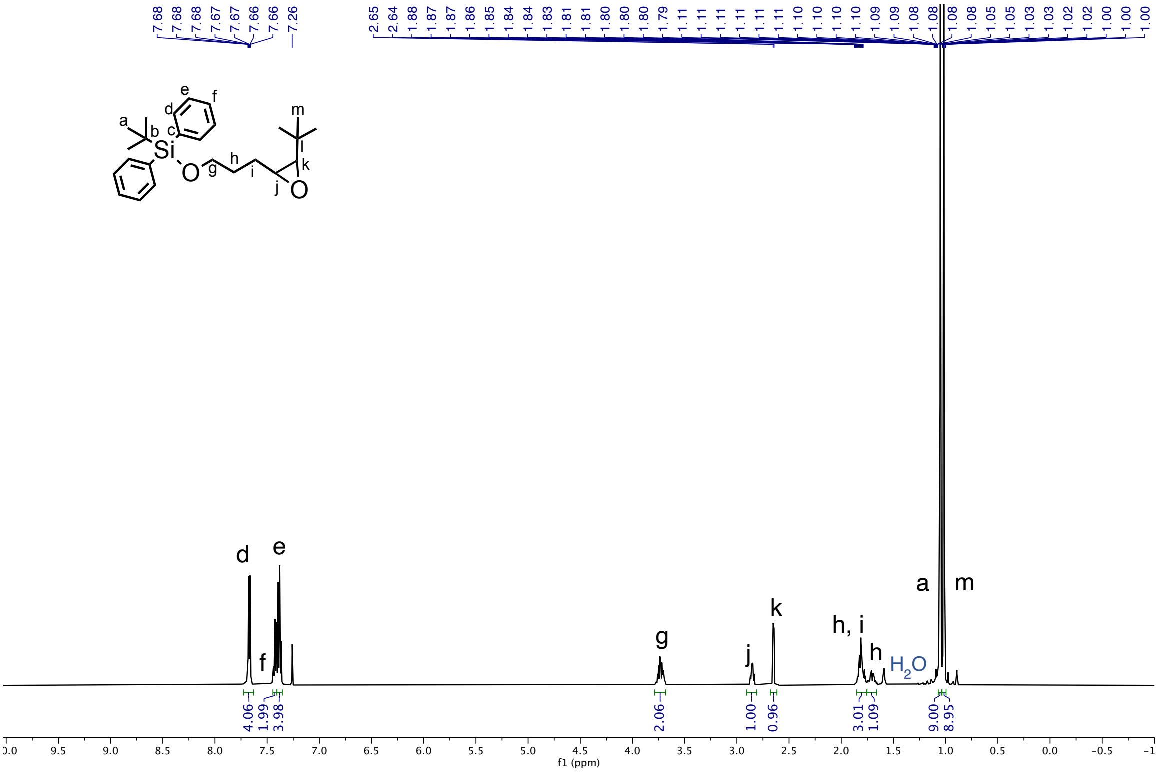

Figure S27. 500 MHz ^1H NMR spectrum of **31** in $CDCl_3$.

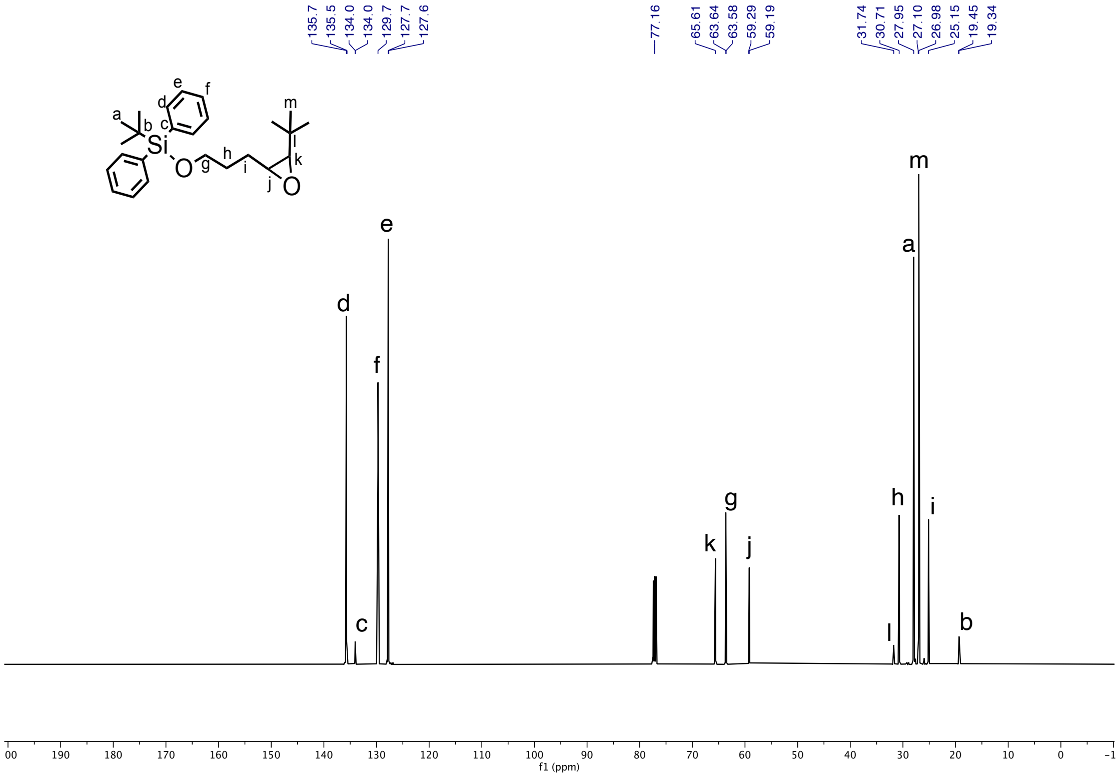

Figure S28. 126 MHz ^{13}C NMR spectrum of **31** in $CDCl_3$.

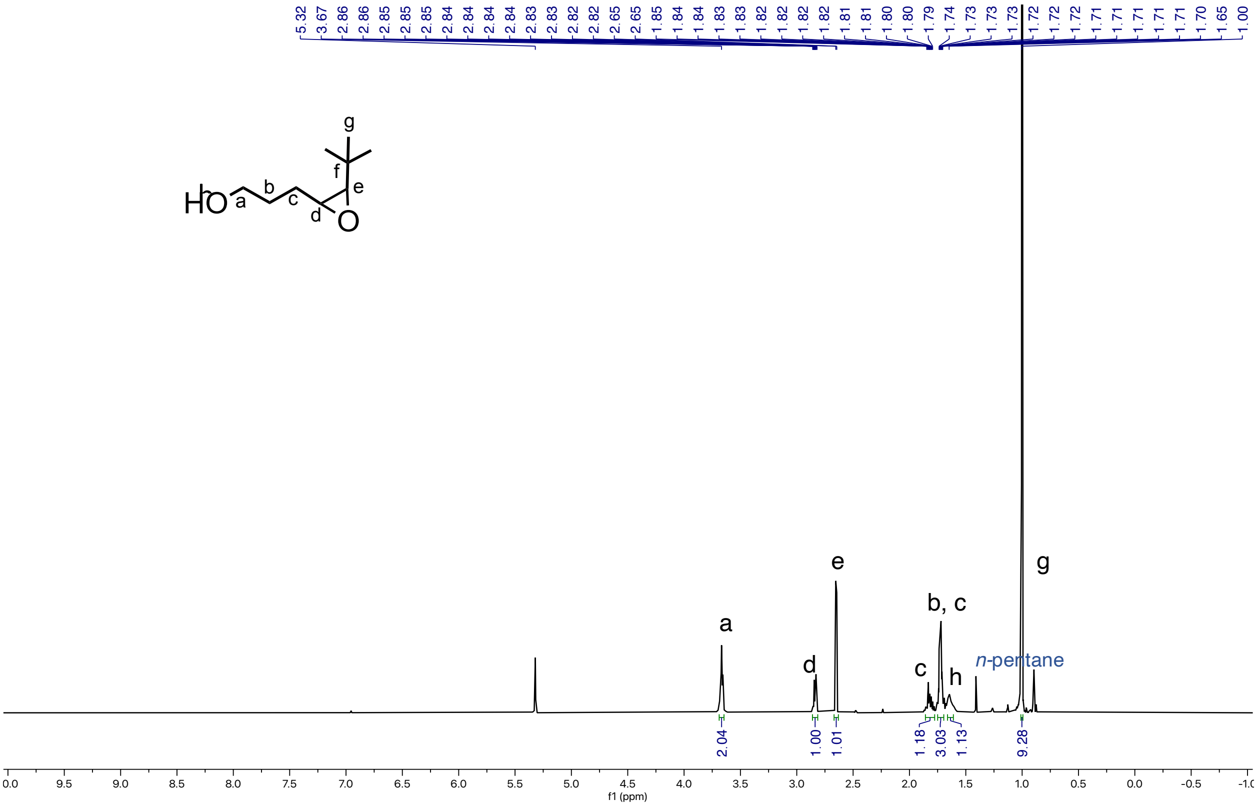

Figure S29. 500 MHz ^{1}H NMR spectrum of **20** in CD_2Cl_2.

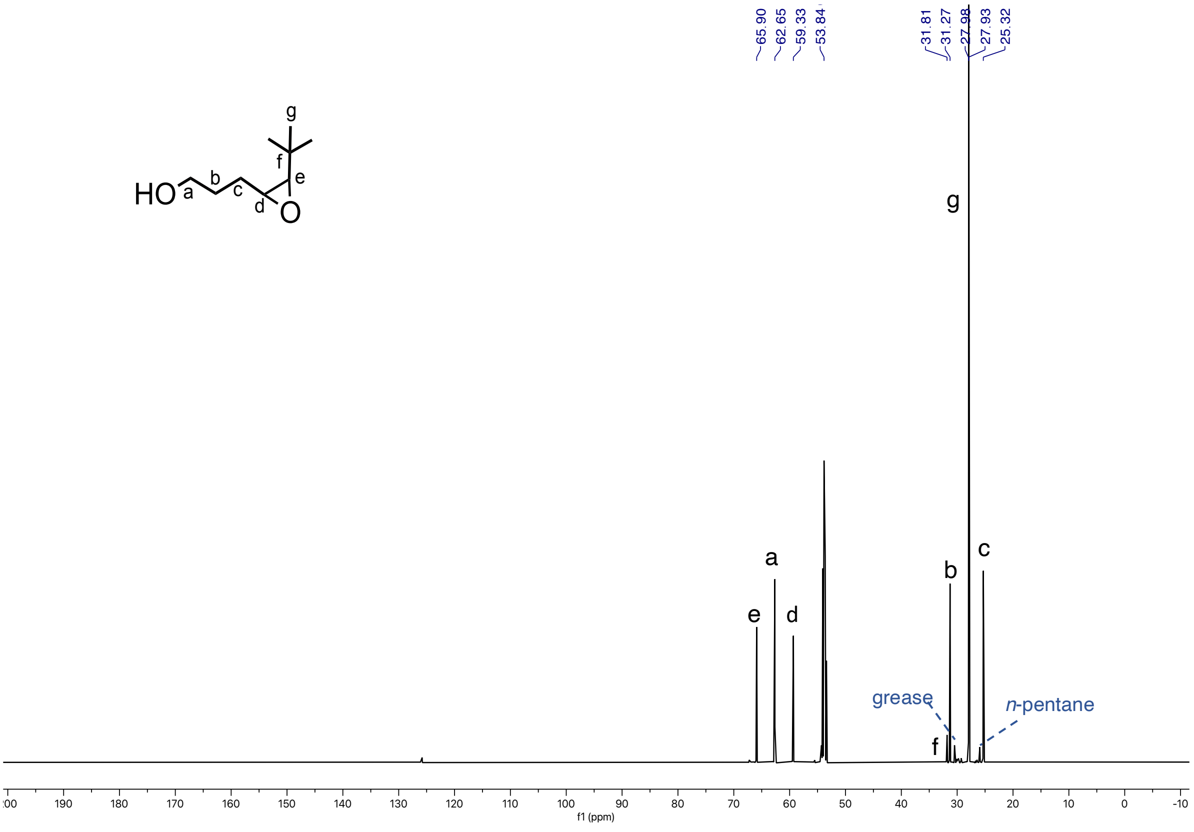

Figure S30. 126 MHz ^{13}C NMR spectrum of **20** in CD_2Cl_2.

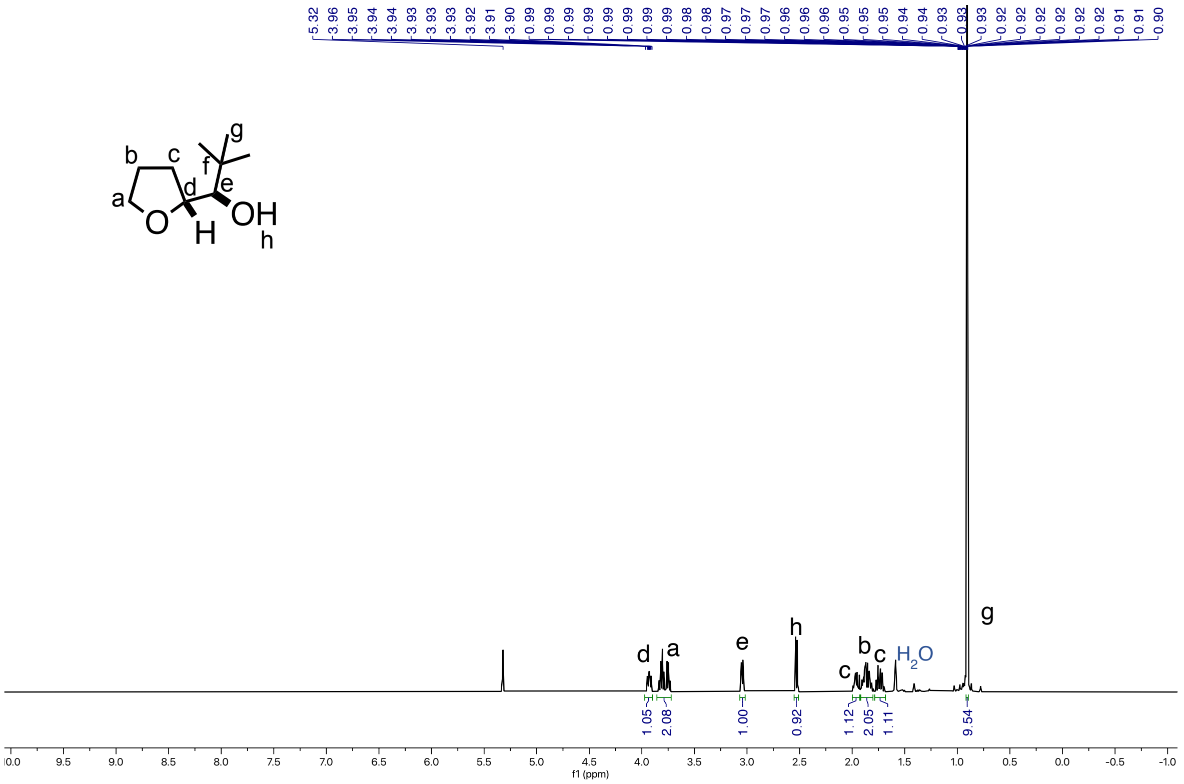

Figure S31. 500 MHz ^{1}H NMR spectrum of **23** in CD_2Cl_2.

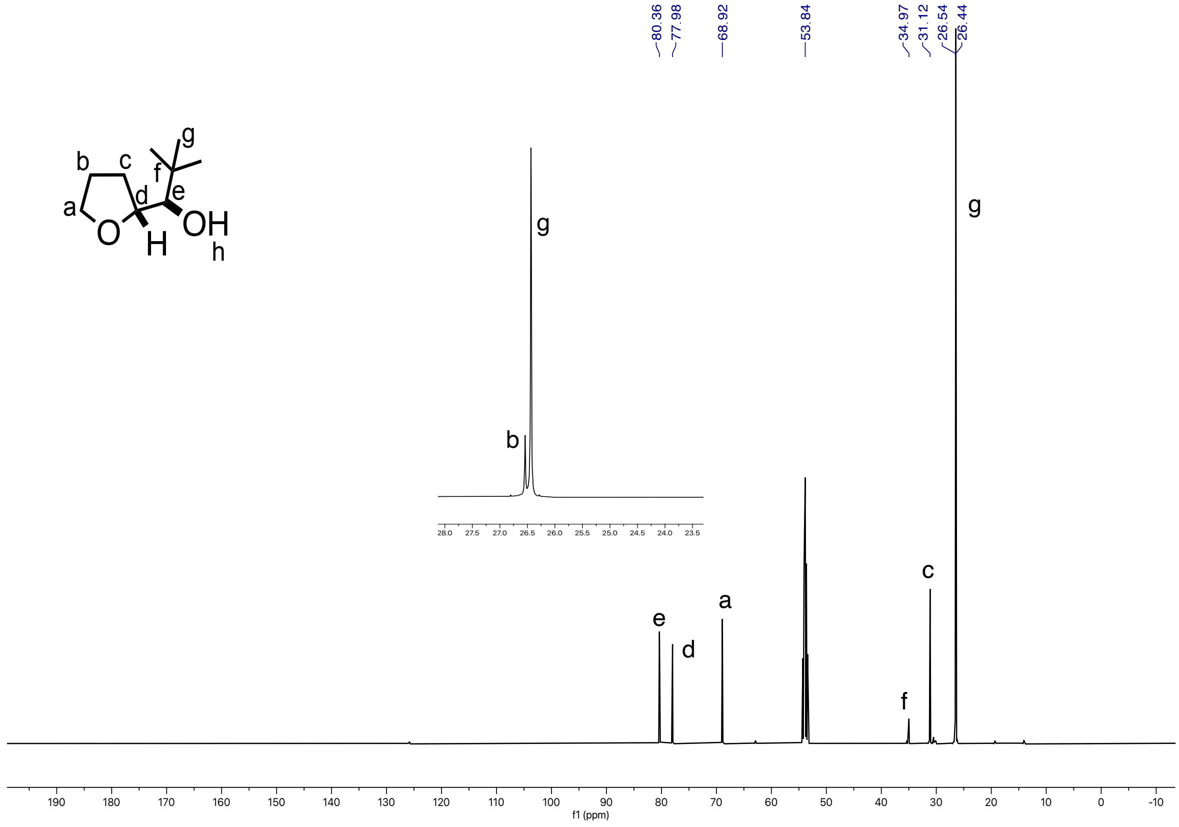

Figure S32. 126 MHz ^{13}C NMR spectrum of **23** in CD_2Cl_2.

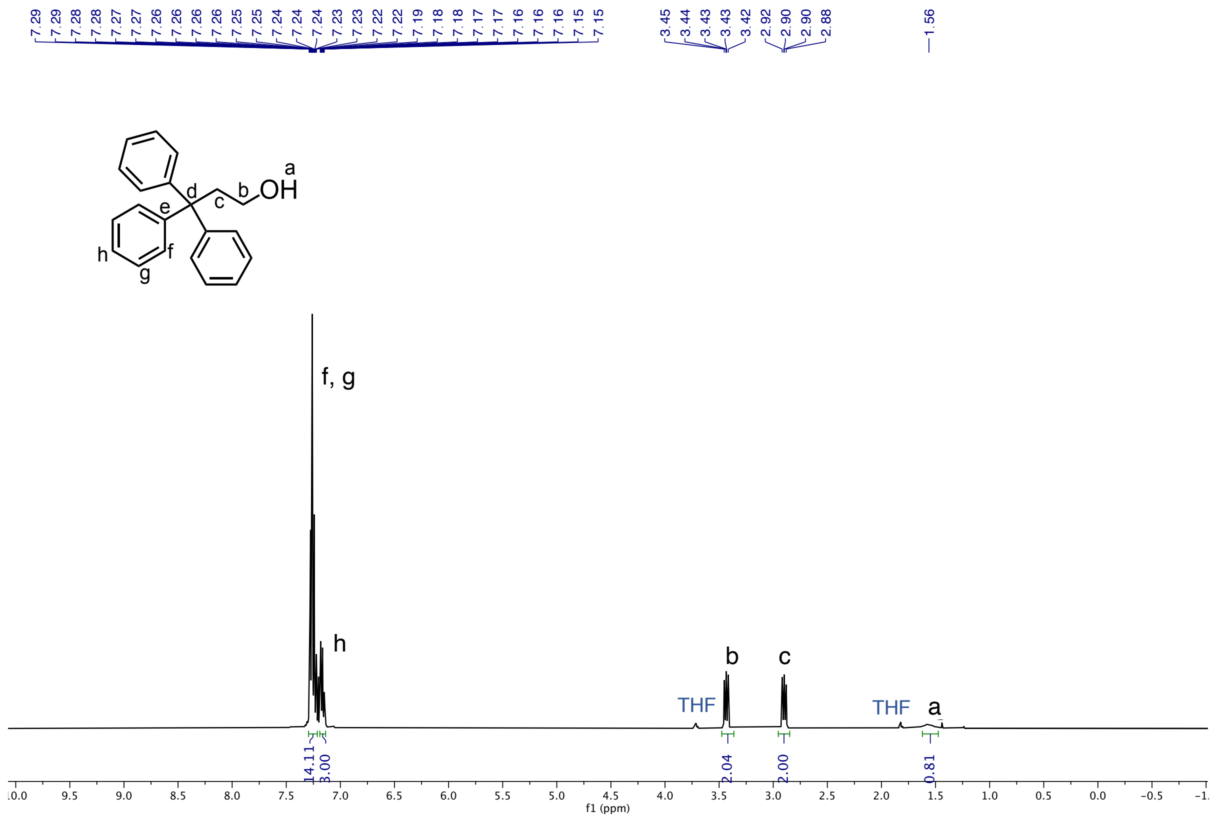

Figure S33. 400 MHz ^{1}H NMR spectrum of **39** in $CDCl_3$.

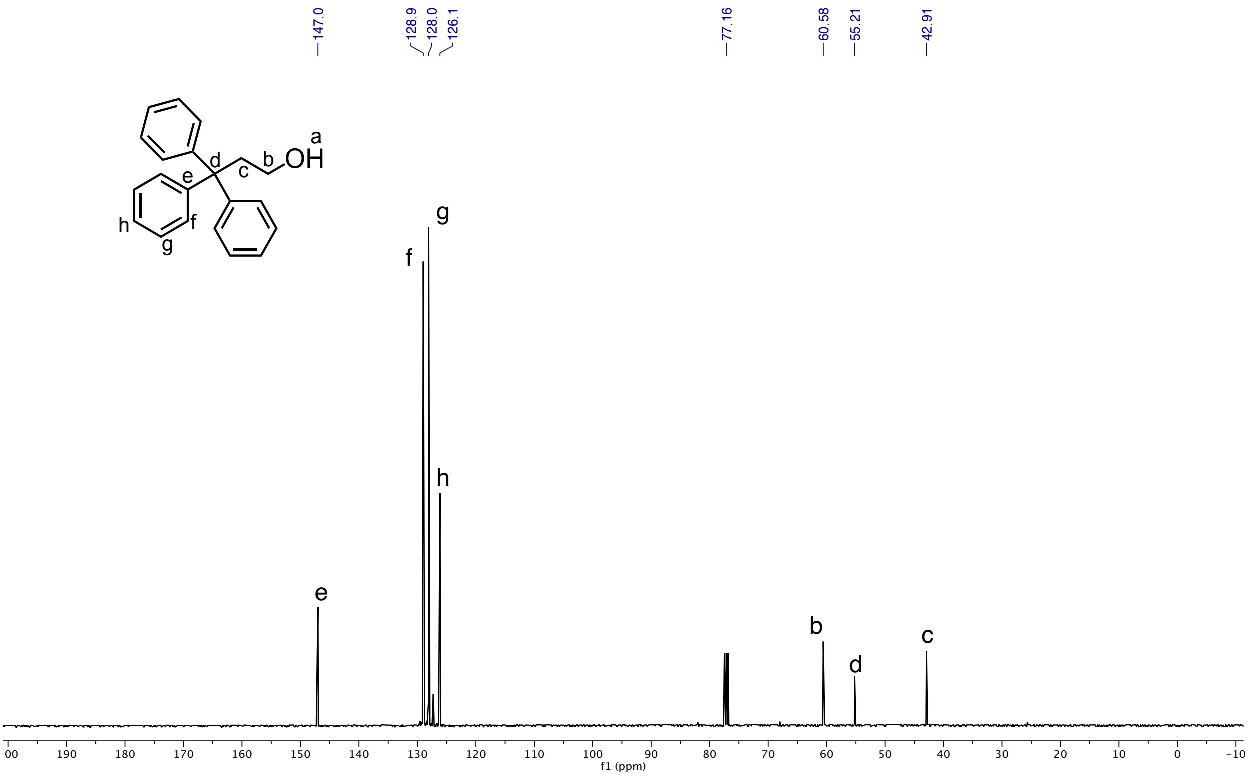

Figure S34. 101 MHz ^{13}C NMR spectrum of **39** in $CDCl_3$.

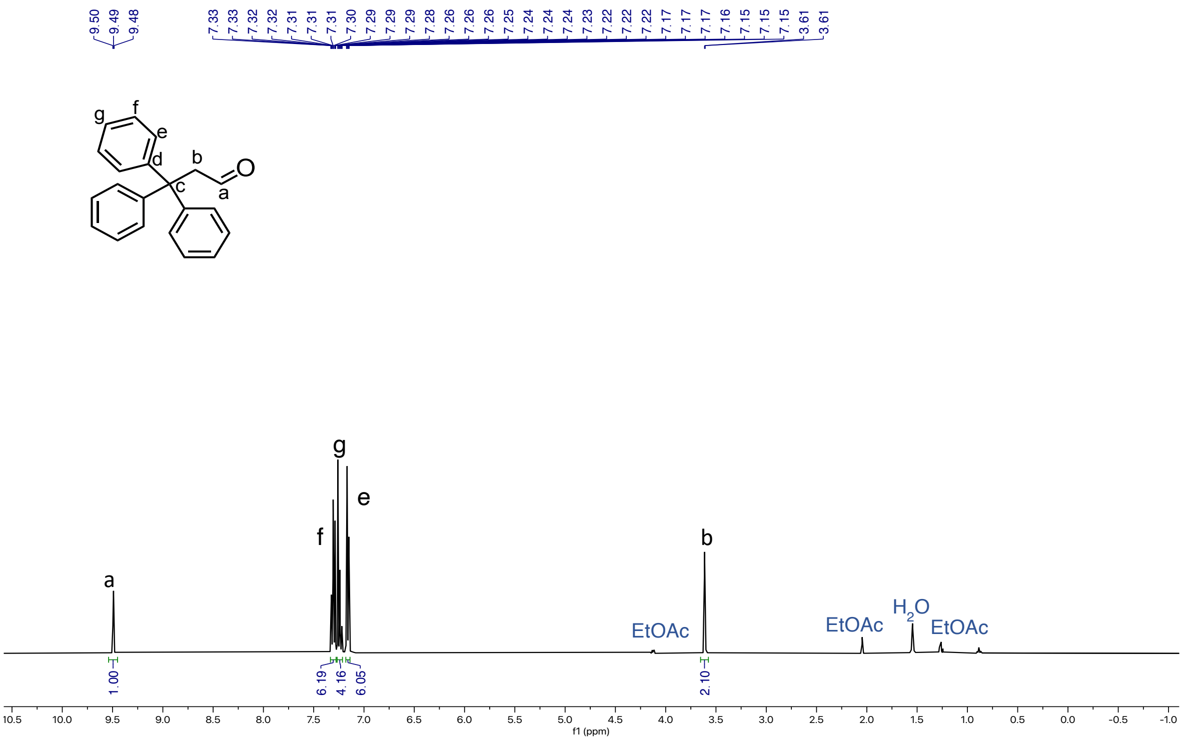

Figure S35. 400 MHz ^{1}H NMR spectrum of **40** in $CDCl_3$.

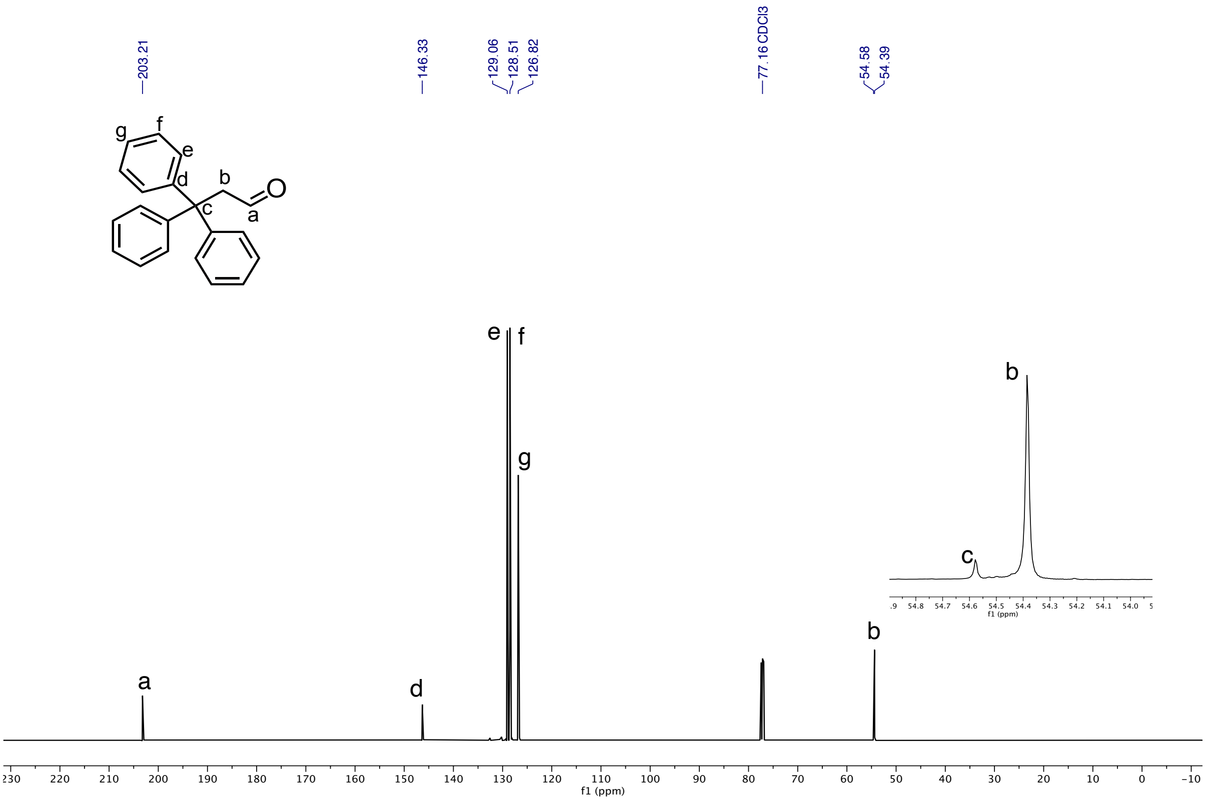

Figure S36. 126 MHz ^{13}C NMR spectrum of **40** in $CDCl_3$.

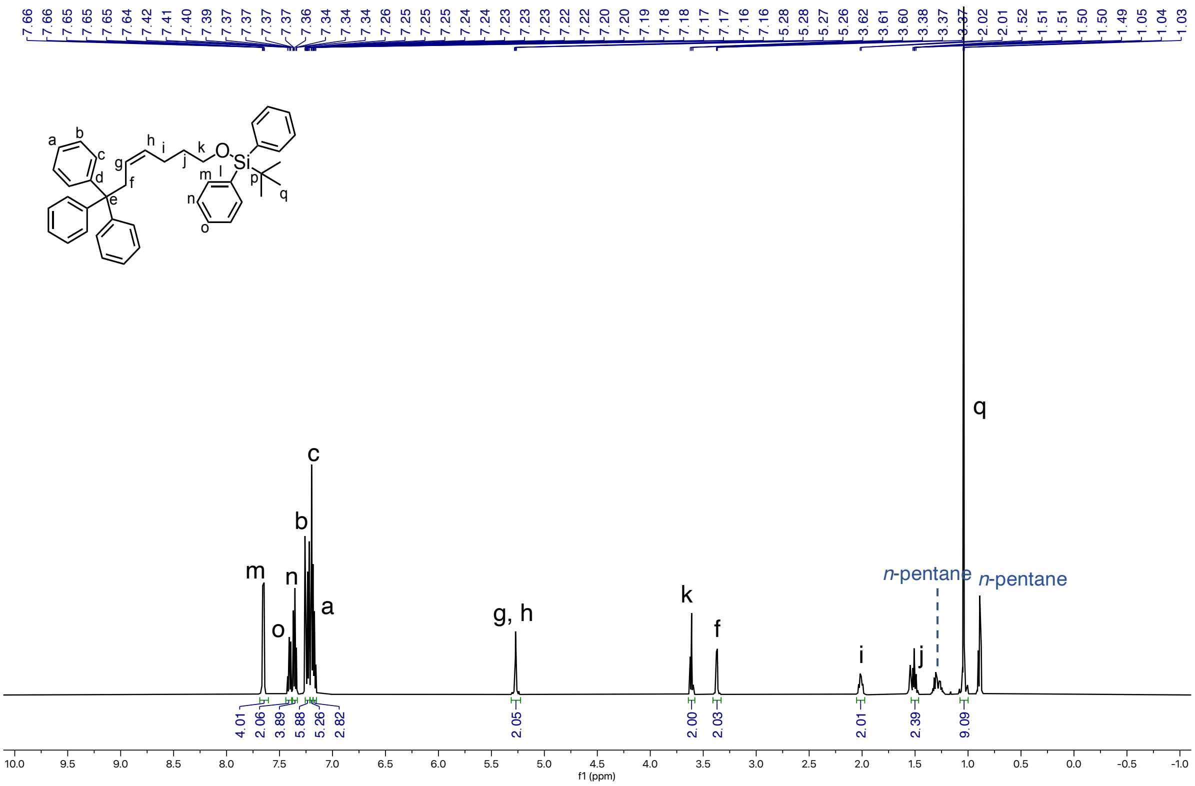

Figure S37. 500 MHz ^{1}H NMR spectrum of **41** in $CDCl_3$.

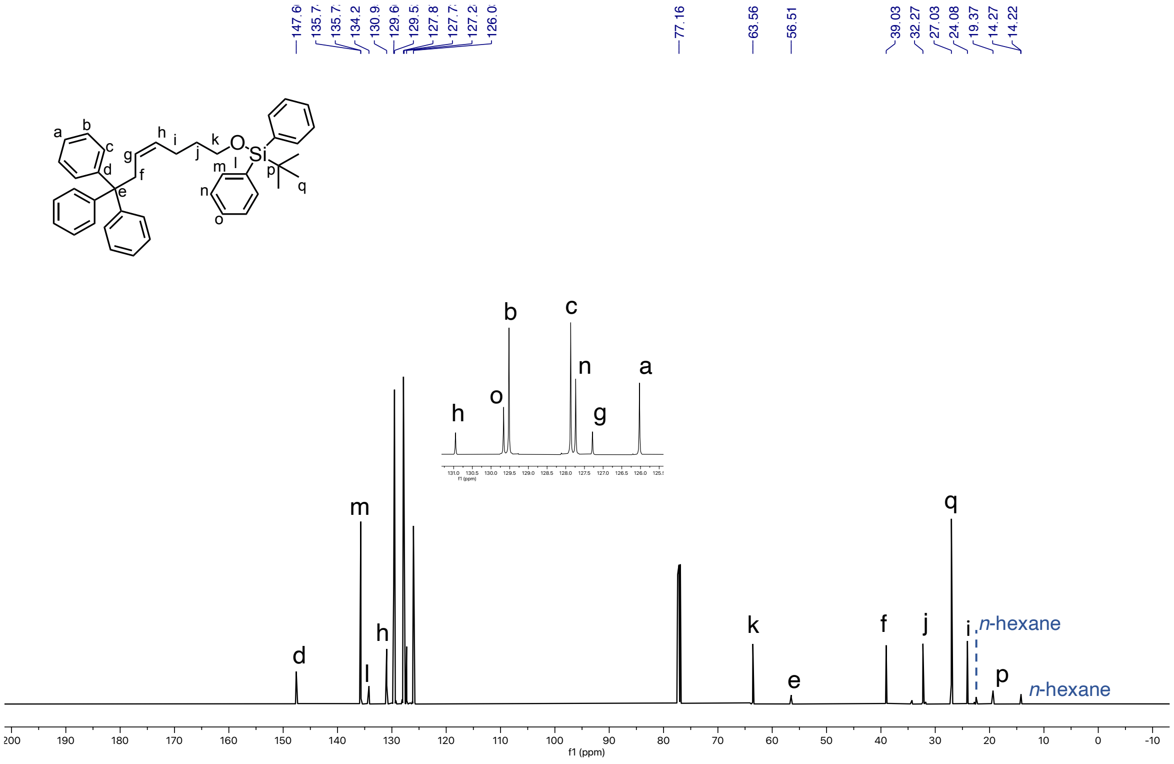

Figure S38. 126 MHz ^{13}C NMR spectrum of **41** in $CDCl_3$.

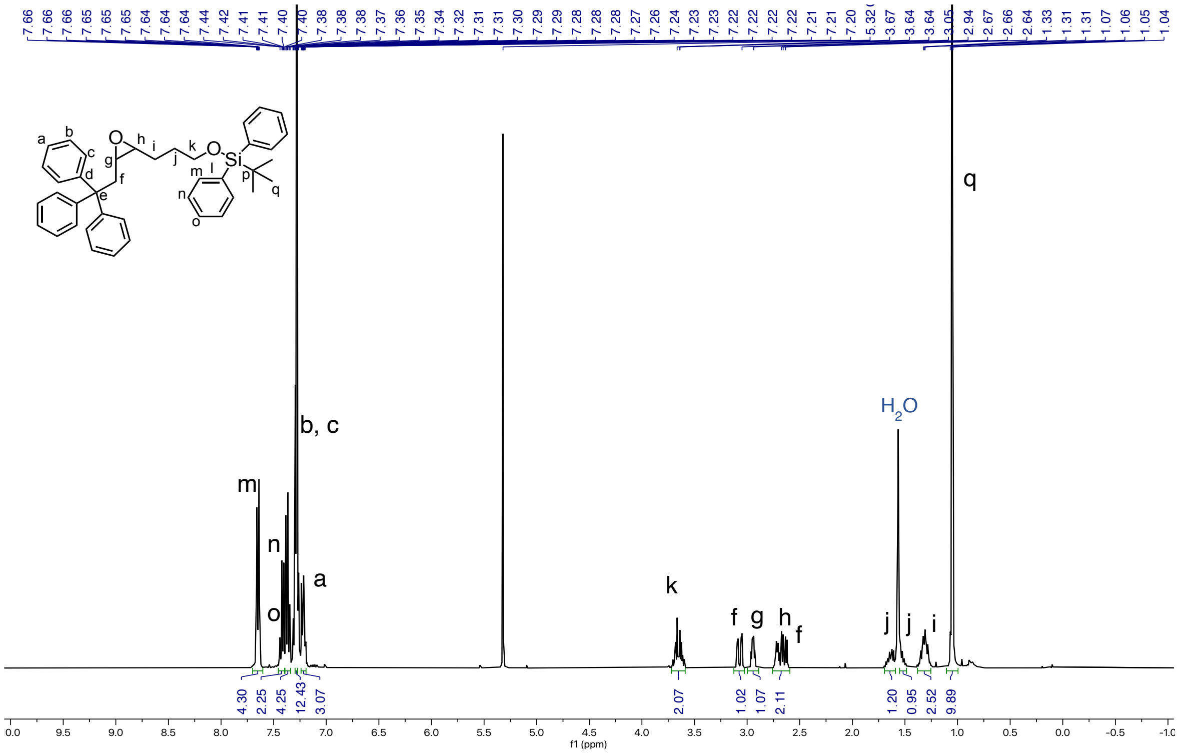

Figure S39. 400 MHz ^{1}H NMR spectrum of **42** in CD_2Cl_2.

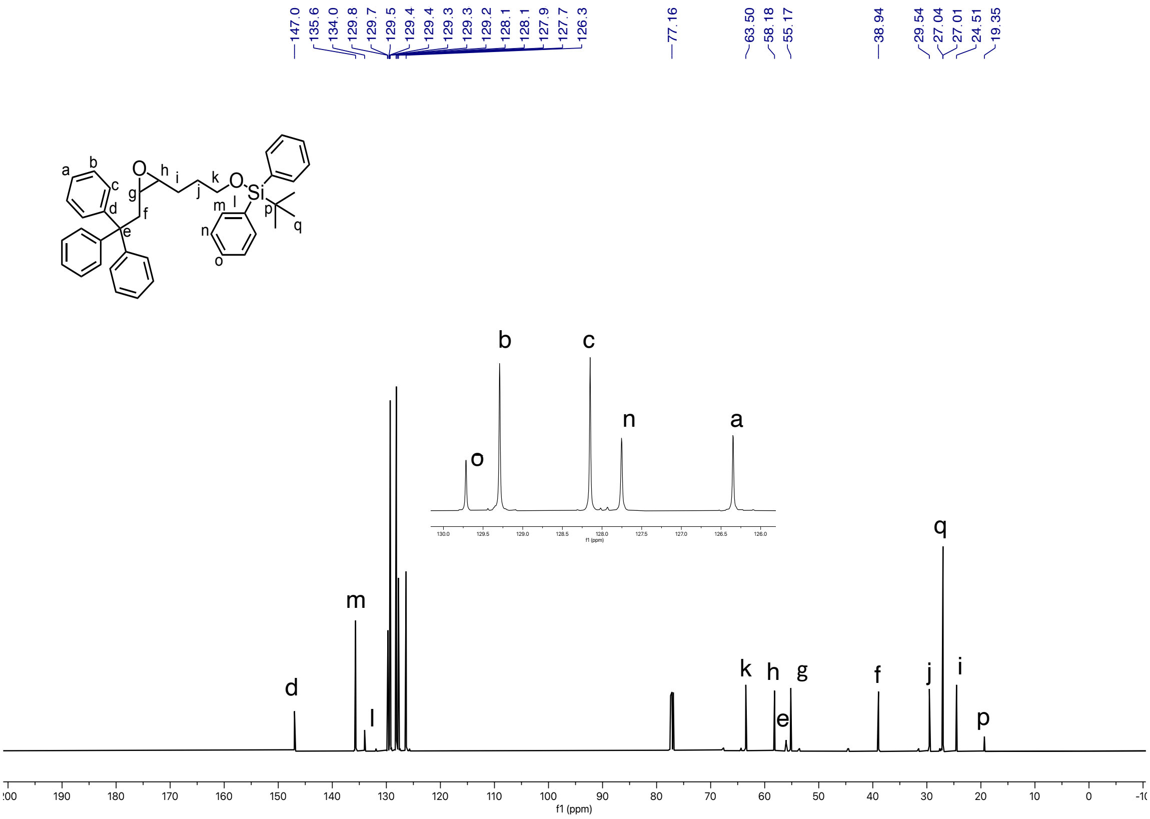

Figure S40. 126 MHz ^{13}C NMR spectrum of **42** in $CDCl_3$.

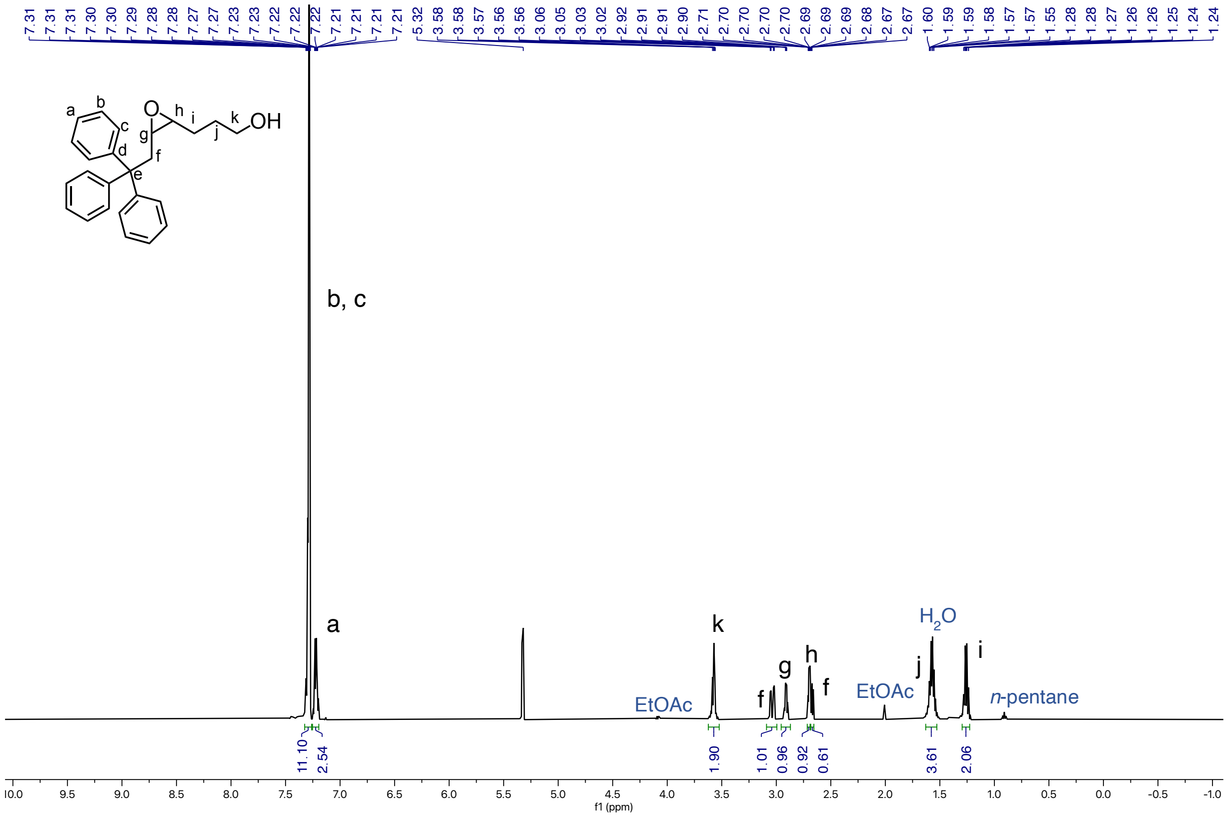

Figure S41. 500 MHz ^{1}H NMR spectrum of **21** in CD_2Cl_2.

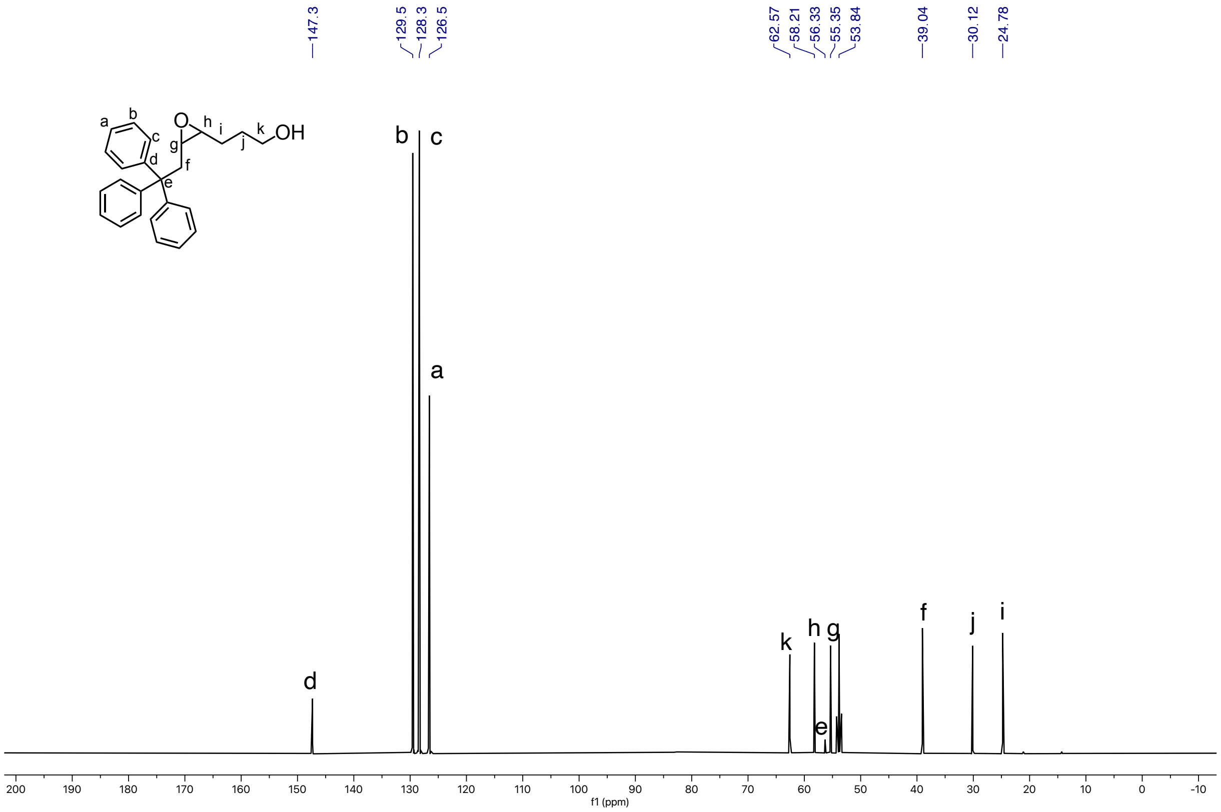

Figure S42. 126 MHz ^{13}C NMR spectrum of **21** in CD_2Cl_2.

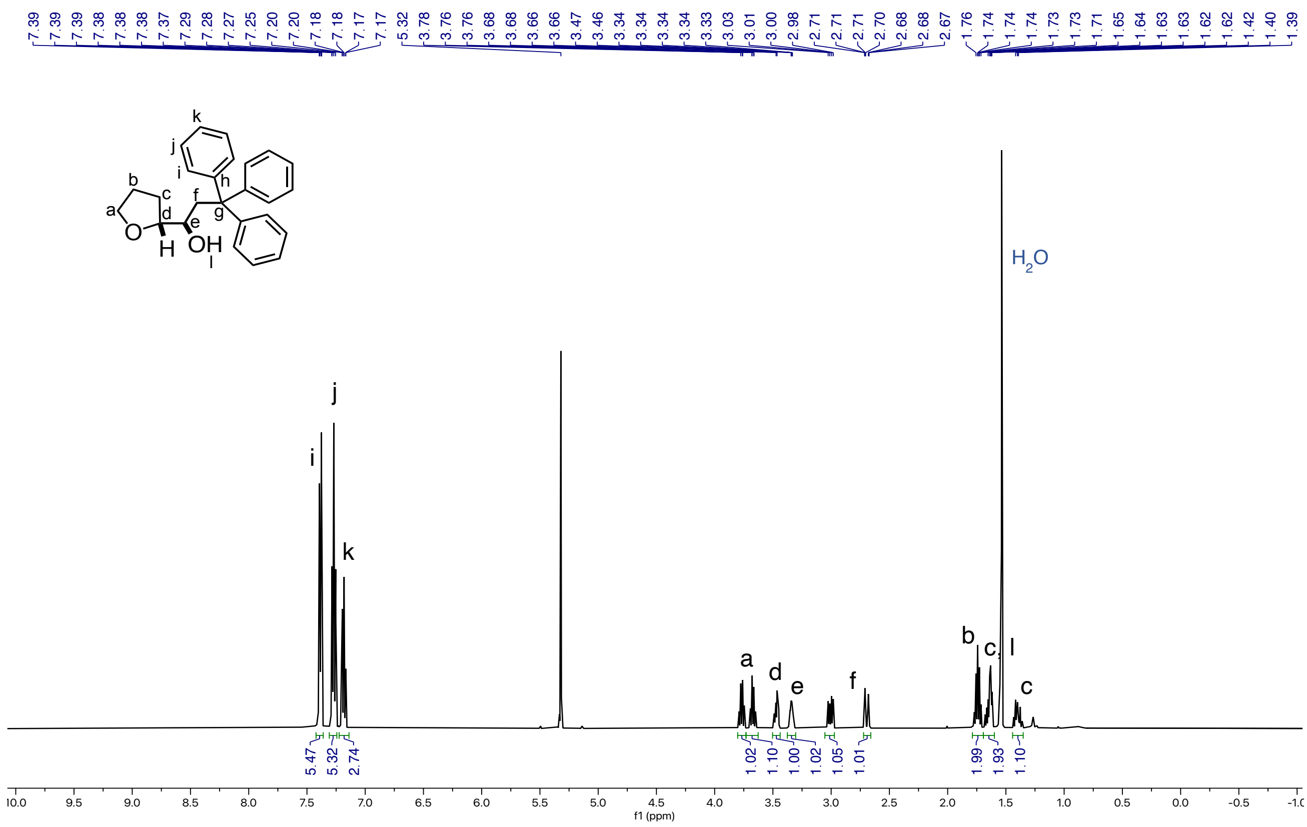

Figure S43. 500 MHz ^{1}H NMR spectrum of **24** in CD_2Cl_2.

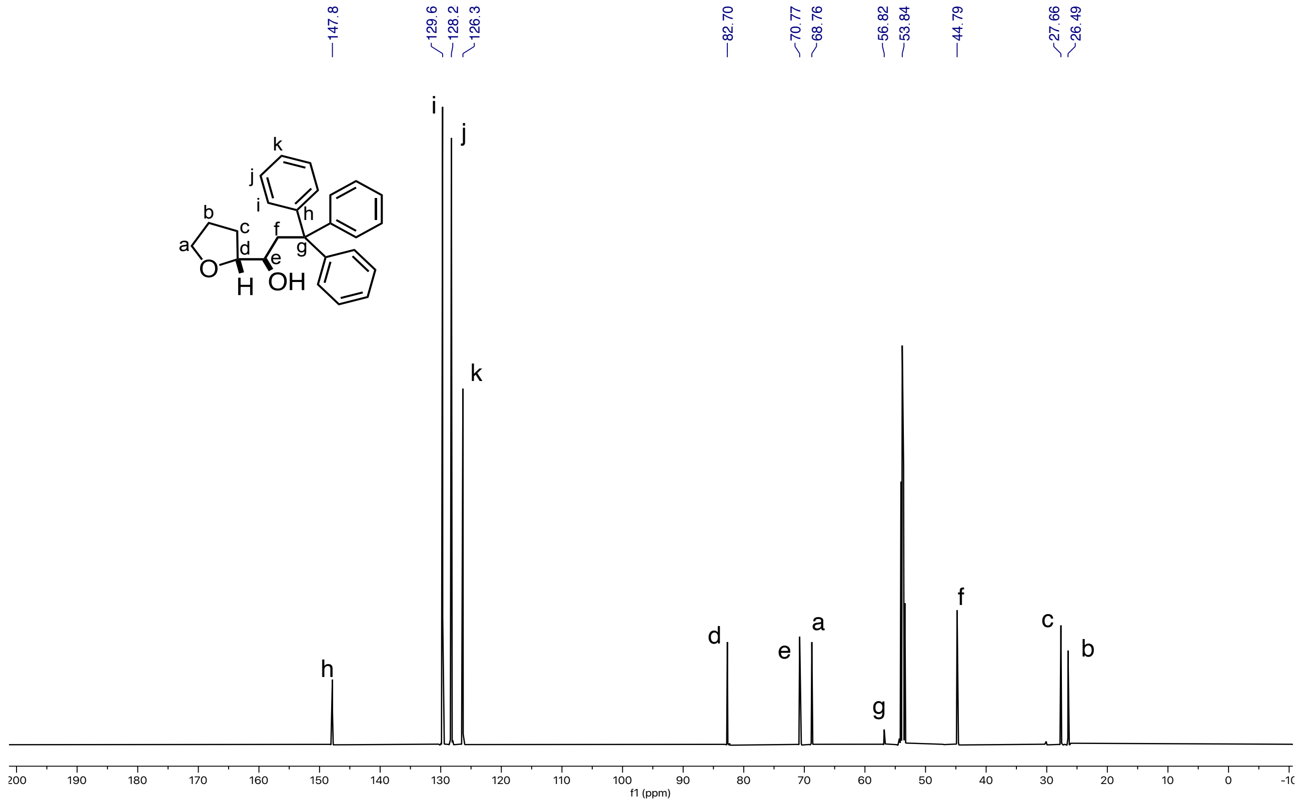

Figure S44. 126 MHz ^{13}C NMR spectrum of **6** in CD_2Cl_2.

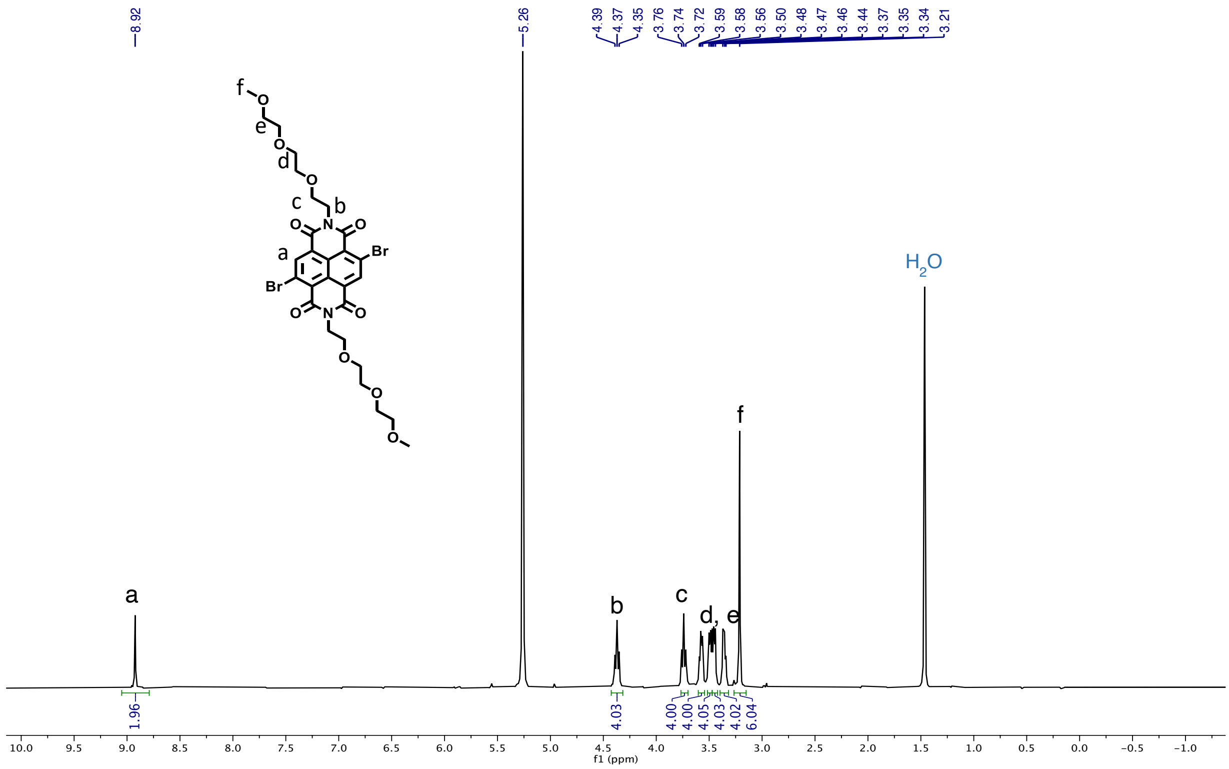

Figure S45. 300 MHz ^{1}H NMR spectrum of **45** in CD_2Cl_2.

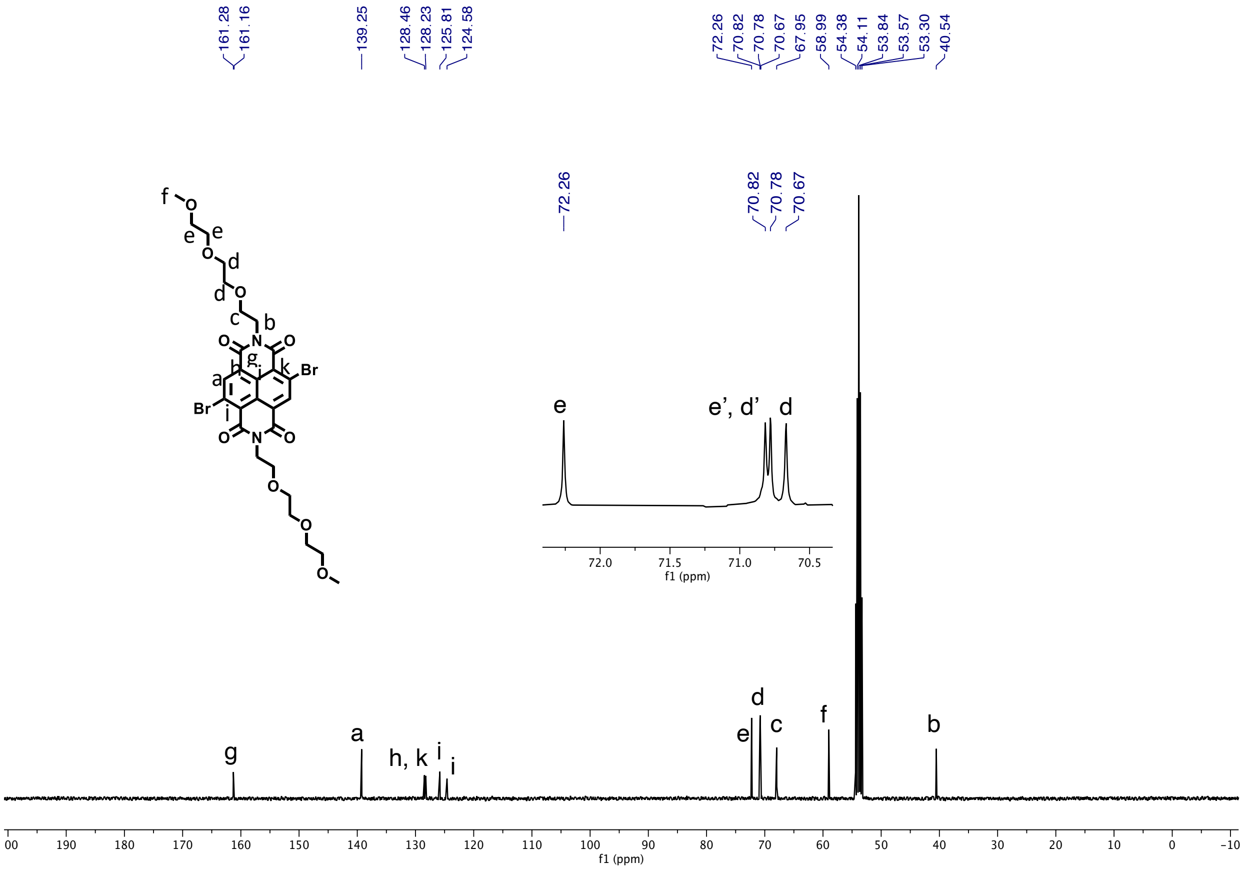

Figure S46. 101 MHz ^{13}C NMR spectrum of **45** in CD_2Cl_2.

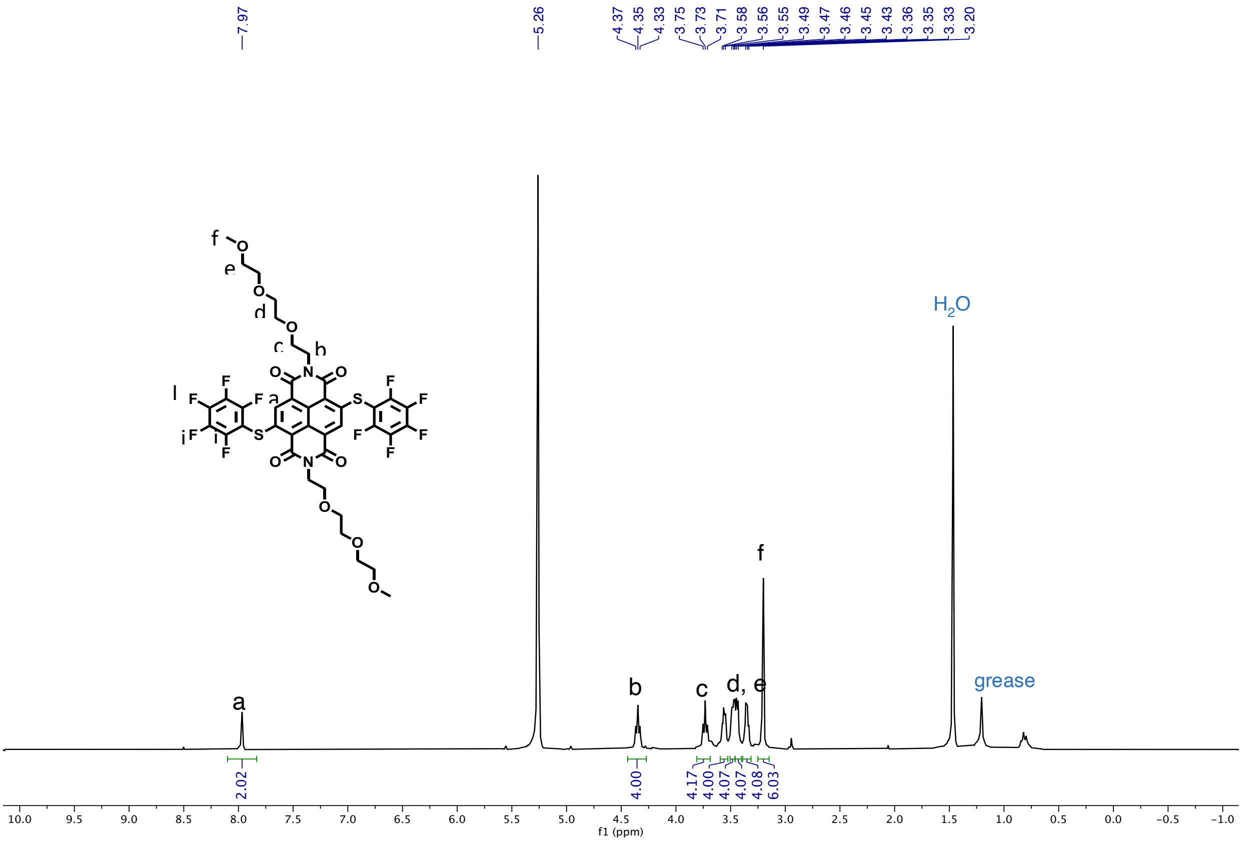

Figure S47. 300 MHz ^1H NMR spectrum of **47** in CD_2Cl_2

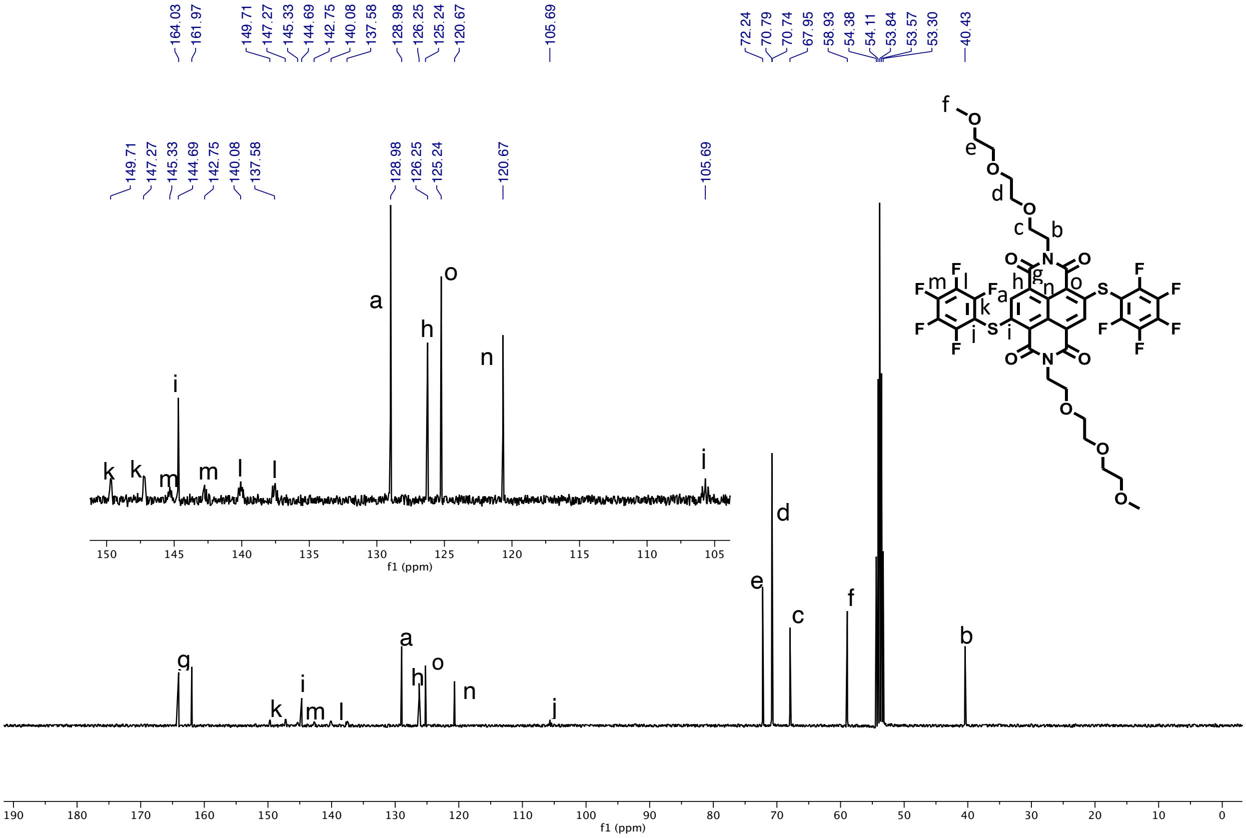

Figure S48. 101 MHz ^{13}C NMR spectrum of **47** in CD_2Cl_2.

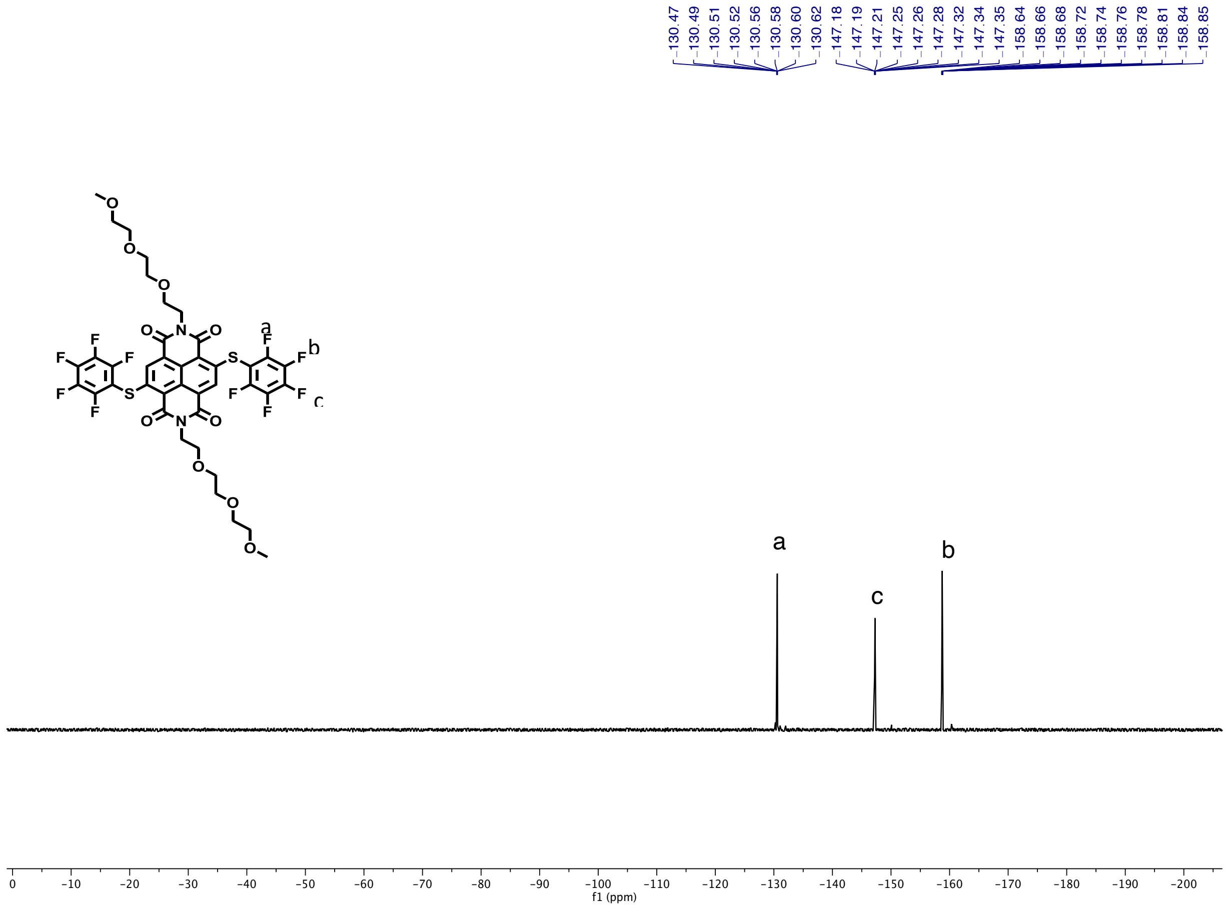

Figure S49. 282 MHz ^{19}F NMR spectrum of **47** in CD_2Cl_2.

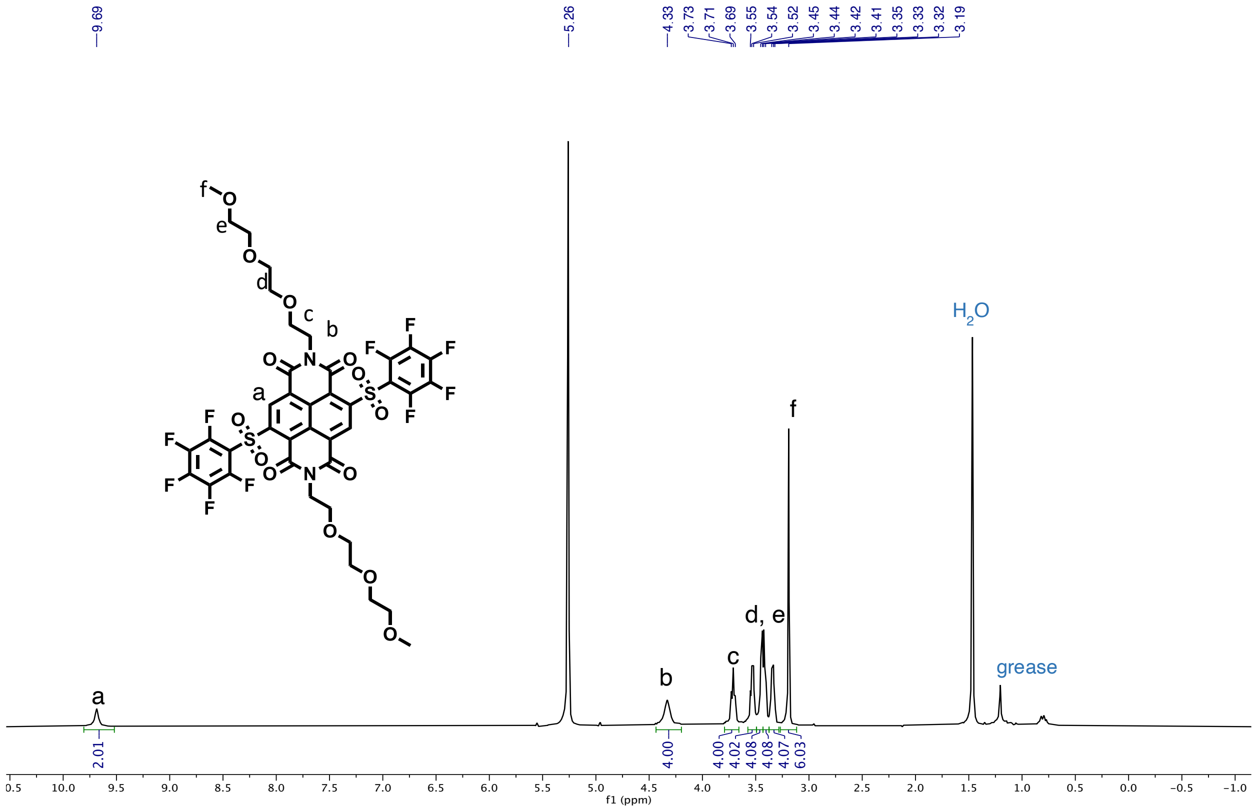

Figure S50. 300 MHz ^{1}H NMR spectrum of **35** in CD_2Cl_2.

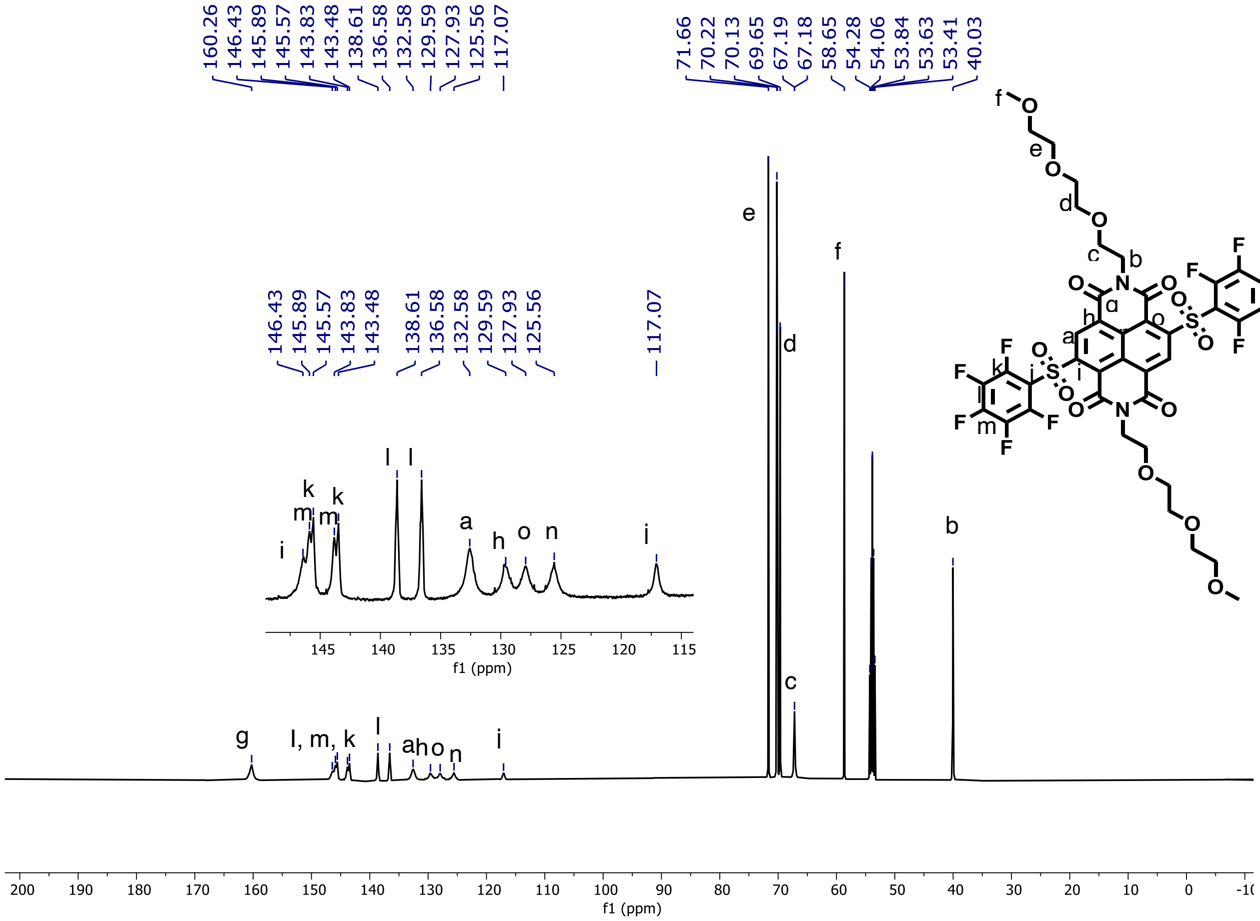

Figure S51. 126 MHz ^{13}C NMR spectrum of **35** in CD_2Cl_2 at -30 °C.

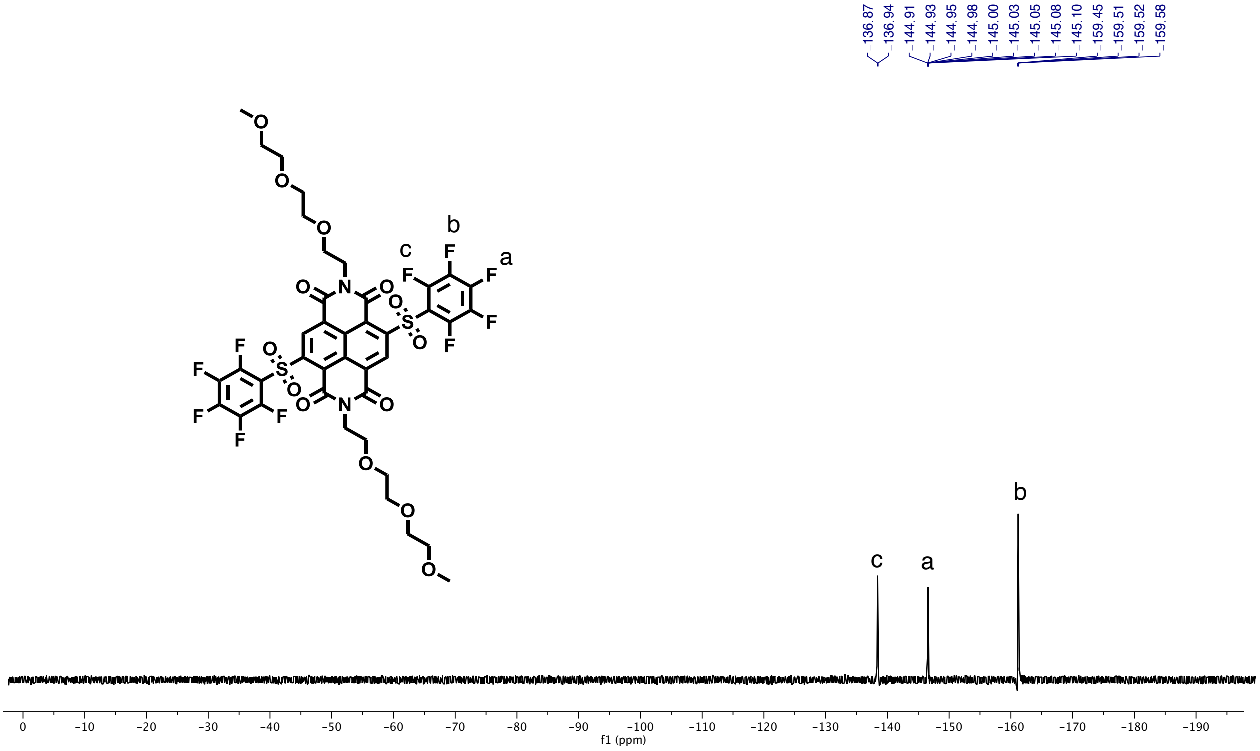

Figure S52. 282 MHz ^{19}F NMR spectrum of **35** in CD_2Cl_2.

6. Supporting References

(S1) Hao, X.; Li, T-R.; Chen, H.; Gini, A.; Zhang, X.; Rosset, S.; Mazet, C.; Tiefenbacher, K.; Matile, S. Bioinspired Ether Cyclizations within a π-Basic Capsule Compared to Autocatalysis on π-Acidic Surfaces and Pnictogen-Bonding Catalysts. *Chem. Eur. J.* **2021**, *27*, 12215-12223.

(S2) Lindborg, J.; Tanskanen, A.; Kanerva, L. T. Chemoselective Chloroperoxidase-Catalyzed Oxidation of Hexen-1-ols. *Biocatal. Biotransformation* **2009**, *27*, 204–210.

(S3) Gini, A.; Paraja, M.; Galmés, B.; Besnard, C.; Poblador-Bahamonde, A. I.; Sakai, N.; Frontera, A. Matile, S. Pnictogen-Bonding Catalysis: Brevetoxin-Type Polyether Cyclizations. *Chem. Sci.* **2020**, *11*, 7086–7091.

(S4) Le, L.; Matile, S. Anion-π Transaminase Mimics. *Supramol. Chem.* **2017**, *29*, 702–706.

(S5) Zhang, X.; Hao, X.; Le, L.; Pham, A.-T.; López-Andarias, J.; Frontera, A.; Sakai, N.; Matile, S. Primary Anion−π Catalysis and Autocatalysis. *J. Am. Chem. Soc.* **2018**, *140*, 17867–17871.

(S6) Lin, N.-T.; Vargas Jentzsch, A.; Guénée, L.; Neudörfl, J.-M.; Aziz, S.; Berkessel, A.; Orentas, E.; Sakai, N.; Matile, S. Enantioselective Self-Sorting on Planar, π-Acidic Surfaces of Chiral Anion-π Transporters. *Chem. Sci.* **2012**, *3*, 1121-1127.

(S7) Colobert, F.; Kreuzer, T.; Cossy, J.; Reymond, S.; Tsuchiya, T.; Ferrié, L.; Marko, I.-E.; Jourdain, P. Stereoselective Synthesis of the C(53)-C(67) Polyene Fragment of Amphidinol 3. *Synlett* **2007**, *15*, 2351-2354.

(S8) Morozova, V.; Skotnitzki, J.; Moriya, K.; Karaghiosoff, K.; Knochel, P. Preparation of Optically Enriched Secondary Alkyllithium and Alkylcopper Reagents—Synthesis of (−)-Lardolure and Siphonarienal. *Angew. Chem. Int. Ed.* **2018**, *57*, 5516.

(S9) Hellerman, L. The Preparation of 3-Triphenylethylamine. Rearrangement of 3-Triphenylpropionhydroxamic Acid. *J. Am. Chem. Soc.* **1927**, *49*, 7, 1735–1742.

(S10) (a) Feng, S.; Liu,C.; Xu, X.; . Liu, X.; Zhang, L.; Nian, Y.; Cao, Y. Siloxane-Terminated Side Chain Engineering of Acceptor Polymers Leading to Over 7% Power Conversion Efficiencies in All-Polymer Solar Cells. *ACS Macro Lett.* **2017**, *6*, 1310–1314. (b) Sasikumar, M.; Suseela, Y. V.; Govindaraju, T. Dibromohydantoin: A Convenient Brominating Reagent for 1,4,5,8-Naphthalenetetracarboxylic Dianhydride. *Asian J. Org. Chem.* **2013**, *2*, 779–785.

(S11) Fernando, M.-P.; Joaquin F., P.-B. The Kinetic Rate Law for Autocatalytic Reactions. *J. Chem. Educ.* **1987**, *64*, 925–927.

(S12) Schwartz, L. M. More on Autocatalytic Reaction Data Analysis. *J. Chem. Educ.* **1989**, *66*, 677.

(S13) Becke, A. D. Density-functional Thermochemistry. III. The Role of Exact Exchange. *J. Chem. Phys*. **1993**, *98*, 5648-5652.

(S14) Lee, C.; Yang, W.; Parr, R. G. Development of the Colle-Salvetti Correlation-Energy Formula into a Functional of the Electron Density. *Phys. Rev. B.* **1988**, *37*, 785-789.

(S15) Vosko, S. H.; Wilk, L.; Nusair, M. Accurate Spin-Dependent Electron Liquid Correlation Energies for Local Spin Density Calculations: A Critical Analysis. *Can. J. Phys*. **1980**, *58*, 1200-1211.

(S16) Stephens, P. J.; Devlin, F. J.; Chabalowski, C. F.; Frisch, M. J. Ab Initio Calculation of Vibrational Absorption and Circular Dichroism Spectra Using Density Functional Force Fields. *J. Phys. Chem*. **1994**, *98*, 11623 11627.

(S17) Grimme, S.; Antony, J.; Ehrlich, S.; Krieg, H. A Consistent and Accurate ab Initio Parametrization of Density Functional Dispersion Correction (DFT-D) for the 94 Elements H-Pu. *J. Chem. Phys*. **2010**, *132*, 154104.

(S18) Hehre, W. J.; Ditchfield, R.; Pople, J. A. Self—Consistent Molecular Orbital Methods. XII. Further Extensions of Gaussian-Type Basis Sets for Use in Molecular Orbital Studies of Organic Molecules. *J. Chem. Phys*. **1972**, *56*, 2257.

(S19) Tomasi, J.; Mennucci, B.; Cammi, R. Quantum Mechanical Continuum Solvation Models. *Chem. Rev.* **2005**, *105*, 2999-3093.

(S20) Gaussian 16, Revision C.01, Frisch, M. J.; Trucks, G. W.; Schlegel, H. B.; Scuseria, G. E.; Robb, M. A.; Cheeseman, J. R.; Scalmani, G.; Barone, V.; Petersson, G. A.; Nakatsuji, H.; Li, X.; Caricato, M.; Marenich, A. V.; Bloino, J.; Janesko, B. G.; Gomperts, R.; Mennucci, B.; Hratchian, H. P.; Ortiz, J. V.; Izmaylov, A. F.; Sonnenberg, J. L.; Williams-Young, D.; Ding, F.; Lipparini, F.; Egidi, F.; Goings, J.; Peng, B.; Petrone, A.; Henderson, T.; Ranasinghe, D.; Zakrzewski, V. G.; Gao, J.; Rega, N.; Zheng, G.; Liang, W.; Hada, M.; Ehara, M.; Toyota, K.; Fukuda, R.; Hasegawa, J.; Ishida, M.; Nakajima,T.; Honda, Y.; Kitao, O.; Nakai, H.; Vreven, T.; Throssell, K. J.; Montgomery, A.; Jr., Peralta, J. E.; Ogliaro, F.; Bearpark, M. J.; Heyd, J. J.; Brothers, E. N.; Kudin, K. N.; Staroverov, V. N.; Keith, T. A.; Kobayashi, R.; Normand, J.; Raghavachari, K.; Rendell, A. P.; Burant, J. C.; Iyengar, S. S.; Tomasi, J.; Cossi, M.; Millam, J. M.; Klene, M.; Adamo, C.; Cammi, R.; Ochterski, J. W.; Martin, R. L.; Morokuma, K.; Farkas, O.; Foresman, J. B.; Fox, D. J. Gaussian, Inc., Wallingford CT, 2016.

(S21) Marenich, A. V.; Cramer, C. J.; Truhlar, D. G. Universal Solvation Model Based on Solute Electron Density and on a Continuum Model of the Solvent Defined by the Bulk Dielectric Constant and Atomic Surface Tensions. *J. Phys. Chem. B*. **2009**, *113*, 6378-6396.

The original data that support the findings of this study are openly available:

https://doi.org/10.5281/zenodo.7575444
